# Cavity electrodynamics of van der Waals heterostructures

**DOI:** 10.1038/s41567-025-03064-8

**Published:** 2025-10-20

**Authors:** Gunda Kipp, Hope M. Bretscher, Benedikt Schulte, Dorothee Herrmann, Kateryna Kusyak, Matthew W. Day, Sivasruthi Kesavan, Toru Matsuyama, Xinyu Li, Sara Maria Langner, Jesse Hagelstein, Felix Sturm, Alexander M. Potts, Christian J. Eckhardt, Yunfei Huang, Kenji Watanabe, Takashi Taniguchi, Angel Rubio, Dante M. Kennes, Michael A. Sentef, Emmanuel Baudin, Guido Meier, Marios H. Michael, James W. McIver

**Affiliations:** 1 Max Planck Institute for the Structure and Dynamics of Matter, Hamburg, Germany; 2 Center for Free-Electron Laser Science, Hamburg, Germany; 3 Department of Physics, Columbia University, New York, NY USA; 4 Institut für Theorie der Statistischen Physik, RWTH Aachen University, JARA-Fundamentals of Future Information Technology, Aachen, Germany; 5 Research Center for Electronic and Optical Materials, National Institute for Materials Science, Tsukuba, Japan; 6 Research Center for Materials Nanoarchitectonics, National Institute for Materials Science, Tsukuba, Japan; 7 Initiative for Computation Catalysis, Center for Computational Quantum Physics, Simons Foundation Flatiron Institute, New York, NY USA; 8 Nano-Bio Spectroscopy Group, ETSF, Universidad del País Vasco, San Sebastián, Spain; 9 Institute for Theoretical Physics, Bremen Center for Computational Materials Science, University of Bremen, Bremen, Germany; 10 Laboratoire de Physique de l’Ecole Normale Supérieure, Université PSL, CNRS, Sorbonne Université, Université Paris-Cité, Paris, France; 11 Institut Universitaire de France, Paris, France

**Keywords:** Electronic properties and materials, Nanophotonics and plasmonics, Optical properties and devices

## Abstract

Van der Waals heterostructures host many-body quantum phenomena that are tunable in situ using electrostatic gates. Their constituent two-dimensional materials and gates can naturally form plasmonic self-cavities, confining light in standing waves of current density due to finite-size effects. The plasmonic resonances of typical graphite gates fall in the gigahertz to terahertz range, corresponding to the same microelectronvolt to millielectronvolt energy scale as the phenomena in van der Waals heterostructures that they electrically control. This raises the possibility that the built-in cavity modes of graphite gates are relevant for shaping the low-energy physics of these heterostructures. However, probing these cavity-coupled electrodynamics is challenging as devices are notably smaller than the diffraction limit at the relevant wavelengths. Here we report on the intrinsic cavity conductivity of gate-tunable graphene heterostructures. As the carrier density is tuned, we observe coupling and spectral weight transfer between graphene and graphite plasmonic cavity modes in the ultrastrong coupling regime. We present an analytical model to describe the results and provide general principles for cavity design. Our findings show that intrinsic cavity effects are important for understanding the low-energy electrodynamics of van der Waals heterostructures and open a pathway for useful functionality through cavity control.

## Abstract (web summary)

Integrating an electronic device with a cavity can cause the electrons to couple to photons strongly enough to form hybrid modes. Now, the cavity effects induced by intrinsic graphite gates are shown to modify the low-energy properties of graphene.

## Main

Many low-energy quantum phenomena have been observed in van der Waals (vdW) heterostructures, constructed by mechanically stacking and patterning exfoliated two-dimensional (2D) materials. Examples include superconductivity^1^, correlated insulating states^2^, polaritons^3,4^, 2D magnetism^5^ and both integer and fractional quantum anomalous Hall effects^6,7^. These effects are sensitive to their electromagnetic environment^8^ and can be tuned in situ using electrostatic gates, which control the material’s carrier density and electronic structure. We show here that exfoliated flakes of 2D materials can also form plasmonic self-cavities, confining terahertz (or millelectronvolt) light in standing waves of current density in the subwavelength regime due to finite size effects^9–11^. The plasmonic resonances of prototypical graphite gates (in the range of 0.25–2.5 THz) coincidentally fall on the same energy scale of many predicted collective modes and energy gaps in vdW heterostructures (in the range of 0.1–10 meV)^1,2,6,7,12^. This raises the question: can discrete modes of light confined in a plasmonic graphite gate modify or control the electrodynamics of a vdW heterostructure?

Engineering the properties of quantum materials using the enhanced light–matter interaction provided by a cavity has gained traction as a route to new functionality^13–19^. When the cavity coupling strength *g* approaches the cavity resonance frequency *ν*_0_, collective matter modes, such as phonons, plasmons or magnons, are transformed into light–matter hybrids, whose formation can modify the macroscopic properties of the material itself^13,14^, even in the presence of dissipation^20^. As the interaction increases, the light–matter coupling becomes non-perturbative. In the ultrastrong coupling regime, defined as *η* = *g*/*ν*_0_ ≳ 0.1, few photon drives, or even only photon vacuum fluctuations, can create new thermodynamic ground states^21,22^. Pioneering experiments have demonstrated an enhancement of ferromagnetism^23^, shifts in the critical temperature of metal–insulator transitions^24^ and the modification of both integer^25^ and fractional^26^ quantum Hall states in an empty cavity—all achieved with relatively low cavity quality factors on the order of 5.

The phenomena found in vdW heterostructures reflect the weakly formed long-range order in two dimensions, and thus small perturbations by plasmonic cavity modes could tip the balance between two competing phases with qualitatively different macroscopic responses^27^. In addition, the low energy scale of the emergent physics should make it easier to enter the ultrastrong light–matter coupling regime^18,21,22^. VdW heterostructures, with their built-in plasmonic cavity modes, are therefore good candidates for developing and testing cavity control protocols. The first step to doing so is capturing their intrinsic cavity-coupled electrodynamics. This requires measuring the frequency-dependent, complex terahertz conductivity of a gate-tunable heterostructure assembly and understanding the manifestation of finite-size effects on the electrodynamics. This presents experimental challenges, as far-field spectroscopic tools cannot operate on subwavelength-sized samples, and local near-field probes do not measure the global conductivity.

On-chip terahertz spectroscopy is a technique for investigating the terahertz response of vdW heterostructures^11,28–31^. By confining terahertz light to the near-field in metallic transmission lines interfaced to these micrometre-sized materials, the discrepancy between the free-space terahertz wavelength and small sample size can be overcome. This approach has been used to measure gate voltage-dependent changes in transmitted terahertz fields, from which estimates of the sample conductivity have been made. However, probing the conductivity of self-cavity modes and their hybridization in vdW heterostructures has not been possible so far due to circuitry limitations and the lack of a theoretical framework for understanding the impact of finite-size effects on the terahertz response.

**Fig. 1:**
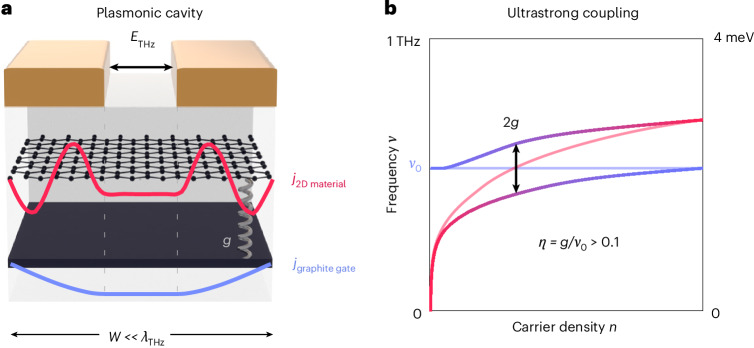
VdW heterostructure illustrating ultrastrong coupling between the sample and gate plasmonic self-cavity modes. **a**, A schematic of a subwavelength-sized (*W* ≪ *λ*_THz_) vdW heterostructure consisting of a 2D material and a few-nanometre-thick graphite gate embedded in a micropatterned dielectric environment provided by a metallic transmission line. A plasmonic self-cavity mode of a 2D material (red), such as a graphene plasmon studied in this work, can hybridize with the plasmonic self-cavity mode of the graphite gate (blue). **b**, The frequency of the screened plasmonic mode in the 2D material (light-magenta line) is tuned with carrier density into resonance with the screened graphite mode, *ν*_0_ (light-blue line). The self-cavity modes of graphene and graphite hybridize due to coupling mediated by unscreened currents between the metal strips and an avoided crossing appears (Supplementary Section 0.9), with an energy splitting of twice the coupling strength, *g*. When *η*, the ratio of *g*/*ν*_0_, exceeds the value of 0.1, the system is in the ultrastrong coupling regime^21,22^.

Here, we report on the cavity electrodynamics of gate-tunable vdW heterostructures by delivering a terahertz circuitry architecture and methodology for performing on-chip terahertz spectroscopy. We extracted the cavity conductivity of monolayer graphene heterostructures with graphite gates and found that plasmonic self-cavity modes form both in the graphene and graphite layers (Fig. 1). We observed distorted carrier-density dependencies of plasmon modes and spectral weight transfer from the graphite cavity mode to multiple graphene modes, demonstrating their hybridization. We quantified the normalized coupling strength of this hybridization as *η* = *g*/*ν*_0_ > 0.1, accessing the ultrastrong light–matter interaction regime. We developed an analytical theory that accounts for the geometry and dielectric environment of vdW heterostructures in their terahertz response, which reproduces the results of both numerical simulations and experimental data. This non-perturbative theory enables us to identify the coupling mechanism between two modes and provides generalizable design principles for enhancing or minimizing cavity coupling.

We find that self-cavity and cavity-coupling effects are intrinsically present in vdW heterostructures. This indicates the need for determining how cavity modes affect the properties of vdW materials and raises the possibility of intentionally engineering cavities to control quantum phases. These results deliver a pathway to realize collective quantum phenomena and new functionality, such as Bose–Einstein condensation of plasmons^32^, polariton condensation^33,34^ or single photon detection in the terahertz regime^35^. By providing a chip-scale platform that enables contact-free measurements of the complex terahertz cavity conductivity of vdW heterostructures, deterministic tuning of light–matter interactions and spectral read-out of coupling strength, this work presents an experimental route for investigating and controlling vdW heterostructures via cavity dynamics.

## Cavity design and spectral readout

**Fig. 2:**
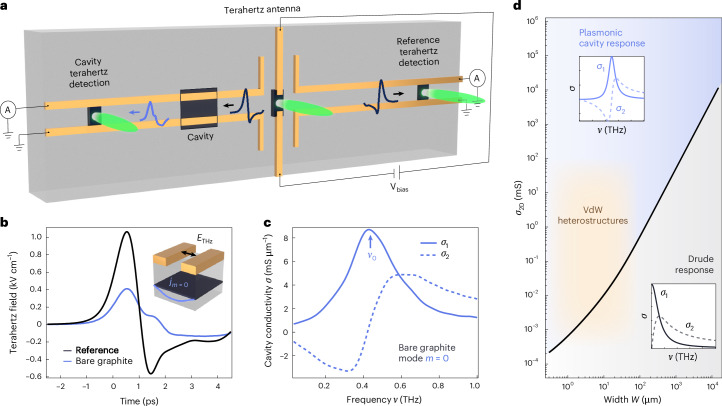
On-chip terahertz spectroscopy of plasmonic self-cavity modes. **a**, The circuit design for on-chip terahertz spectroscopy of vdW heterostructures: Two identical terahertz pulses (dark blue) are emitted from a voltage-biased terahertz antenna and coupled into two symmetric coplanar strip transmission line arms. The terahertz pulse interacts with the graphite cavity (dark → light blue) and is measured using a photoconductive switch triggered by a delayed probe beam on the left. The signal can be compared withthe reference signal measured on the detector on the right. **b**, Experimentally measured terahertz transients of a typical bare graphite plasmonic self-cavity (without an additional 2D material) and reference as a function of time delay. The detected cavity signal is notably modified as compared with the reference trace. **c**, Real and imaginary parts of the near-field cavity conductivity obtained from the data in **b**. **d**, Analytical theory simulation mapping where a plasmonic cavity or Drude response is expected as a function of *σ*_2D_ and total sample width *W* (with equally spaced metal strips that make up the transmission line, and *d*_hBN_ = 25 nm). hBN encapsulated vdW heterostructures typically fall in the cavity response regime due to finite-size effects, regardless of the presence of transmission lines. The boundary between regimes indicates where the measured cavity conductivity can be reliably fit with a Drude model (see Supplementary Fig. S18 for details). Cavity parameters (see also Extended Data Table 1 and Extended Data Fig. 1): *W*_0_ = 1.9 μm (graphite width exceeding the metal strips), *W*_1_ = 3.55 μm (width of each metal trace) and *W*_2_ = 2.26 μm (width of the gap between strips), *d*_gr_ = 16 nm (graphite thickness) and *d*_hBN_ = 26 nm (hBN thickness). Source data

**Extended Data Fig. 1:**
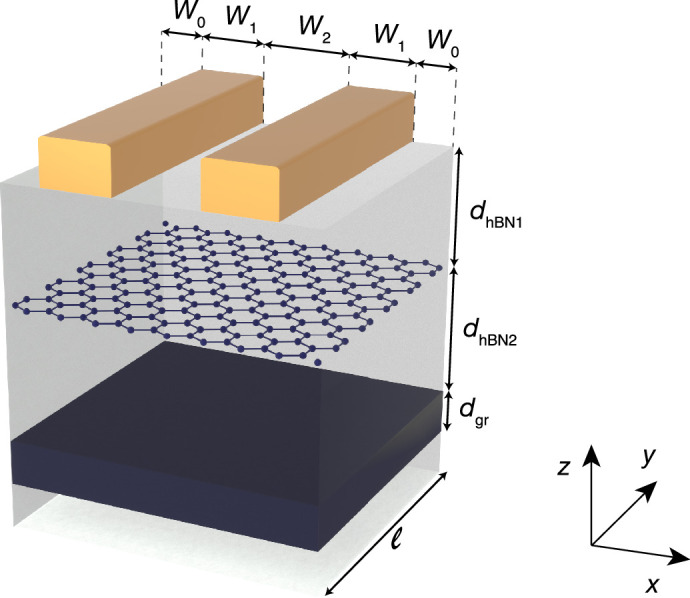
Definition of cavity geometry dimensions. Schematic illustrating parameters relevant for the device geometry.

**Extended Data Fig. 2:**
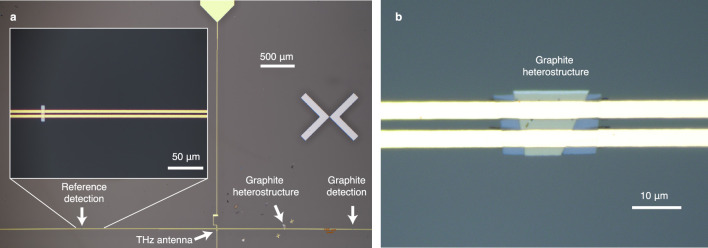
Optical micrograph of Device 1 discussed in Fig. 2. **a** Overview of the circuitry: THz pulses generated using the THz antenna couple to the left into an empty reference transmission line and to the right into the transmission line with the bare graphite cavity. Top left inset shows the reference detection switch. **b** Optical micrograph of the well-defined bare graphite cavity.

The cavities reported here combine vdW heterostructure devices of typical dimensions of the order 10 × 10 μm^2^ interfaced with circuitry for performing on-chip terahertz spectroscopy^10,36–41^. Each cavity was constructed using a precision-cut graphite flake, encapsulated in hexagonal boron nitride (hBN) and placed on a sapphire substrate (see Extended Data Table 1 and Extended Data Fig. 1 for precise cavity dimensions, and Extended Data Fig. 2 for a micrograph of the device). The hBN preserves the intrinsic properties, such as high mobility, of the 2D material from the deleterious effect of the environment and electrically insulates it from the circuitry. This circuitry confines light to the near-field, where the (*νλ*_near−field_)/*c* ≪ 1 (*c* is the speed of light), and enables spectral readout from 0.05 to 1 THz (refs. ^11,28,29^), within the terahertz gap^42^. The circuitry features a symmetric terahertz antenna, which injects a single odd mode into the coplanar strip transmission lines^43^, a requirement for probing the pure in-plane conductivity^44^. The use of highly repeatable evaporated silicon switches enables the introduction of in situ referencing circuitry, allowing the simultaneous measurement of the time-domain cavity and reference terahertz pulses on separate transmission line arms (Fig. 2a,b). The Fourier transforms of the recorded time-domain signals were then used to calculate the transmission coefficient, which in turn was used for numerically computing the real and imaginary parts of the heterostructure’s near-field optical conductivity, which we refer to as the cavity conductivity, plotted in Fig. 2c and Supplementary Fig. 2.

The experimental bare graphite self-cavity response in Fig. 2c exhibits a resonance at *ν*_0_ that is described by a Lorentz oscillator model and results from the excitation of a 2D plasmonic standing wave, which is predominantly confined beneath the metallic strips that form the transmission line. The electric field of the terahertz pulse generates a current in the graphite (Supplementary Fig. 9) that reflects at discontinuities in the dielectric environment, particularly at the sample boundaries, forming a standing wave of current density (*j*_*m*=0_ in Fig. 2b, inset). The wavelength and thus finite momentum of the confined plasmonic resonance are determined by the geometry and dielectric environment of the heterostructure^9–11,45,46^ (Supplementary Fig. 14).

We developed an analytical model to describe the plasmonic modes of the cavity and their conductivity^47^, whose results match with both experiment and finite element numerical simulations that capture the full electromagnetic field propagation through the cavity^48^ (Supplementary Figs. 10, 11 and 20). The analytical model enables predictions of the terahertz conductivity of micropatterned vdW heterostructures, facilitating the design of cavity modes within a target spectral range. It further highlights that vdW heterostructures of typical dimensions (of less than 100 μm) and 2D conductivities *σ*_2D_ exhibit cavity modes, in contrast to the far-field Drude response of metals (Fig. 2d). This insight underscores the need to incorporate the role of finite-size effects when interpreting on-chip terahertz spectroscopy data (Supplementary Section 0.5).

The terahertz circuitry and framework for understanding self-cavity effects offer multiple key functions. First, they enable contact-less, finite-momentum measurements of the complex cavity conductivity on the length-scales of typical vdW heterostructures, which are inaccessible with far-field techniques, local probes or photocurrent readout mechanisms^42^. The additional reference transmission line enables direct detection of the complex conductivity, an advancement over previous approaches that estimated conductivities by comparing gate-dependent changes in the transmitted terahertz field. Independent access to the real and imaginary parts of the conductivity, combined with an understanding of self-cavity effects, is necessary for measurements of parameters such as linewidths, mobilities and superfluid stiffness. In systems where electronic correlations shift spectral weight to high frequencies, the applicability of using Kramers–Kronig transformations of the absorption to extract complex quantities would be limited^42^. Finally, the metallic transmission lines provide sharp electrodynamic boundary conditions, breaking momentum conservation in a controllable manner that mediates the coupling between gate and sample plasmonic self-cavity modes, as elaborated below.

## Spectrally resolving gate-tunable graphene plasmonic modes

**Fig. 3:**
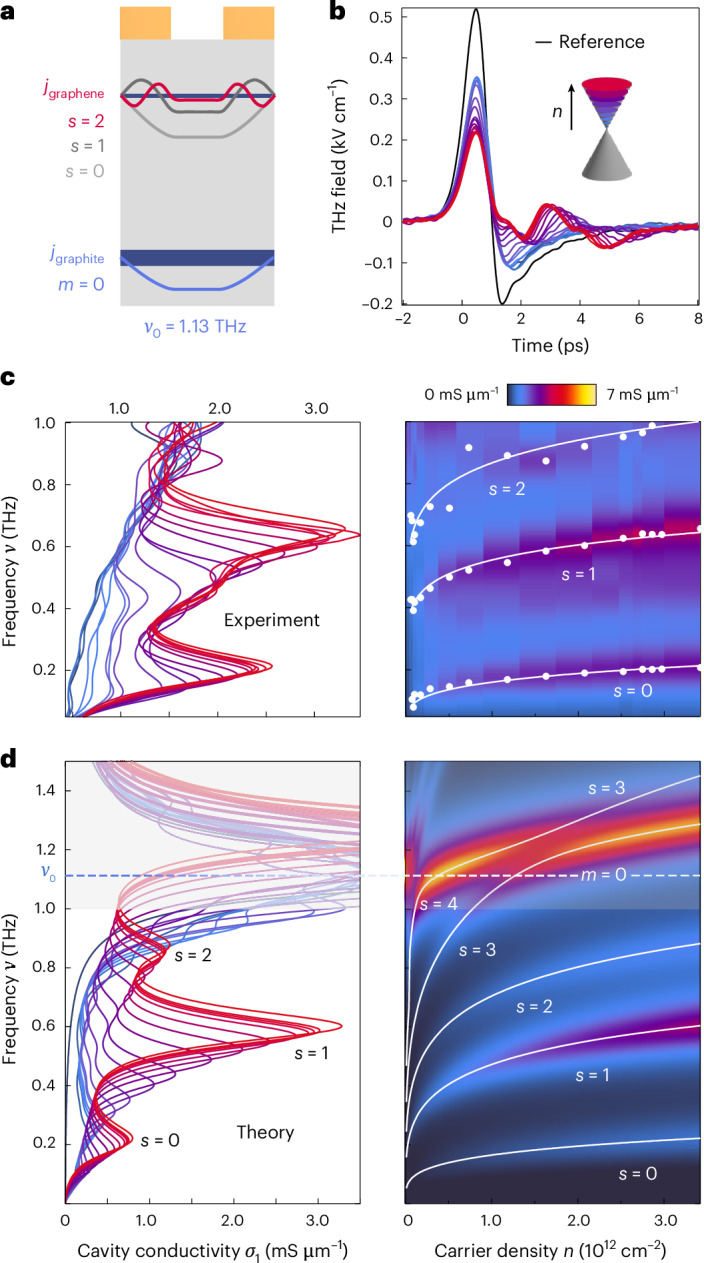
Probing the spectra of gate-tunable graphene plasmonic modes. **a**, Cavity design for characterizing the plasmonic self-cavity modes of a graphene layer (*s* = 0, 1, 2) with a resonance frequency of the graphite plasmonic self-cavity (*m* = 0) mode, *ν*_0_, of ~1.13 THz, outside the experimental spectral sensitivity range. **b**, Experimentally measured terahertz transients of the reference (black line) and increasing graphene carrier densities (coloured lines). **c**, Left: experimentally obtained real part of the cavity conductivity spectra. The graphite self-cavity conductivity (dark-blue trace) increases slowly with increasing frequency and is expected to peak at ~1.13 THz. Right: the resonance frequencies as a function of graphene carrier density of these three resonances. **d**, The analytically calculated cavity conductivity for the device shown in **a**, identifying the three experimentally detected resonances as the *s* = 0, 1, 2 graphene modes. The extended frequency range of the theory captures the avoided crossings between the *s* = 3, 4 modes to the graphite self-cavity mode (dashed line). Cavity parameters for device 2: *W*_0_ = 2.0 μm, *W*_1_ = 2.80 μm and *W*_2_ = 1.91 μm, *d*_gr_ = 17 nm, *d*_hBN1_ = 23 nm and *d*_hBN2_ = 91 nm. Source data

**Extended Data Fig. 3:**
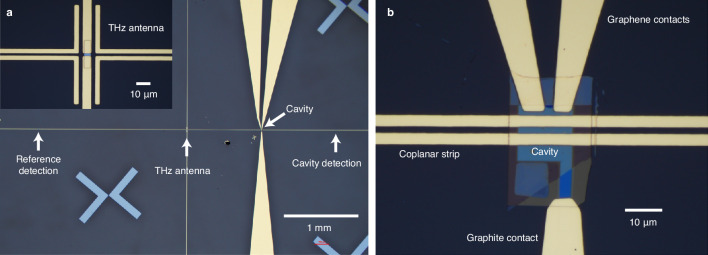
Optical micrograph of Device 2 discussed in Fig. 3. **a** Overview of the circuitry with a graphene cavity embedded in the transmission line on the right. Top left inset shows a magnified picture of the THz antenna. **b** Optical micrograph of the cavity with edge contacts to the graphite (bottom contact) and to the graphene (other two contacts). The close proximity of the contacts to the gold traces were not observed to play any role in the observed conductivities.

We investigated the behaviour of the plasmonic cavity modes of hBN-encapsulated graphene with an electrostatic graphite gate. We designed the device (Extended Data Table 1 and Extended Data Fig. 3) such that the graphite cavity resonance frequency was centred at approximately 1.13 THz, outside of the experimental spectral sensitivity range, to facilitate the spectral characterization of individual graphene plasmonic cavity modes (Fig. 3a). On-chip terahertz data taken at 20 K show that the transmission decreases as the sample reflectivity increases with increasing carrier density (Fig. 3b). In addition, time-domain oscillations appear, indicating a frequency-dependent optoelectronic response.

The real (dissipative) part of the cavity conductivity of a gate-tunable monolayer heterostructure was calculated from the time-domain data in Fig. 3b (see Supplementary Fig. 3 for Fourier transforms). The results are plotted in Fig. 3c. At the lowest carrier density, close to charge neutrality, the cavity conductivity data grow slowly with increasing frequency. This is consistent with the expectation that charge neutral graphene is largely transparent to terahertz fields. Only the rising edge of the graphite plasmonic self-cavity resonance is captured, which peaks outside the experimentally accessible spectral range.

As the graphene becomes more conductive, three resonances appear on top of this background, which grow in amplitude and blueshift as the carrier density increases. This can be explained as the formation of plasmonic self-cavity modes in the graphene (*j*_graphene_)^9–11,49,50^. Higher-frequency resonances, represented schematically in Fig. 3a, originate from higher-order plasmonic cavity modes. The labelling *s* = 0, 1, 2 corresponds to the number of nodes in the current density beneath each metal strip of the transmission line, as determined from finite element electromagnetic field simulations (Supplementary Fig. 8). The spectra in Fig. 3c were fit with a sum of Lorentzians to extract the linewidth and resonance frequency of each mode as a function of carrier density. A representative linewidth for the *s* = 2 mode at *n* = 2.85 × 10^12^ cm^−2^ corresponds to an electronic mean free path of approximately (9.1 ± 0.2) μm, roughly the width of the device (Supplementary Fig. 4). The quality factor is *Q* ≈ 5.7 ± 0.1, defined as *Q* ≈ *ν*_*s*=2_/*γ*_*s*=2_, where *ν*_*s*=2_ is the resonance frequency and *γ*_*s*=2_ is the linewidth. The scattering rate and quality factor are limited by device dimensions, illustrating that the graphene in the microstructured cavity exhibited ballistic transport, thereby achieving the highest possible quality factor for a device of this geometry in this frequency range.

In an uncoupled system and in the absence of electronic correlations, the carrier-density dependence of the resonance frequency is determined by the electronic band dispersion of the sample^51^. This can be monitored by tracking the resonance frequency extracted from fits as a function of carrier density *n*. We find that the *s* = 0 mode scales as *ν* ∝ *n*^0.23±0.03^, in good agreement with the expectation of a Dirac band structure, where 2D plasmons scale as *ν* ∝ *n*^0.25^ (ref. ^51^). However, the higher-order plasmon modes, *s* = 1, 2 scale as 0.12 ± 0.01 and 0.11 ± 0.01, respectively.

To understand the suppressed power laws of the *s* = 1, 2 modes, we included the contribution from the graphene layer in our electromagnetic calculations (Supplementary Fig. 12). Figure 3d plots the results of the real part of the simulated cavity conductivity up to 1.5 THz, using similar linewidths as extracted in experiment. Three modes appear below 1 THz with good agreement to the experimental data. Examining the simulated conductivity outside of the experimental range shows that, when the graphene *s* = 3, 4 modes cross with the graphite self-cavity mode (Fig. 3d, dashed line), they hybridize and form avoided crossings, splitting into upper and lower polariton branches. The simulations reveal that, at high carrier densities, the observed *s* = 1, 2 modes also form lower branches of avoided crossings occurring at carrier densities outside of the experimentally accessible range. The experimentally detected, suppressed power-law behaviours of the graphene *s* = 1, 2 modes are thus a consequence of the hybridization of graphene and graphite plasmons (Supplementary Fig. 21).

## Ultrastrong cavity coupling of plasmonic collective modes

**Fig. 4:**
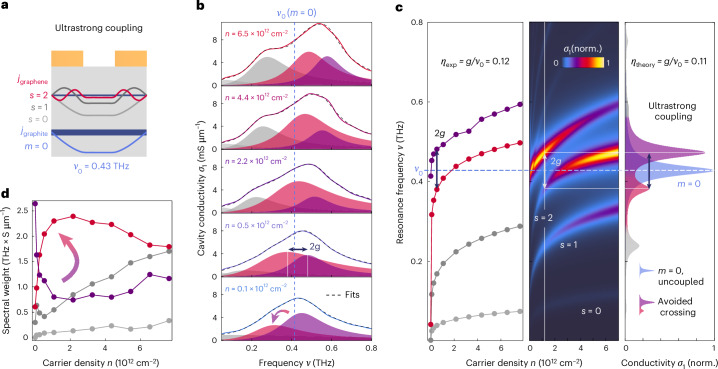
Ultrastrong coupling between graphene and graphite plasmonic self-cavity modes. **a**, Cavity design with a thin hBN layer between graphene and graphite to centre the graphite plasmonic self-cavity mode at 0.43 THz. The graphene *s* = 2 mode couples in the ultrastrong regime to the graphite *m* = 0 mode inside the experimental spectral sensitivity range. **b**, Experimentally obtained real part of the near-field terahertz conductivity spectra of a selection of measured graphene carrier densities (Supplementary Fig. 5). The data were fit to a sum of four Lorentzians as indicated by the shaded peaks. **c** Left: detected resonance frequencies as a function of carrier density for the four Lorentzians fit of the experimental data. The experimentally extracted normalized (norm.) coupling strength, ${\eta }_{\exp }=0.12\pm 0.01$, is in the ultrastrong coupling regime. Middle: the analytical calculation for this device follows similar trends, with *η*_theory_ = 0.11 ± 0.01. Right: the simulated conductivity spectra at a carrier density of *n* = 0, for the uncoupled *m* = 0 mode and for a carrier density of *n* = 1.24 × 10^12^ cm^−2^ at which the avoided crossing appears. **d**, Quantification of experimental spectral weight transfer between the two highest-frequency modes. Cavity parameters: *W*_0_ = 2.2 μm, *W*_1_ = 3.33 μm and *W*_2_ = 2.60 μm, *d*_gr_ = 10 nm, *d*_hBN1_ = 21 nm and *d*_hBN2_ = 18 nm. Source data

**Extended Data Fig. 4:**
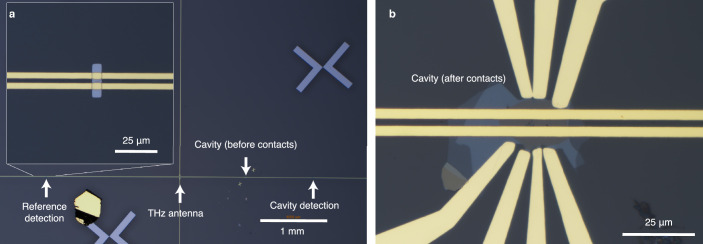
Optical micrograph of Device 3 discussed in Fig. 4. **a** Optical micrograph of the circuitry. Top left inset shows a detection switch. **b** Optical micrograph of the cavity with edge contacts to the graphite (top right contact) and to the graphene (all other contacts).

Having demonstrated the role of hybridization in a device where resonances were well separated in frequency, we now turn to the observation of ultrastrong coupling between graphene and graphite plasmonic cavity modes within the experimental spectral sensitivity range. To enter this regime, we designed the heterostructure (Extended Data Table 1 and Extended Data Fig. 4), using our analytical theory, such that four modes, the graphite cavity mode centred at 0.43 THz in addition to three graphene modes, *s* = 0, 1, 2, overlap in frequency within the experimental measurement range of the terahertz circuit. This was achieved by making both the graphite and the hBN between graphene and graphite thinner (Fig. 4a) as compared with the device in Fig. 3.

The ultrastrong coupling of graphene and graphite plasmonic self-cavity modes is indicated by the near-field carrier-density-dependent conductivity spectra, shown in Fig. 4b. We fit the spectra with a sum of four Lorentzians to capture the expected modes of the cavity (Supplementary Fig. 6). As the carrier density increases, all modes shift to higher frequencies. At a graphene carrier density of *n* = 0.5 × 10^12^ cm^−2^, the *s* = 2 mode becomes resonant with the *m* = 0 mode (Fig. 4b, dashed line). Rather than crossing in frequency, as would be expected given the different momenta of the *m* = 0 and *s* = 2 modes (Supplementary Figs. 13, 26 and 27), they hybridize and split. The spectral separation of these two modes at this carrier density corresponds to twice the coupling strength *g*, found to be (50 ± 5) GHz, corresponding to approximately 0.2 meV. Comparing this with the frequency of (0.43 ± 0.01) THz, which is approximately 1.8 meV, at which this coupling occurs, we obtained a normalized coupling strength of ${\eta }_{\exp }=0.12\pm 0.01$ characterizing this cavity as in the ultrastrong coupling regime (Supplementary Fig. 22).

The ultrastrong coupling between the graphene and graphite plasmonic self-cavity modes can be seen to modify the electrodynamic properties of the system. Tracking the resonance frequencies of the four modes as a function of carrier density (Fig. 4c) reveals that the lowest-order graphene mode *s* = 0 is again uncoupled from the cavity as it scales with *ν*_*s*=0_ ∝ *n*^0.24±0.01^. However, the *s* = 1 and *s* = 2 modes are suppressed when compared with their uncoupled nature (Supplementary Fig. 22).

The spectral weight of each mode was calculated as the area of each Lorentzian shown in Fig. 4b and plotted as a function of carrier density in Fig. 4d. As the graphene becomes metallic, the spectral weight of the upper branch (purple) decreases, as the lower branch (magenta) increases in spectral weight. This stands in contrast to the expected spectral weight behaviour of graphene and graphite cavity modes in the absence of coupling, where the spectral weight of graphene modes should increase in amplitude and the cavity mode should only be weakly affected when the graphene carrier density *n* is increased (Supplementary Fig. 29). The large transfer in spectral weight across the avoided crossing is thus a consequence of the cavity coupling achieved.

The analytical model captures the carrier-density dependence and spectral weight trends of this device, as shown in the middle panel of Fig. 4c. The theory predicts *η*_theory_ = 0.11 ± 0.05 (Fig. 4c, right), consistent with the experimental observation. The theory further shows that the broadening of the linewidths of the graphene plasmonic modes in device 3 compared with the narrow linewidths detected in device 2 can be attributed to hybridization of the currents in the graphene and graphite layers (Supplementary Fig. 19). The unexpected carrier-density scaling of the plasmon frequency, linewidth broadening and spectral weight transfer can thus be attributed to tunable, multimodal cavity coupling between the plasmonic modes of the graphite and graphene layers, which reach the ultrastrong coupling regime for the *s* = 2 mode (Supplementary Fig. 25).

## Tunable cavity coupling mechanism

**Fig. 5:**
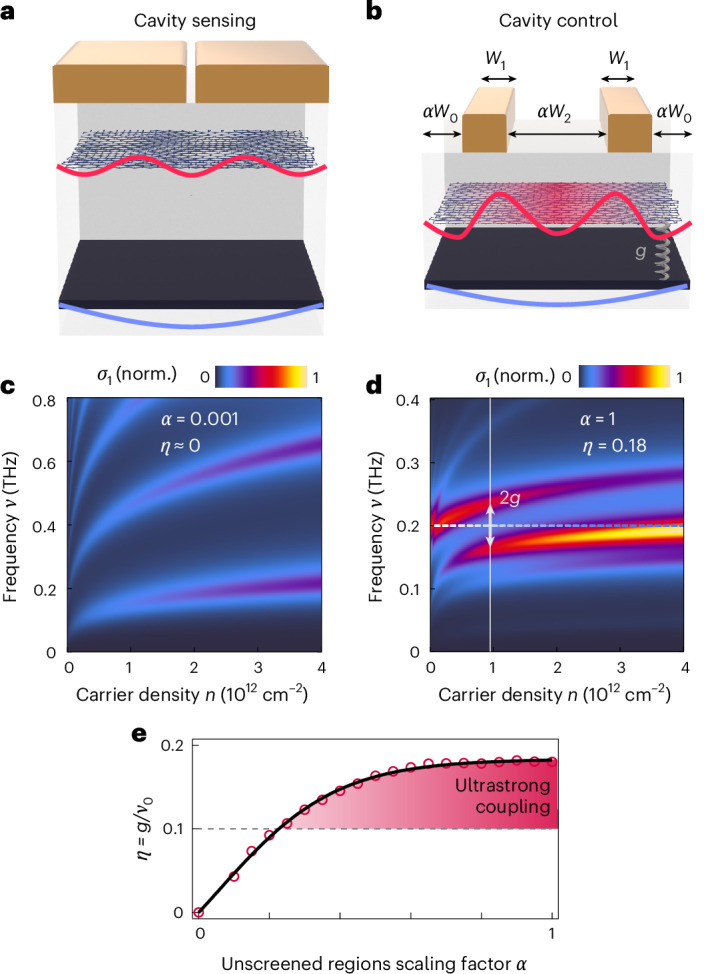
Coupling strength tunability through geometry: from cavity sensing to cavity control. **a**, A cavity with a small-gap transmission line and no extended sample regions behaves as a sensing tool, meaning the properties of a sample of interest can be probed without the additional influence of resonant cavity coupling to the graphite gate. Cavity parameters: *W*_0_ = 0 μm, *W*_1_ = 3 μm and *W*_2_ = 0.01 μm, *d*_gr_ = 9 nm, *d*_hBN1_ = 9 nm and *d*_hBN2_ = 93 nm. **b**, A cavity with extended 2D material region *W*_0_ and gap *W*_2_ can be used to control collective modes of 2D materials. *α* represents a tuning factor for the cavity parameters. Cavity parameters: *W*_0_ = 3 μm, *W*_1_ = 3 μm and *W*_2_ = 8 μm, *d*_gr_ = 9 nm, *d*_hBN1_ = 9 nm and *d*_hBN2_ = 9 nm. **c**, Simulation of a device as in **a** with a graphene monolayer. Multiple undisturbed graphene plasmonic modes can be obtained that all scale as *ν* ∝ *n*^0.25^. **d**, Simulation of a device as in **b** with a graphene monolayer and *α* = 1. An avoided crossing with *η* = 0.18 is observed. **e**, *η* dependence on the scaling factor *α* for the geometry defined in **b**, with cavity sensing in the small *α* limit and cavity control in the large *α* limit. Source data

While the devices highlighted in this work exhibited coupling between sample and graphite cavity modes, it would be useful to also be able to build cavities with negligible coupling such that the unperturbed electrodynamics of the sample can be interrogated. The analytical model developed as part of this work provides design principles to construct cavities which either minimize or maximize coupling strengths to either sense or control the terahertz electrodynamics of collective modes in microstructured quantum materials (Fig. 5).

The theory predicts that a heterostructure built with a thick hBN, thick graphite gate and with a transmission line geometry patterned to cover all but a small gap between the traces (*αW*_2_ ≪ *W*_1_; Fig. 5a) minimizes the coupling between the graphene and graphite self-cavity modes (Fig. 5c). In such a device, momentum conservation is preserved and, thus, the graphite and 2D material plasmon modes are orthogonal to each other. Our theory and numerical simulations show that both the current density *j* and the spatial derivative, *dj*/*dx*, are approximately continuous throughout the device (Supplementary Fig. 23). As the terahertz spectroscopic platform introduced here is capable of independently capturing the complex uncoupled 2D material conductivity, which are necessary for extracting correlation-induced spectral weight shifts, measurements of a sensing cavity will provide insight into the dynamics of quantum materials^52^. For example, properties such as quasiparticle scattering rates, eigenfrequencies of collective modes, magnitudes of electronic energy gaps, and superfluid density can be extracted from the sensing cavity conductivity as a function of temperature, displacement and magnetic field, and carrier density.

By contrast, the ultrastrong coupling can be enhanced by patterning the dielectric environment (Fig. 5b). A plasmonic cavity built with increased conductor separation and additional vdW heterostructure widths outside of the transmission line maximizes the coupling between modes of different momenta (Supplementary Fig. 24), with predicted strengths of *η* > 0.18 (Fig. 5b,d,e). This is due to broken translational symmetry of this cavity design and, thus, broken momentum conservation. While discrete plasmonic cavity modes of 2D material and graphite gate are typically orthogonal to each other, the inhomogeneous dielectric environment allows the two cavity modes to spatially overlap and hybridize. Unscreened graphene plasmons are excited in the gap and mediate the coupling of the screened graphite and graphene plasmons. The relative widths of the metal strips to the widths of the unscreened regions provide a tuning knob for the coupling strength. Thus, the coupling can be controlled through the cavity geometry and carrier density, allowing for either probing (*α* ≪ 1) or controlling (*α* ≈ 1) quantum materials (Fig. 5e and Supplementary Fig. 29). The details of this coupling are captured by the analytical theory (Supplementary Figs. 26–29).

## Cavity control of vdW quantum materials

Our results show that terahertz cavity electrodynamics must be taken into account for vdW heterostructures of typical dimensions. As microstructured electrostatic gates and contacts in common vdW device architectures may break momentum conservation in a similar way to the metallic transmission lines used in this work, cavity-coupling effects in vdW heterostructures may even be relevant for understanding the emergence of quantum phases.

The platform developed here delivers a route to deterministic cavity control of vdW heterostructures. These advances in on-chip THz circuitry allow both the cavity coupling strength and spectral weight transfer to be probed under the same experimental conditions in which d.c. transport is measured. With this, the effects of cavity-mediated coupling on the ground state, as probed by transport, could be experimentally related to the cavity hybridization, as probed by terahertz measurements. Moreover, the subwavelength nature and broken symmetries in these microstructured self-cavities could enable hybridizing and probing a variety of collective modes. With this strategy, these devices provide a platform for both spectroscopic characterization and cavity-based control of emergent properties in vdW heterostructures.

## Methods

### Device fabrication

Graphene monolayers, few-nanometre-thick graphite and hBN crystals (15–100 nm) were obtained by mechanical exfoliation onto silicon substrates with a 285-nm SiO_2_ layer. Thicknesses, morphology and cleanliness of the exfoliated 2D crystals were characterized with an atomic force microscope. Atomic force microscope anodic oxidation lithography was used to cut graphene and graphite flakes into well-defined shapes^53^. Subsequently, the heterostructures were fabricated in ambient conditions by the dry-transfer technique using propylene carbonate stamps. The fully assembled heterostructures were transferred to a sapphire substrate (1 × 1 cm^2^, 2 mm thick, c-cut, two-side epi-polished), which has a nearly constant dielectric function (*ϵ* ≈ 10) over the relevant gigahertz-to-terahertz frequency band. Optical lithography (LOR-7B and ma-P 1205) and reactive ion etching (O_2_ and SF_6_) were used to precisely define the shape of the cavity and create clean sample edges. After the cavity was shaped, edge contacts (7 nm Cr and 40–100 nm Au) were patterned by optical lithography and e-beam evaporation. Finally, the optoelectronic circuitry was fabricated in two steps. First, three silicon (α-Si) patches were evaporated to form the terahertz antenna and photoconductive detector switches. Second, the metallic leads to the terahertz antenna and the two coplanar strip transmission lines for the reference and cavity side were fabricated. The transmission lines have a conductor–gap–conductor geometry of approximately 3–3–3 μm and consist of a titanium (10 nm) and gold (275 nm) layer.

### Cavity dimensions

The geometric dimensions of the bare graphite cavity discussed in Fig. 2 (device 1), the graphene cavity discussed in Fig. 3 (device 2) and the ultrastrongly coupled graphene cavity discussed in Fig. 4 (device 3) were obtained by optical and atomic force microscopy and are defined in Extended Data Fig. 1 and listed in Extended Data Table 1. The error bars for the flake thickness measurements are approximately 0.5 nm, and for the transmission line and cavity width measurements approximately 10 nm. The widths *W*_0_ of the cavity region extending the transmission line (Extended Data Fig. 1) vary slightly along the length axis of the cavity and are slightly asymmetric between the left and right sides. The values for *W*_0_ given in Extended Data Table 1 are averaged values that are used for the analytical theory simulations. The full three-dimensional electromagnetic simulations discussed below use actual flake shapes, including varying *W*_0_ and *l*, obtained from the optical micrographs shown in Extended Data Figs. 2, 3 and 4.

The geometric parameters used in the analytical theory simulations of the sensing cavity (Fig. 5c) and the ultrastrongly coupled cavity (Fig. 5d) are given in Extended Data Table 1.

### On-chip terahertz spectroscopy measurements

The measurement set-up is built around a Yb-based femtosecond laser (Spirit HE 1040-16) which provides pulses at a central wavelength of 1,040 nm and full width at half maximum of 310 fs at a repetition rate of 200 kHz. For more efficient operation of the photoconductive switches, the 1,040-nm pulses were frequency-doubled in a thin beta barium borate crystal to generate green 520-nm light. This green light was split into two paths, one for terahertz generation and the other for terahertz detection. The beam for terahertz generation was intensity modulated by an acousto-optic modulator that sent every second pulse onto the chip. The beam for detection was routed through two different optical delay lines: a translation stage and a fast optical delay line (scanDelay from APE). The latter was used for terahertz measurements to generate a time delay of up to 50 ps between generation and detection pulses at a scanning frequency of 2 Hz. After the delay lines, the detection beam was split into two for detecting the cavity and reference side of the optoelectronic circuitry. All three beams were coupled in a microscope assembly and focused onto the different photoconductive switches. The microscope provided an optical image of the device and was used for active alignment stabilization.

The sample was mounted in a continuous-flow optical cryostat (ST500 from Janis), and all measurements were performed at a base temperature of 7–20 K. Currents generated by the photoconductive detector switches were collected by home-built transimpedance amplifiers (2 × 10^8^ V A^−1^, 100 kHz bandwidth) and the generated voltage signal was demodulated by a lock-in amplifier at the frequency of the intensity modulation of the terahertz generation (100 kHz). The lock-in output was digitized by a data acquisition card and synchronized to the fast optical delay line (2 Hz) to recover the terahertz signal.

## Online content

Any methods, additional references, Nature Portfolio reporting summaries, source data, extended data, supplementary information, acknowledgements, peer review information; details of author contributions and competing interests; and statements of data and code availability are available at 10.1038/s41567-025-03064-8.

## Supplementary information

Supplementary Information Supplementary Sections 0.1–0.10 and Figs. 1–30.

## Source data

Source Data Fig. 2 Statistical source data.

Source Data Fig. 3 Statistical source data.

Source Data Fig. 4 Statistical source data.

Source Data Fig. 5 Statistical source data.

## Figures and Tables

**Extended Data Table 1:**
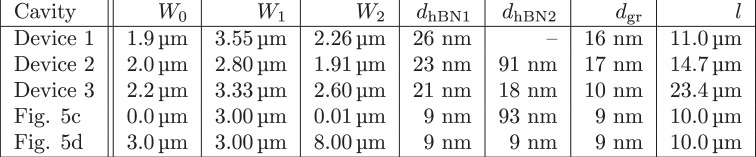
Cavity geometry dimensions

Table containing further details on the geometry of each experimental device discussed in this study.

## Data Availability

The source data for all main and extended data figures are included at the end of this Article. In addition, raw and source data that support the findings of this Article are available at 10.17617/3.YUZ9O9 (ref. ^54^). Materials are purchased from standard suppliers unless otherwise noted (hBN), and analysis code is described in the Supplementary Information. Source data are provided with this paper.
