# Supplementary material for: Cavity electrodynamics of van der Waals heterostructures
Source: Nat Phys. 2025 Oct 20;21(12):1926–33. doi: 10.1038/s41567-025-03064-8 (PMC12695651; doi:10.1038/s41567-025-03064-8)
Supplement: Supplementary file 1 — Supplementary Sections 0.1–0.10 and Figs. 1–30. [file 41567_2025_3064_MOESM1_ESM.pdf]

nature physics

Supplementary information https://doi.org/10.1038/s41567-025-03064-8

Cavity electrodynamics of van der Waals heterostructures

In the format provided by the authors and unedited

Contents

S0.1 Extracting the THz conductivity . . . 3
S0.1.1 Data analysis for Device 1 (Fig. 2) . . . 9
S0.1.2 Data analysis for Device 2 (Figure 3) . . . 11
S0.1.3 Data analysis for Device 3 (Figure 4) . . . 14
S0.1.4 Experimental data from additional gate-tunable graphene cavity 17
S0.2 Full 3D electromagnetic simulations . . . 18
S0.2.1 Method . . . 18
S0.2.2 Simulating bare graphite cavities . . . 20
S0.3 Analytical model for vdW heterostructure cavity electrodynamics . . . 23
S0.3.1 Theoretical description of a bare graphite cavity . . . 23
S0.3.2 Theoretical description of a heterostructure with two self-cavities: 2D material and graphite gate . . . 30
S0.3.3 Influence of hBN anisotropy . . . 36
S0.4 Dependence of the cavity length on the cavity conductivity per micron 37
S0.5 Plasmonic cavity response vs Drude response . . . 38
S0.6 Influence of hybridisation on linewidths . . . 42
S0.7 Comparing experimental data, full 3D electromagnetic simulations and analytical model . . . 44
S0.7.1 Device 1 . . . 44
S0.7.2 Device 2 . . . 45
S0.7.3 Device 3 . . . 47
S0.7.4 Sensing cavity simulations for Fig. 5 . . . 50
S0.7.5 Ultrastrongly coupled cavity of Fig. 5 . . . 52
S0.8 Multimodal coupling . . . 53
S0.9 Coupling mechanism . . . 55
S0.10Quantum fluctuations in the ultrastrong coupling regime . . . 61

In these supplementary notes, we provide additional details of the data analysis procedure, the analytical model developed for this work and supporting full 3D electromagnetic simulations. In Sec. S0.1, we report on the analysis protocol and how the cavity conductivity is extracted from measured time-domain THz transients for Devices 1, 2, and 3. In Sec. S0.2, we present details and the methods of the full 3D electromagnetic simulations that significantly supported the development of the analytical model. The analytical model is summarized both for the case of a bare cavity as well as for a heterostructure with two self-cavities (consisting of a 2D material and a graphite gate) in Sec. S0.3. In Sec.S0.4 we discuss the dependence of the cavity conductivity on the length of samples, and in Sec.S0.5, we expand on the ubiquity of self-cavity effects expected for measurements of vdW heterostructures with a wide range of conductivities. Sec.S0.6 illustrates the impact of hybridization on the linewidth of measured modes. In Sec. S0.7, we compare experimental data, full 3D electromagnetic simulations, and results from the analytical model to show that all are in good agreement, supporting the interpretation of the experimental data and validating the developed analytical model. Given the multimodal nature of the hybridization observed in Device 2 and 3, Sec.S0.8 illustrates the hybridization between a cavity mode and multiple material resonances. In Sec. S0.9, we discuss the coupling mechanism and how the coupling strength can be controlled through geometry. Finally, Sec.S0.10 simulates the modified quantum fluctuations that occur in the ultrastrong coupling regime.

Duration and volume of study

This project started in January 2020 and ended in March 2024. During this time, vdW heterostructure microcavity fabrication protocols were developed, the ultrafast cryogenic measurement setup was built, THz circuitry for probing the cavity conductivity was developed and optimized, an analysis framework for extracting the cavity conductivity was constructed, finite element simulations to simulate the cavity modes were performed, and the analytical theory describing the cavity modes and their coupling was developed. For this report, 42 THz devices were measured in total: 15 THz circuitry optimization devices, 16 bare graphite cavities, 3 graphene devices without graphite cavity, 2 cavities based on transition metal dichalcogenide (TMD) gates, and 6 gate-tunable graphene cavity devices.

S0.1 Extracting the THz conductivity

The goal of on-chip time-domain THz spectroscopy is to extract the complex, frequency-dependent conductivity of a sample of interest in the GHz-THz spectral range. In this section, the models used to extract the optical conductivity of the devices are described, derived and discussed.

Importantly, the setup of on-chip THz spectroscopy is very comparable to a scenario in optics, where an electromagnetic wave propagates from a medium with a refractive index n_0 to a medium of length l with a refractive index n_1, before it continues propagating in a medium with n_0 (see Figure S1). At the interfaces between the different media, the wave is partially reflected and partially transmitted. The transmission and reflection coefficients at the two interfaces can be obtained by using Fresnel's equations at normal incidence.

The reflection and transmission coefficients at the interface from medium with n_0 to a medium with n_1 are therefore [2]:

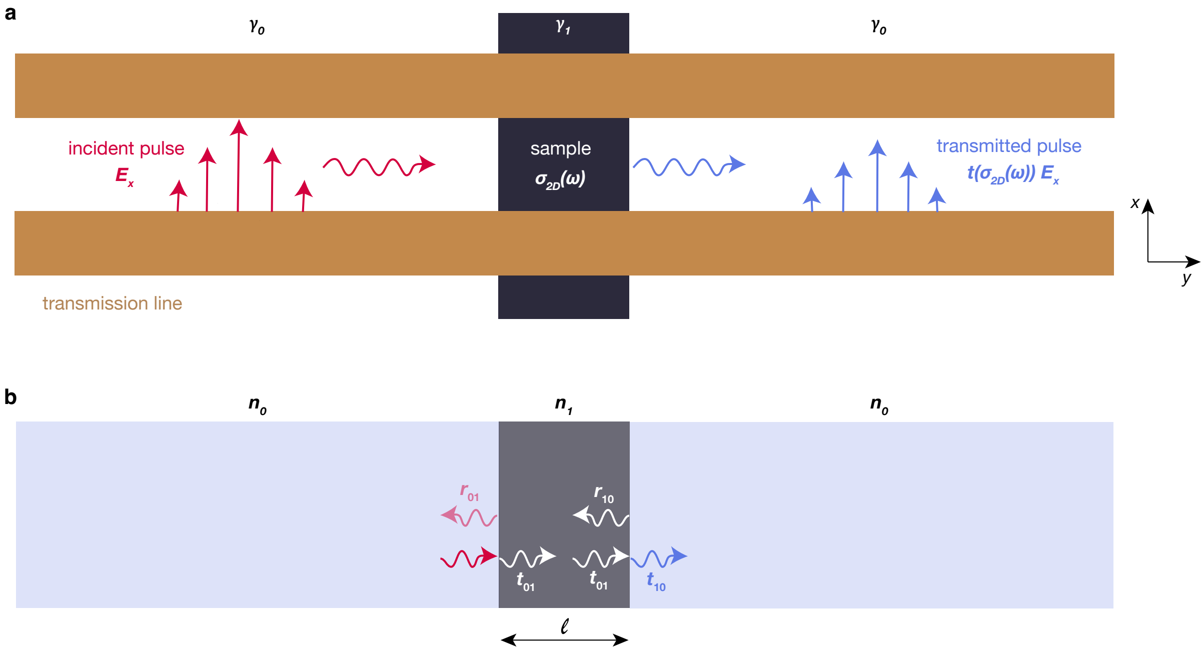

Fig. S1 Optics ansatz for on-chip THz spectroscopy a: An incident THz pulse E_x propagates in a transmission line with propagation constant γ_0. When it interacts with the sample with 2D conductivity $\sigma_{2\mathrm{D}}\omega$, the propagation constant is γ_1. The transmitted pulse $t(\sigma_{2\mathrm{D}}\omega)E_x$ continues propagating with a propagation constant γ_0. **b** The scenario can be compared to an optics picture, where waves propagate in a dielectric with refractive index n_0 before interacting with the sample (refractive index n_1), where the wave gets reflected at both interfaces. The sample length l defines how much of the wave is reflected. Adapted from [1].

$$t_{01} = \frac{2n_0}{n_0 + n_1}, \tag{1}$$

$$r_{01} = \frac{n_0 - n_1}{n_0 + n_1}. \tag{2}$$

For the second interface between the sample medium with n_1 and the medium with n_0, the coefficients are similarly:

$$t_{10} = \frac{2n_1}{n_0 + n_1}, \tag{3}$$

$$r_{10} = \frac{n_1 - n_0}{n_0 + n_1}. \tag{4}$$

Next, a phase shift Φ has to be considered that the wave experiences when propagating in the medium with n_1 over a length of l (sample side) when compared to a wave that continuously propagates in a medium with n_0 (reference side) [3]:

$$\Phi(\omega, l) = e^{\frac{i\omega l}{c} n}. \tag{5}$$

Thus, the transmitted electromagnetic wave $E_t(\omega)$ that propagated across both interfaces on the sample side can be described by:

$$E_t(\omega) = E_0(\omega) t_{01} t_{10} e^{\frac{i\omega l}{c} n_1}. \tag{6}$$

However, another effect that also has to be considered is that the wave can reflect several times within the sample (medium with n_1), as in a Fabry-Pérot interferometer. The internal resonance enhancement factor of a Fabry-Pérot resonator is defined as [4]:

$$F = \frac{1}{1 + r_{01} r_{10} e^{2i \frac{\omega l}{c} n_1}}. \tag{7}$$

The overall transmitted electromagnetic wave is therefore on the sample side:

$$E_{\mathrm{sam}}(\omega) = E_0(\omega) \frac{t_{01} t_{10} e^{\frac{i\omega l}{c} n_1}}{1 + r_{01} r_{10} e^{2i \frac{\omega l}{c} n_1}}, \tag{8}$$

and on the reference side:

$$E_{\mathrm{ref}}(\omega) = E_0(\omega) e^{\frac{i\omega l}{c} n_0}. \tag{9}$$

Now, it is helpful to translate the derived equations in the optics picture to the language that is typically used to describe circuits, namely Telegrapher's equations, that model the change in the voltage and current of a wave propagating down the

transmission lines [5]. The circuit can thereby be treated as being comprised of elements of infinitesimal segments, dy, described by a resistance (R), inductance (L), shunt capacitance (C), and conductance (G), all per unit length. The solution to these equations can be found as a superposition of traveling, attenuated waves propagating to the left and right along y, of the form,

$$\tilde{V}(y) = \tilde{V}^{+}(y)e^{-\gamma y} + \tilde{V}^{-}(y)e^{\gamma y} \tag{10}$$

$$\tilde{I}(y) = \tilde{I}^{+}(y)e^{-\gamma y} + \tilde{I}^{-}(y)e^{\gamma y}, \tag{11}$$

where $\gamma = \sqrt{(R + i\omega L)(G + i\omega C)}$ is defined as the propagation constant of the transmission lines, and ω corresponds to the angular frequency $\omega = 2\pi\nu$. In a dissipationless transmission line, which is a valid approximation for the empty coplanar strip transmission lines used in this experiment, the propagation constant is given by $\gamma_0 = i\omega\sqrt{LC}$. Note that we assume perfect mode matching that is captured by conservation of current and voltage across the interface. In reality there are radiative corrections to these assumptions that we assume to be negligible.

The goal is to transform the above derived equations into frequency dependent voltages $V_{\text{sam}}(\omega)$ and $V_{\text{ref}}(\omega)$, that are functions of propagation constants γ and that can be measured with on-chip THz spectroscopy, to then calculate the optical near-field conductivity based on the fraction of $V_{\text{sam}}(\omega)$ and $V_{\text{ref}}(\omega)$. To isolate the propagation constant of the sample from that of the transmission lines, a measurement scheme and analysis framework analogous to that of free-space THz can be derived [1, 6, 7].

When a sample is placed beneath the coplanar strip transmission lines such as in the geometry of the experiment, it modifies the complex propagation constant, denoted by γ_{sam}.

The propagation constant γ (Telegrapher's equations) and refractive index n (optics) are then related by

$$\gamma = ik = \frac{i\omega n}{c}. \tag{12}$$

$V_{\mathrm{sam}}(\omega)$ and $V_{\mathrm{ref}}(\omega)$ can therefore be written as:

$$V_{\mathrm{sam}}(\omega) = V_0(\omega)\frac{t_{01}t_{10}e^{\gamma_{\mathrm{sam}}l}}{1 + r_{01}r_{10}e^{2\gamma_1 l}}, \tag{13}$$

$$V_{\mathrm{ref}}(\omega) = V_0(\omega)e^{\gamma_0 l}. \tag{14}$$

By using Fresnel's equations, Equations 1, 2, 3, 4, and replacing the refractive indexes by propagation constants, the fraction of the measured $V_{\mathrm{sam}}(\omega)$ and $V_{\mathrm{ref}}(\omega)$ can be calculated:

$$\frac{V_{\mathrm{sam}}(\omega)}{V_{\mathrm{ref}}(\omega)} = \frac{\frac{2\gamma_0}{\gamma_0+\gamma_{\mathrm{sam}}}\frac{2\gamma_{\mathrm{sam}}}{\gamma_0+\gamma_{\mathrm{sam}}}e^{(\gamma_{\mathrm{sam}}-\gamma_0)l}}{1 + \frac{\gamma_0-\gamma_{\mathrm{sam}}}{\gamma_0+\gamma_{\mathrm{sam}}}\frac{\gamma_{\mathrm{sam}}-\gamma_0}{\gamma_0+\gamma_{\mathrm{sam}}}e^{2\gamma_{\mathrm{sam}}l}}, \tag{15}$$

which can be further transformed to [1, 6]:

$$\frac{V_{\mathrm{sam}}(\omega)}{V_{\mathrm{ref}}(\omega)} = \frac{2\frac{\gamma_{\mathrm{sam}}}{\gamma_0}e^{-\gamma_0 l}}{(2\frac{\gamma_{\mathrm{sam}}}{\gamma_0})(\cosh(\gamma_{\mathrm{sam}}l)) - (1 + (\frac{\gamma_{\mathrm{sam}}}{\gamma_0})^2)\sinh(\gamma_{\mathrm{sam}}l)}. \tag{16}$$

Here, $\gamma_0(\omega)$ is the propagation constant of the empty transmission lines simulated using full 3D electromagnetic simulations and l is the length of the sample. While this can be analytically simplified using a thin film assumption [1], numerically solving Equation 15 provides more accurate results and was used for all the calculations of conductivities shown throughout this study. Numerical solving was performed using the findroots solver in IgorPro, which uses a Jenkins-Traub algorithm for solving the zeros of a polynomial [8].

As we will see later, the changes in the propagation constant, γ, that occur when a sample is present are due to the excitation of a current in the material between the transmission lines. Thus, the γ_{sam}, calculated above, can be related to the longitudinal

optical conductivity of the cavity $\sigma_{\text{eff}}(\omega)$, which in turn is related to the dielectric constant (ϵ) of a material through:

$$\gamma^2(\omega) = -\frac{\omega^2}{c^2}\epsilon_{\text{eff}}(\omega) = -\frac{\omega^2}{c^2}(\epsilon_{ref} + \frac{i\sigma_{\text{eff}}(\omega)}{\epsilon_0\omega}), \tag{17}$$

where $\epsilon_{ref} = c^2LC$ accounts for the dielectric response of the empty transmission lines.

The effective macroscopic conductivity is derived from the microscopic conductivity that is spatially varying along the z-direction, $j_{\text{3D}}(z,\omega) = \sigma_{\text{3D}}(\omega, z)E_{probe}(z,\omega)$. Expressing the probe field in terms of eigenmodes of the transmission lines, $E_{probe}(z,\omega) = f(z,\omega)E_0$, we can compute the effective conductivity as the overlap of the z-dependent mode function with the z-dependent current,

$$\sigma_{\text{eff}}(\omega) = \int_{-\infty}^{\infty} dz|f(z,\omega)|^2\sigma_{\text{3D}}(\omega, z). \tag{18}$$

In the presence of 2D layers, the conductivity takes the form $\sigma_{\text{3D}}(z,\omega) = \sum_l \delta(z - z_l)\sigma_{2D,l}(\omega)$, where l corresponds to the l_{th} layer at position z_l along the z-direction. As a result, the effective conductivity can be directly mapped to the 2D layer conductivities of a stacked heterostructure through:

$$\sigma_{\text{eff}}(\omega) = \sum_l |f(z_l,\omega)|^2\sigma_{2D,l}(\omega). \tag{19}$$

In the main text, since the layers we consider are on the order of $10 - 100$ nm away from each other, we assume that $|f(z_l,\omega)|^2$ is constant for the different layers, and therefore the result is given by:

$$\sigma_{\text{eff}}(\omega) \approx C_{\text{filling}} \sum_l \sigma_{\text{2D,l}}(\omega), \tag{20}$$

where $C_{\text{filling}} = |f(z_0,\omega)|^2$, is a multiplicative filling factor that corresponds to the normalized amplitude of the stripline eigenmode at the lower edge of the transmission

lines. This factor is a free parameter in our theory analysis and depends on details of the transmission line itself. However, since it is an overall multiplicative constant that is not affected by the carrier density, it allows for detecting changes in the conductivity of the stacked heterostructure as we dope the graphene.

S0.1.1 Data analysis for Device 1 (Fig. 2)

To extract the conductivity shown in Fig. 2, time domain data were measured on both the cavity and reference side of the circuit using the procedure outlined in the Methods section. The amplitudes of the time domain traces were calibrated using the procedure outlined in Ref. [9]. For each photoconductive switch, the voltage output of the transimpedance amplifier is recorded as a function of known voltage bias across the switch. This measurement curve is fit with a linear function to extract the slope. To obtain the electric fields shown in the time-domain traces, the detected voltage is divided by this slope, and by the distance between the transmission lines to determine the electric field:

$$E_{\mathrm{THz}} = \frac{V_{measured}}{m_{calibration}} \cdot \frac{1}{W_2}. \tag{21}$$

A binomial filter was applied with a low-pass filter frequency of 1.5 THz to eliminate high frequency noise. The data traces were windowed to avoid reflections from known sources and the Fourier transforms were taken using standard numerical procedures. The frequency domain data were then used to numerically calculate the complex optical conductivity using Eq. 15, and the derivation outlined in Sec. S0.1. The conductivity shown in Fig. 2 was found to be independent of the windowing procedure. Full 3D electromagnetic simulations of the device were used to extract the ratio of a field transmitted through the sample as compared to an empty circuit. Using the simulations input introduces a systematic uncertainty of $\approx 10\%$ on the amplitude of the

calculated conductivities, but has negligible impact on the lineshape of the resonance (or the carrier-density dependent behaviour of Devices 2 and 3).

The data were fit using a Lorentz oscillator model, ($\sigma(\nu) = \frac{A}{2\pi\cdot\Gamma + i(\nu\cdot 2\pi - \frac{(\nu_0\cdot 2\pi)^2}{\nu\cdot 2\pi})}$), where Γ corresponds to the linewidth, ν_0 the resonance frequency, and A the amplitude. Using these parameters, the cavity quality factor can be extracted for the device. On Device 1, (Fig. 2 of the main text), a linewidth of (0.29 ± 0.01) THz and a resonance frequency of (0.43 ± 0.01) THz were extracted, giving a quality factor Q of 1.48 $\pm$0.01, where the uncertainty comes from the covariance of the fits. We note here that the linewidth can be related to the mean free scattering time (τ) or scattering rate (τ^{-1}) by $\Gamma = \tau^{-1}$ when the inhomogeneous broadening is small.

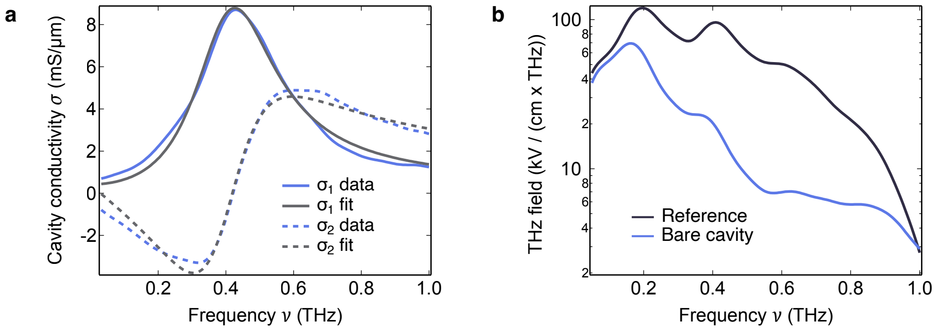

Fig. S2 Fits and Fourier transforms of time-domain data of Device 1: a The data shown in Fig. 2 of the main text were fit with a single Lorentzian to extract the linewidth and quality factor (Q). **b** Real part of the Fourier transforms of the measured transients on the cavity and reference sides shown in Fig. 2b.

S0.1.2 Data analysis for Device 2 (Figure 3)

The conductivities for Device 2 shown in Fig. 3 were calculated at each back gate voltage in a similar way to Device 1. The carrier density was calculated using the relation $n = \frac{\epsilon_0 \epsilon_{\text{hBN}}(V_{\text{g}} - V_{\text{cnp}})}{d_{\text{hBN2}} e}$, where $\epsilon_{\text{hBN}} = 3.7$ [10] is the relative dielectric constant of hBN, ϵ_0 is the permittivity of free space, V_g the voltage applied to the graphite gate, V_{cnp} the voltage at graphene charge neutrality, d_{hBN2} the distance between the graphene and graphite gate (hBN thickness, defined in Table S1), and e the elementary charge. For this device, the charge neutrality point was selected as the back gate voltage that corresponded to the smallest integrated spectral weight in the range of 50 GHz to 1 THz.

The complex conductivity data were fit with a function comprised by a sum of four Lorentzians (as described above, each containing free parameters of amplitude, linewidth, and resonance frequency), corresponding to modes $s = 0, 1, 1', 2$, and a line (with free parameters σ_{offset} and slope). The additional Lorentzian ($s = 1'$) captured a peak that can be seen as a shoulder in Fig. 3 and Fig. S4a, which occurs due to a slight asymmetry of the W_0 regions extending the transmission line (see Extended data Fig. 3). The sloped line captures the conductivity of plasmons, such as the graphite plasmon and higher order graphene plasmonic modes whose resonance frequencies are centered outside of the experimental sensitivity range. The low-frequency tail of these modes contributes to a finite, frequency-dependent offset, and thus the real part of the offset and slope were constrained to be positive. The full fit parameters with error bars are shown in Fig. S4b-h. At low graphene carrier densities, the spectral weight of the graphene plasmons is small and close together in frequency, increasing the errors in these fits.

Extracted parameters such as the quality factor Q were calculated by taking the ratio of the resonance frequency, ν, to the linewidth, Γ. An example using $s = 2$ is

shown in Fig. S4e. The mean free path was calculated as $l_{\mathrm{mfp}} = \frac{v_F}{\Gamma}$ using the linewidth, Γ, and assuming a Fermi velocity of $v_F = 1 \times 10^6\ m/s$ [1].

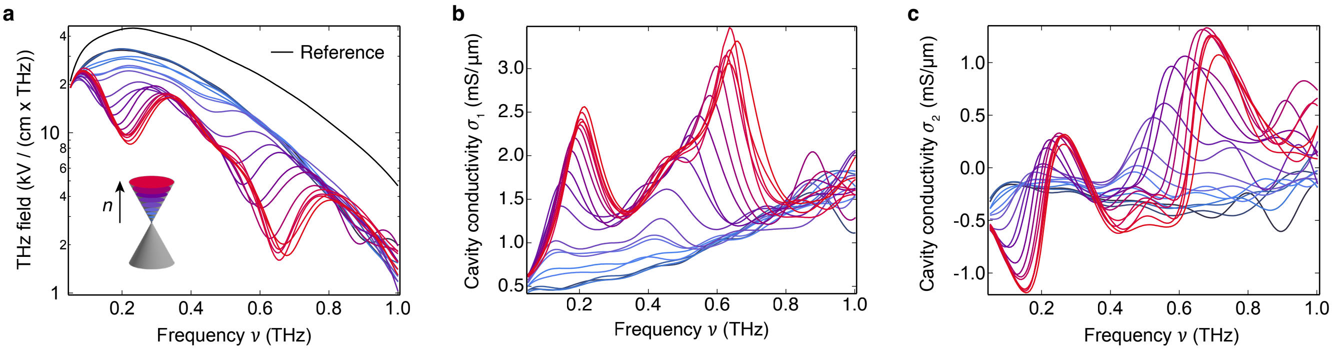

Fig. S3 Additional data for Device 2 discussed in Fig. 3b: a Real part of the Fourier transforms for the reference (black) and for different graphene carrier densities. **b** Real and **c** imaginary parts of the cavity conductivity obtained from the Fourier transforms shown in (a).

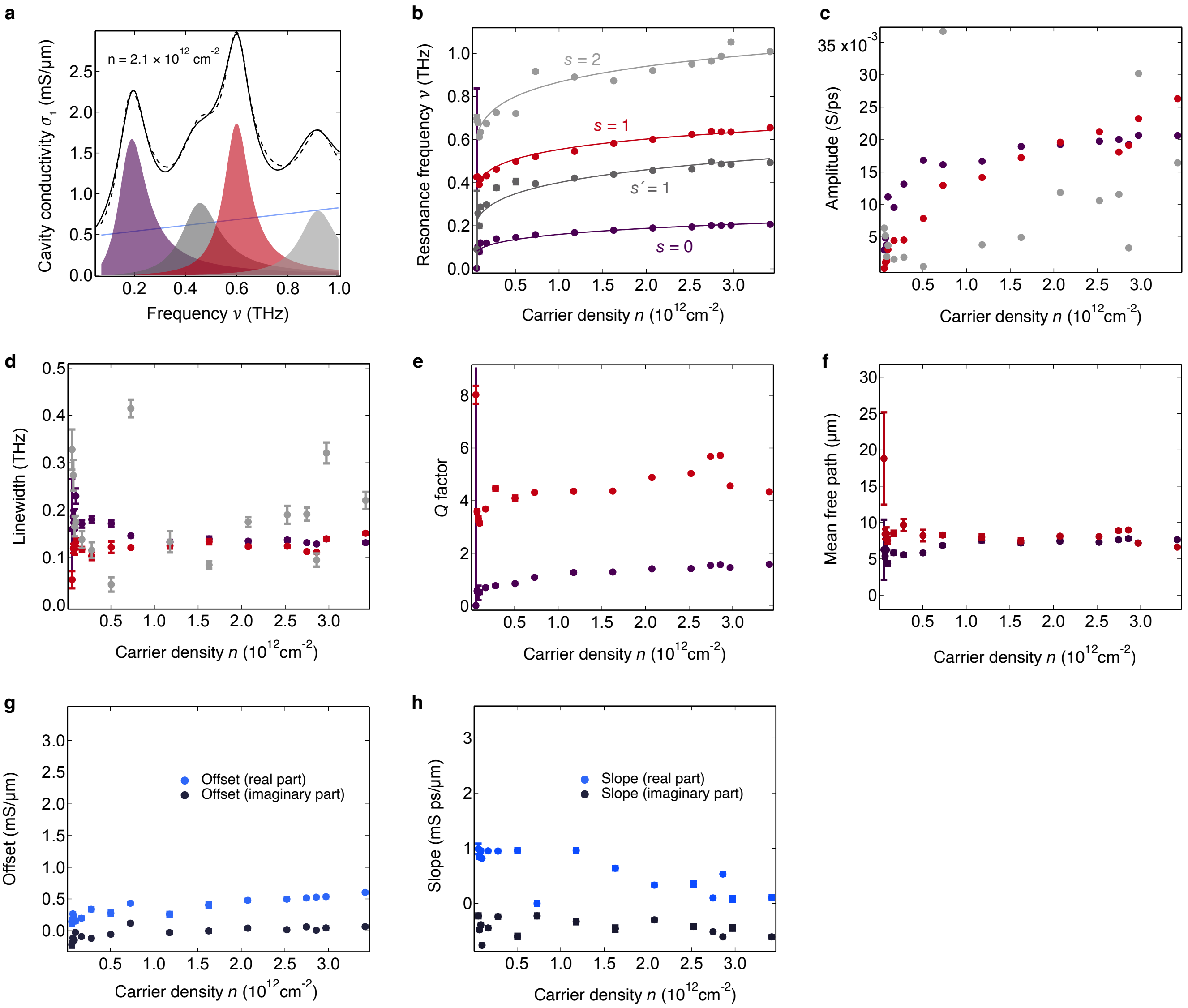

Fig. S4 Fit parameters from the Device 2 shown in Fig. 3: a Exemplary fit result at a graphene carrier density of $n = 2.1 \times 10^{12}$ cm^{-2}. Real and imaginary parts of the cavity conductivity were fit to a sum of four Lorentzians and an offset with a slope. The dark grey mode ($s = 1'$ occurs due to device asymmetry (graphene which extends further on one side than the other). **b** Resonance frequencies, **c** amplitudes, **d** linewidths, **e** quality factors, **f** mean free paths, **g** offsets and **h** slopes as function of graphene carrier density. The uncertainty in each parameter, plotted as error bars, is given by the covariance of the fit.

S0.1.3 Data analysis for Device 3 (Figure 4)

Time-domain traces for Device 3 were measured as a function of back gate voltage, as shown in Fig. S5a. The Fourier transforms were taken (Fig. S5b), and complex cavity conductivity (real part shown in Fig. 4 of the main text) were calculated in the same procedure as Device 1. A reference trace measured on a different circuit of identical design was used for the analysis of Device 3 due to an imperfection in the transmission line on the reference side of Device 3. The back gate voltage corresponding to the charge neutrality point was selected as the back gate voltage which led to the maximum voltage drop across the sample in a 4 point DC resistance measurement.

Due to the congested nature of the spectrum in Device 3, the data were fit using a global fit procedure across all carrier densities. Each carrier density was fit using the same fit function as used for Device 2 (a sum of four Lorentzians and a sloped line, where the resonance frequency of the lowest three frequency modes were assumed to scale with power-law behaviour (the best approximation that could be used to capture the increase in resonance frequency as a function of carrier density). The powers of each of these modes were varied to find the best global fit over all carrier densities.

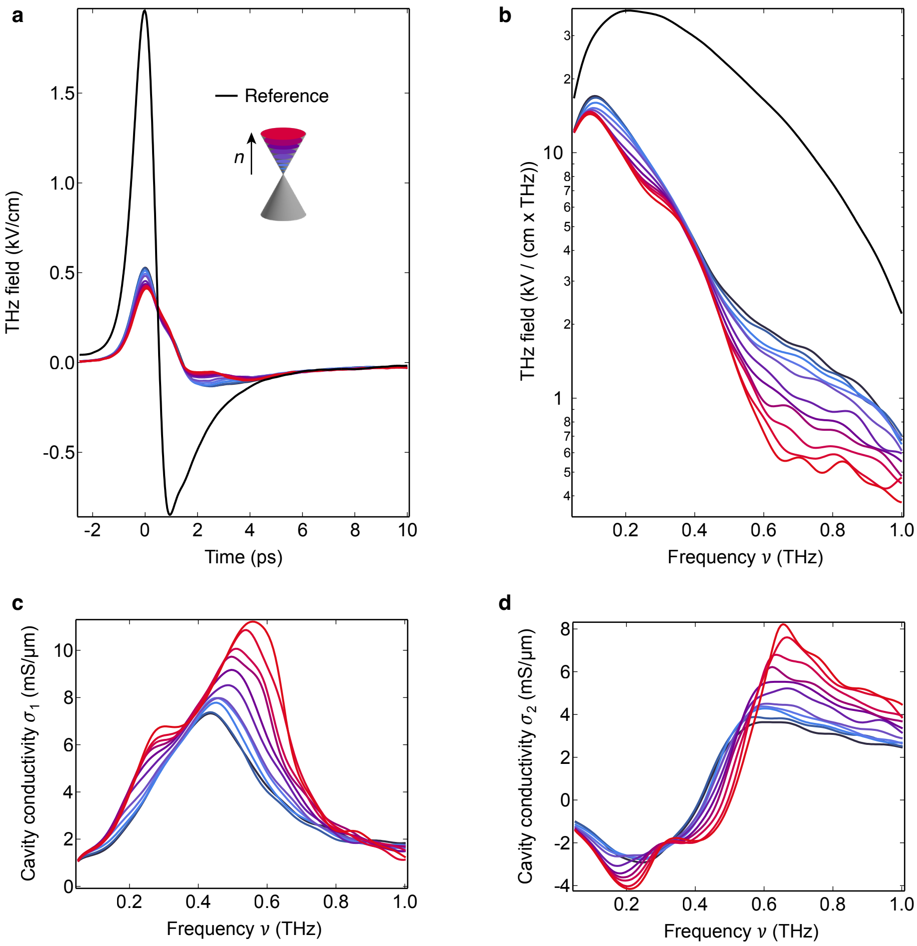

Fig. S5 Additional data for Device 3 discussed in Fig. 4: a Measured time-domain traces taken on the reference transmission line and on the cavity side for different graphene carrier densities. **b** Real part of the Fourier transforms of the transients shown in panel (a). **c** Real and **d** imaginary parts of the cavity conductivity obtained from the Fourier transforms in (b).

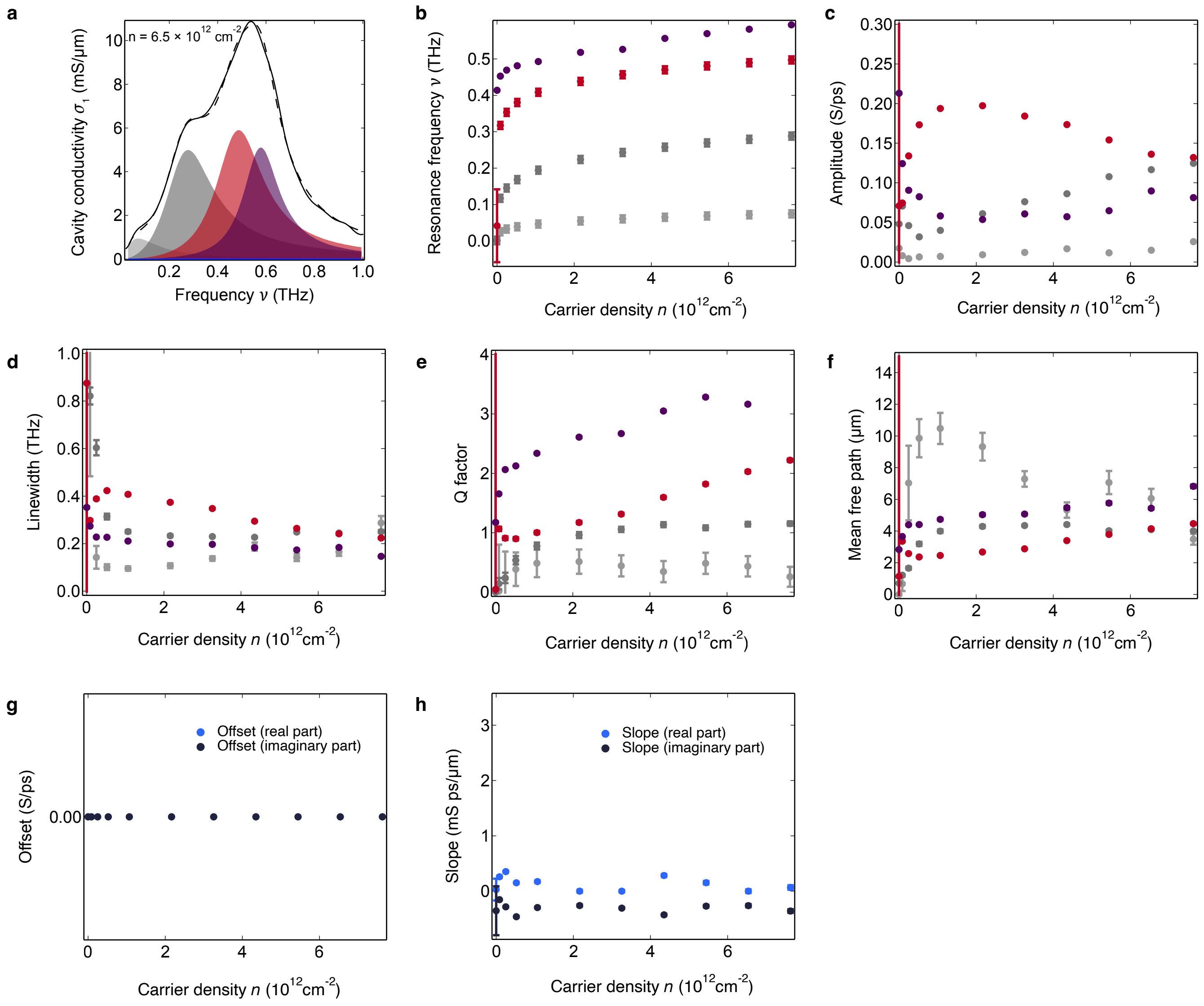

Fig. S6 Fit parameters from Device 3, shown in Fig. 4: a Exemplary fit result at a graphene carrier density of $n = 6.5 \times 10^{12}$ cm^{-2}. Real and imaginary part of the cavity conductivity was fit to a sum of four Lorentzians and an offset with a slope. **b** Resonance frequencies, **c** amplitudes, **d** linewidths, **e** quality factors, **f** mean free paths, **g** offsets and **h** slopes as function of graphene carrier density. The uncertainty in each parameter, plotted as error bars, is given by the covariance of the fit.

S0.1.4 Experimental data from additional gate-tunable graphene cavity

As part of this project, 6 additional gate-tunable graphene devices were measured and characterised, whose results supported the conclusions of this study and were utilised for developing the analytical model. For example, Device 4 had a very similar heterostructure geometry as Device 2 of the main text, and based on our analytic model, is expected to have a cavity resonance ≈ 1.25 THz. Like Device 2, we observed suppressed power law scaling ($\nu \propto n^{\text{pow}}$) of the graphene-like, antisymmetric modes. The purple mode shown in Fig. S7c (corresponding to the resonance at ≈ 0.56 THz for the highest carrier density trace in Fig. S7b) scales with a power of $\approx 0.18 \pm 0.01$. The magenta mode in Fig. S7c (corresponding to the resonance ≈ 0.86 THz in Fig. S7b) scales as 0.14 ± 0.01. These results deviate from the expected power law scaling of $\nu \propto n^{0.25}$, and are interpreted as a signature of coupling to the graphite cavity mode.

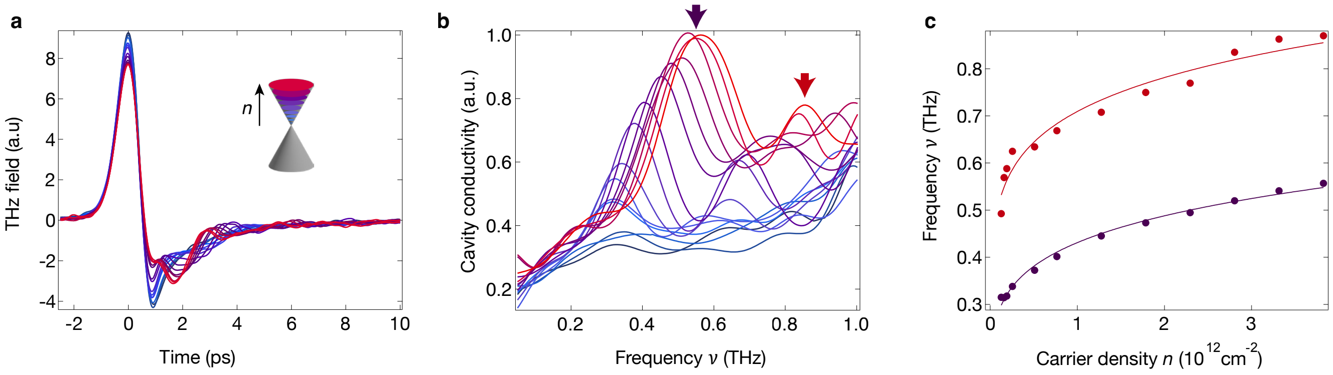

Fig. S7 Experimental data from Device 4: a Measured time-domain traces taken on the cavity side for different carrier densities. **b** Real part of the cavity conductivity for Device 4, exhibiting a sequence of graphene-like resonances. **c** The resonance frequency positions extracted using similar fitting methods as described above for two modes, fit with power-law behaviour.

S0.2 Full 3D electromagnetic simulations

A full 3D electromagnetic solver (CST Studio Suite 2021 from Dassault Systèmes) was utilized in order to understand and verify the frequency dependence of the cavity conductivity, as well as to compute current density profiles in the sub-THz range. The simulations were performed with a frequency domain solver, based on the finite element method (FEM) [11] in the frequency range between 0.3 GHz and 1 THz, in the manner described below.

The frequency domain solver solves Maxwell's equations and calculates scattering parameters (S-parameters) of the ports for a number of sampled frequencies by launching an excitation signal and detecting the signal after it interacted with the sample. An impedance mismatch occurs between empty transmission line and transmission line with sample, which leads to reflections occurring at this interface. By introducing a reference plane for each port at the sample edges, the pure sample contribution was de-embedded.

The S-parameters were converted into an ABCD-matrix (chain matrix A_{ij}) or alternatively into the Z-matrix. The complex conductance Y of the film was extracted from $Y = A_{21}$ or from the respective off-diagonal components of the Z-matrix $Y = 1/2(1/Z_{12} + 1/Z_{21})$. The global cavity conductivity σ in mS/µm was extracted by dividing the obtained complex conductance Y by the device length l.

S0.2.1 Method

The simulation model was defined in a box (typically 140 µm width, 160 µm length and 135 µm height) with open boundary conditions to minimise the reflections of the electromagnetic waves at the walls of the box. The transmission line was oriented along the y-axis (see Extended data Fig. 1) and the sample (graphite flake encapsulated in hBN) was located at the center. Both ends of the transmission lines were terminated by the transmission line ports that are flush with the y-direction open boundary

conditions. We chose a pure odd mode for the launching port signal, which is the dominant mode launched by the THz antenna and coupled into the typical ≈ 3-3-3 µm transmission line geometry (see Fig. 2b and Extended data Fig. 3) [5].

The frequency domain solver requires different material models to simulate the graphite-transmission line microstructures [11]. We chose the following material properties for the individual cavity-materials:

- The sapphire (Al_2O_3) substrate and hBN layer were simulated using the Conventional conductivity model, assuming a complex permittivity of $\epsilon(\omega) = \epsilon' - \epsilon''(\omega)$, with $\epsilon''(\omega) = i\frac{\sigma}{\epsilon_0\omega}$ and $\sigma = 0$ (loss-free). The real part of the permittivity was taken to be $\epsilon' = \epsilon_{sap} = 10$, for sapphire, and $\epsilon' = \epsilon_{hBN} = 3.7$, for hBN. The sign of the real part is always positive and frequency-independent in this model.
- The graphite film was modeled by the Drude dispersion model. This model assumes that the complex permittivity is $\epsilon(\omega) = \epsilon_\infty - \frac{\omega_{3\text{D}}^2}{\omega(\omega - i\omega_c)}$ with $\epsilon_\infty = 21$, the 3D plasma frequency $\nu_{3\text{D}} = \omega_{3\text{D}}/2\pi$ and the scattering rate $\omega_c = 2\pi\nu_c = 2\pi/\tau$, where τ is the mean time between collisions of the carriers. The 3D plasma frequency $\nu_{3\text{D}}$ of graphite in the range of 118 THz - 121 THz (0.49 eV - 0.50 eV) was used for temperatures below 20 K, in accordance with values from the literature [12] and optimized by experimentally measured data sets on graphite devices with varied thicknesses and geometries. Collision frequencies in the range of $\tau^{-1} = 0.08\,\text{THz} - 0.16\,\text{THz}$ were used for the simulations to match the linewidths obtained experimentally.
- Simulations were tested treating the gold strips as perfect electrical conductors with infinite conductivity, and finite-conductivity metals. To simulate a finite conductivity metal, the Lossy metal model was used, which considers the penetration depth of electromagnetic fields inside the metal to be finite. The lossy metal model and perfect electrical conductor gave the same simulation results.

S0.2.2 Simulating bare graphite cavities

An exemplary full 3D electromagnetic simulation of a thin and wide bare graphite cavity, which exhibits five resonances within the spectral sensitivity range, is first discussed to introduce the simulations. In Sec. S0.7, a series of other full 3D electromagnetic simulations are shown in comparison to experimental data and simulations from the analytical theory. The bare graphite cavity discussed here consisted of a 5 nm graphite, a 7 nm hBN, and a transmission line with $W_0 = 0\,\mu\text{m}$ and $W_1 = W_2 = 5\,\mu\text{m}$. The simulation was performed on a cavity assuming a device length $l = 10\,\mu\text{m}$, a graphite 3D plasma frequency of $\nu_{3D} = 118$ THz (0.49 eV) and a scattering rate of $\tau^{-1} = 0.08$ THz.

The simulated bare cavity conductivity spectrum of real and imaginary part (σ_1 and σ_2) is shown in Fig. S8a. The current density profiles (j_x) as function of x for the five resonances that appear in the spectrum are illustrated in Fig. S8b. The profiles show that the current density is continuous across the device, and falls to zero at the edges of the flake, at the outermost corners of the transmission line. Oscillations in

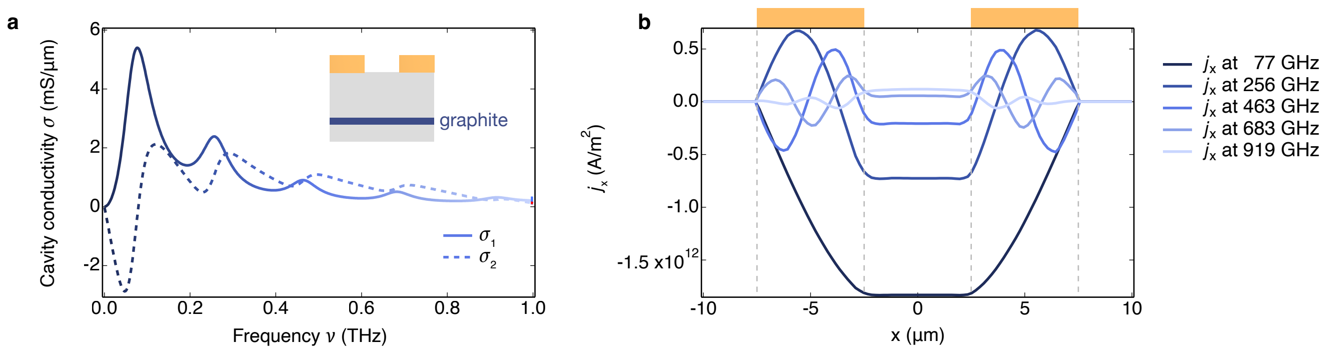

Fig. S8 Full 3D electromagnetic simulation of a bare graphite cavity: a Real and imaginary part of the cavity conductivity as function of frequency obtained by a full 3D electromagnetic simulation of a particularly thin and wide bare graphite cavity, designed to obtain five cavity modes ($m = 0, 1, 2, 3, 4$) within the spectral sensitivity range. Cavity parameters: $W_0 = 0\,\mu\text{m}$, $W_1 = 5\,\mu\text{m}$ and $W_2 = 5\,\mu\text{m}$, $d_{\text{gr}} = 5\,\text{nm}$ and $d_{\text{hBN}} = 7\,\text{nm}$. **b** Current density profiles as function of x for the five resonances obtained in the conductivity spectrum in (a).

the current density occur in the screened regions of the sample, those covered by the metallic transmission lines. In contrast, in the unscreened regions between the metal strips, the current density is primarily flat. When looking at the wavelength of the current density oscillations in the screened regions, one can see that the lowest frequency mode is approximately a quarter wavelength mode, meaning that the wavelength of the plasmonic standing wave in the screened region is about four times as long as the width of the metal strip, W_1. In the limit where conductor separation $W_2 \to 0$, the two screened regions would together admit a half wavelength of standing current, famously resonant in antenna and microwave engineering. The higher frequency modes are then approximately three-quarter, five-quarter, seven-quarter and nine-quarter wavelength modes, defined relative to the individual widths of each coplanar metal strip (W_1). We label these modes $m = 0, 1, 2, 3, 4$, corresponding to the number of current density nodes in the screened regions, below each metal strip. While the cavity simulated here shows five bare cavity modes within the spectral sensitivity range, the cavities discussed in the main paper were intentionally designed to exhibit only one or zero cavity modes below 1 THz.

The electric field distribution in the cavity at the resonance frequency of the $m = 0$ ($= 77\,\mathrm{GHz}$) was simulated for different phases and is shown in Fig. S9. A phase range of $0° - 360°$ defines a full plasmon oscillation. As shown in Fig. S9, most of the electric field is confined in between the two metal strips, pointing in-plane and forming an odd mode. The in-plane orientation of the field in this mode is sensitive to probing the optical conductivity of a 2D sample. It can be seen that this mode excites a current in the graphite, which further leads to modulations of the electric field confined between the gold and graphite, 2D plasmons.

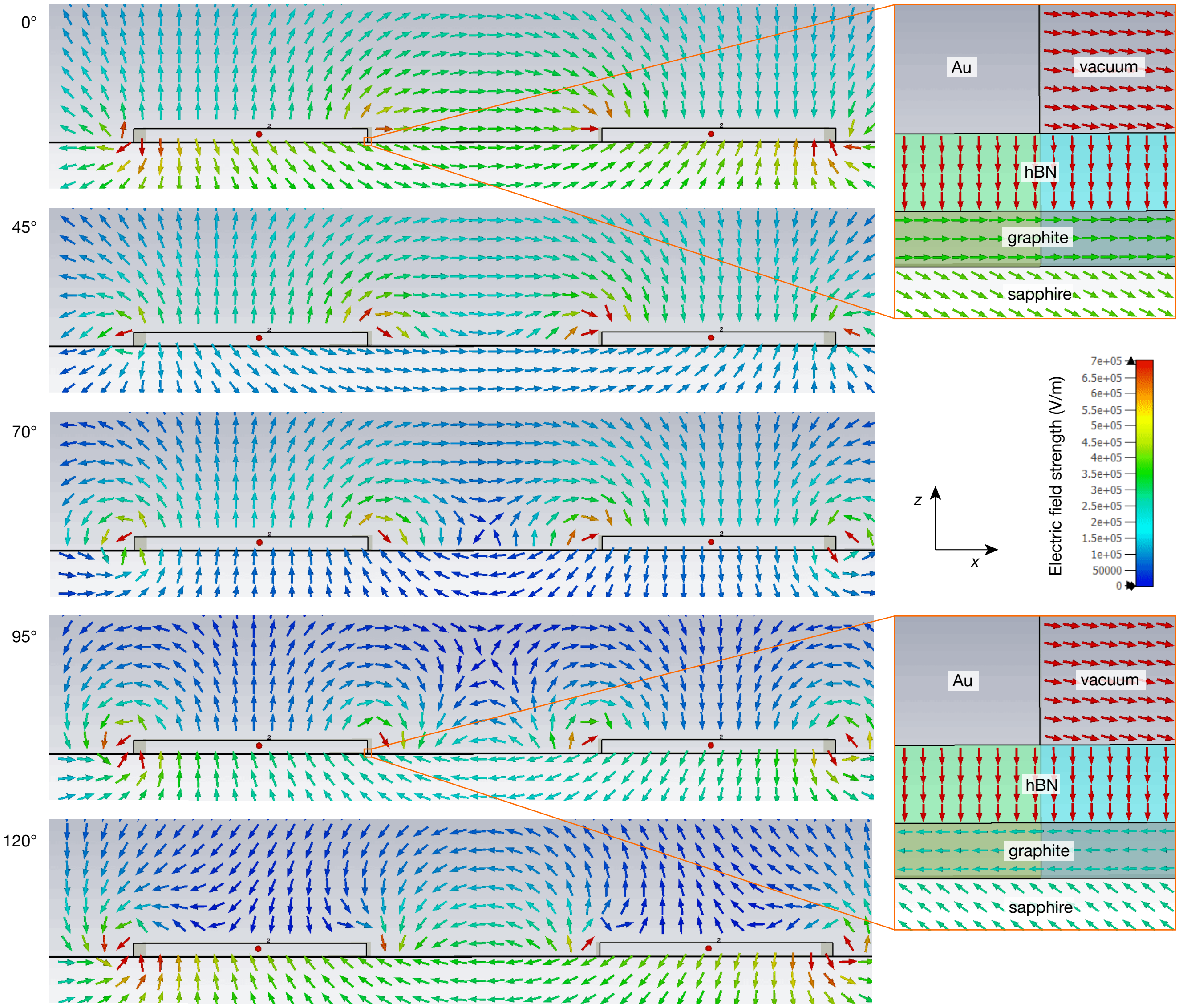

Fig. S9 Electric field distributions for the 77 GHz mode in Fig. S8: Electric field distributions for the 77 GHz mode at different phases ($0° - 120°$). Right panels in orange boxes show zoomed in selections of the electric field distributions in the cavity.

S0.3 Analytical model for vdW heterostructure cavity electrodynamics

In this section, we describe the analytical theoretical framework we developed for on-chip THz spectroscopy of vdW heterostructures, which is shown to give accurate results as compared with the full 3D electromagnetic simulation. In principle, the on-chip THz transmission is a complicated process due to the lack of any spatial symmetries in the system that involves the coupling of many radiative modes as the pulse interacts with the material. This type of coupling is captured by the full 3D electromagnetic simulations. However, our geometries involve 2D plasmonic excitations of typical length of $\sim$ µm in the sub-THz frequency range, which lie in the deep non-relativistic regime away from the light-cone. In this regime, it is possible to describe the effect of the incoming THz pulse on 2D materials in terms of the dynamics of the plasmonic response of 2D materials using Coulomb's law without considering radiative corrections. Similarly, we find that the effect of currents excited in the 2D heterostructure can be captured to a good approximation as an effective conductivity. In Sec. S0.1 we show how the change in effective conductivity determines the transmission coefficient. The good agreement between the analytical model and the full 3D electromagnetic simulations allows the model to be used reliably for the design of future cavities for various different vdW materials, ideally suited for cavity sensing (when the coupling is minimised so the THz spectral response of the undistorted sample is captured), and cavity control (where the THz response captures the effects of the ultrastrong light-matter interaction between the sample and cavity collective modes).

S0.3.1 Theoretical description of a bare graphite cavity

A cross-section of a typical bare graphite cavity is depicted in Fig. S10. The currents along the x-direction can be split up into five regions (0 to IV in Fig. S10), that are either screened (regions I and III) or unscreened (regions 0, II, and IV). To simplify

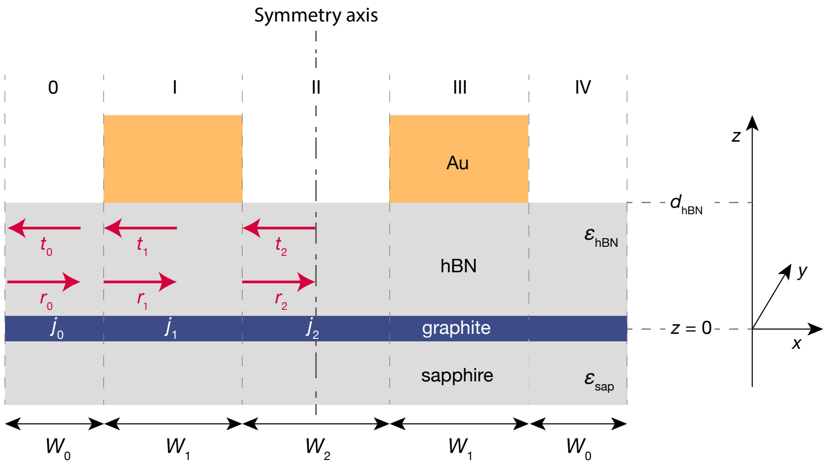

Fig. S10 Cross-section of a bare graphite cavity: The graphite cavity with a top hBN layer is placed on a sapphire substrate and embedded in a transmission line geometry, which provides an inhomogeneous dielectric environment for the 2D plasmon standing waves that form in the graphite layer. The analytical model developed in this work captures the total cavity conductivity by solving Maxwell's equations for transmitted $t_{0,1,2}$ and reflected $r_{0,1,2}$ waves at the boundaries (dashed grey lines) between screened (I and III) and unscreened (0, II and IV) regions.

our calculations, we assume that the cavities are perfectly symmetric with respect to the center of the transmission lines.

2D plasmons at non-zero frequencies have a finite momentum, $q = 2\pi/\lambda$, where λ is the wavelength of the current density wave and the resonance of this oscillation is determined by $\nu_0 = q/\sqrt{L_k C(q)}$, where L_k is the inertial kinetic energy of the cloud of electrons (called kinetic inductance) and C is the capacitance that captures the Coulomb restoring force. In case of an unscreened 2D material, the capacitance is proportional to q and thus the dispersion relation of an unscreened 2D plasmon is $\nu_0 \propto \sqrt{q}$ [13, 14]. Here, the material is considered 2D if the skin depth of electric field is greater than the thickness.

By contrast, when a 2D material with a finite density of states is brought close to a metal (for example in the case of transmission lines in close proximity), image charges form in the metal. The screening and confinement of the electric field are increased, decreasing the resonance frequency for a given momentum. The capacitance becomes q independent as it can then be described by a simple parallel plate capacitor model. The dispersion of a screened 2D plasmon exhibits a linear dispersion ($\nu_0 \propto q/\sqrt{L_K C} \propto q$).

Solving Maxwell's equations gives exact dispersion relations for both screened and unscreened 2D plasmons (see also [13, 15]) as function of 3D plasma frequency of the cavity material (graphite) $\nu_{3\mathrm{D}} \approx 118$ THz - 121 THz, thickness of hBN layer d_{hBN}, thickness of graphite d_{gr}, relative dielectric permittivity of sapphire $\epsilon_{\mathrm{sap}} \approx 10$ and relative dielectric permittivity of hBN $\epsilon_{\mathrm{hBN}} \approx 3.7$ [16]. The dispersion relation of a screened 2D plasmon is:

$$q_{\mathrm{screened}} = \sqrt{\frac{\nu_0^2 \epsilon_{\mathrm{hBN}}}{\nu_{3\mathrm{D}}^2 d_{\mathrm{gr}} d_{\mathrm{hBN}}}}. \tag{22}$$

The dispersion relation of an unscreened 2D plasmon is:

$$q_{\mathrm{unscreened}} = \frac{\nu_0^2 (\epsilon_{\mathrm{hBN}} + \epsilon_{\mathrm{sap}})}{\nu_{3\mathrm{D}}^2 d_{\mathrm{gr}}}. \tag{23}$$

To capture the response of the entire graphite cavity, with broken translational symmetry due to the finite size and different momenta $q_{screened}$ and $q_{unscreened}$, five different, either screened or unscreened, regions have to be considered. This is done by expressing the current in the graphite layer in terms of transmission and reflection coefficients in each region as follows:

$$j_{\text{graphite}}(x,t) = \begin{cases} r_0 E_0 e^{-iq_{\text{unscreened}}(\nu)x} + t_0 E_0 e^{iq_{\text{unscreened}}(\nu)x}, & \text{case } a \\ r_1 E_0 e^{-iq_{\text{screened}}(\nu)x} + t_1 E_0 e^{iq_{\text{screened}}(\nu)x}, & \text{case } b \\ r_2 E_0 e^{-iq_{\text{unscreened}}(\nu)x} + t_2 E_0 e^{iq_{\text{unscreened}}(\nu)x} + \sigma_{2D}(\omega)E_0, & \text{case } c, \end{cases} \tag{24}$$

with case $a : -W_0 - W_1 - W_2/2 < x < -W_1 - W_2/2$, case $b : -W_1 - W_2/2 < x < -W_2/2$ and case $c : -W_2/2 < x < W_2/2$, where due to symmetry, regions III and IV are the symmetry counterparts of 0 and I and omitted in the above expression. Moreover, the symmetry around the middle of the transmission line also leads to $t_2 = r_2$. In addition to hosting the eigenmodes of the graphite layer, region II also supports a current directly excited by the probe pulse, given by $\sigma_{2D}(\omega)E_0$. The coefficients are determined by matching current density j and electrostatic potential V of the plasma modes at the boundaries of each region at $x = -W_1 - W_2/2$ and at $x = -W_2/2$ [17]. Maxwell's equations are used to relate the amplitudes of reflected and transmitted waves of current density to the reflected and transmitted amplitudes of the electrostatic potential V. This is shown schematically in Fig. S10, with the grey dashed lines marking the boundaries. At the edge of the graphite we impose the boundary conditions $j_0(x = -W_0 - W_1 - W_2/2) = 0$.

By then defining the coordinate systems for the cases a (region 0), b (region I) and c (region II) in a way that $x = 0$ at the (left) graphite edge for case a, $x = 0$ at the left metal edge for case b and $x = 0$ at the center of the sample for case c six boundary equations can be obtained in total[1]:

[1] The offset of the different x-axes amounts to a rescaling of the r and t coefficients in the different regions. The RPA conductivity is later defined such that $x = 0$ at the sample center and the equations will be solved for r_2, such that the rescaling of the other r and t coefficients does not contribute to the overall solution.

$$\boxed{\begin{aligned}
&j_{1,\text{graphite}}(W_1,t) = j_{2,\text{graphite}}(-W_2/2,t)\\
&V_{1,\text{graphite}}(W_1,t) = V_{2,\text{graphite}}(-W_2/2,t)\\
&r_2 = t_2\\
&j_{1,\text{graphite}}(0,t) = j_{0,\text{graphite}}(W_0,t)\\
&V_{1,\text{graphite}}(0,t) = V_{0,\text{graphite}}(W_0,t)\\
&j_{0,\text{graphite}}(0,t) = 0,
\end{aligned}} \tag{25}$$

where E_{ext} is the external electric field, that excites the plasmons in the graphite layer in the transmission line gap (region II).

We emphasize that in our approach we make the following distinct approximations. First, radiative corrections to these modes are neglected, and second, refraction at the interface of screened and unscreened heterostructures is assumed to be purely local and captured by boundary conditions on the electrostatic potential and currents. The second approximation is found to be a good approximation for screened systems [17], which we also confirm explicitly in our geometry with the full 3D electromagnetic simulations. The third approximation employed in this modeling is assuming that the dispersion of the 2D polaritons is determined by the homogeneous conductivity i.e. $\sigma(\omega, q) \approx \sigma(\omega, 0)$. This approximation is valid for both graphene and graphite as long as we are probing 2D polaritons with momenta much smaller than the Thomas-Fermi screening wavevector of each layer, where non-local corrections to the conductivity [18–21] are expected to be small. Finally, the fourth approximation we employ is assuming that each layer in a heterostructure acts as a 2D material which implies that the electric field is constant across the thickness of the layer. This approximation is expected to be valid as long as the thickness of each layer is much smaller than the penetration depth of that material in the THz frequency range.

When using realistic device parameters, the cavity sustains a series of resonances whose current density varies rapidly underneath the metal strips, and is nearly constant, but finite, between the strips. This reflects that, for a given frequency, the wavelength of a plasmon excited in a screened material is much shorter (and q much larger), than a plasmon excited in an unscreened material (where q is smaller).

The cavity conductivity from a bare graphite heterostructure is then proportional to the total current density ($\sigma = j_{tot}/E$) and can be obtained by a RPA (random phase approximation) conductivity approach. The principle of this approach is that an external electric field E_{ext} excites a current which causes an additional electric field which then induces a current j_{ind}, such that the total current in the graphite flake (or other 2D material) amounts to:

$$\begin{aligned} j_{\text{tot,graphite}} &= \sigma_{\text{graphite}}(\omega) E_{\text{ext}} + j_{\text{ind}} \\ &= \sigma_{\text{graphite}}(\omega) E_{\text{ext}} + \alpha\omega E_{\text{ext}} \\ &= \left(\sigma_{\text{graphite}}(\omega) + \alpha(\omega)\right) E_{\text{ext}} \\ &= \sigma_{\text{RPA,graphite}} E_{\text{ext}} \end{aligned} \tag{26}$$

where $\sigma_{\text{RPA,graphite}}$ is the RPA conductivity of the graphite layer and $\alpha(\omega)$ is the response function of transmitted and reflected waves in the transmission line gap region of the graphite layer:

$$\alpha(\omega) = t_2 e^{iq_{\text{un}}x} + r_2 e^{-iq_{\text{un}}x} \tag{27}$$

For $r_2 = t_2$ (see boundary equations (3)) and in the limit of small q, this term simplifies to:

$$\alpha(\omega) = 2r_2 \cos(q_{\mathrm{un}} x) \\ \approx 2r_2. \tag{28}$$

Thus, the current in the middle region is approximately constant and given by: $j_x = 2r_2(\omega)E_0 \cos(q_{un}x) + \sigma_{\mathrm{2D}}(\omega)E_0 \approx (2r_2(\omega) + \sigma_{2D}(\omega))\, E_0$. Finally, this allows the extraction of the measured conductivity as:

$$\boxed{\sigma_{\mathrm{2D,cavity}} = 2r_2(\omega) + \sigma_{2D}(\omega).} \tag{29}$$

The full effective conductivity experienced by the probe, $\sigma_{2D,cavity}$ depends both on the bare conductivity $\sigma_{2D}(\omega)$, but also on reflected and transmitted waves from boundaries of the on-chip cavity. The influence of the boundaries is given by the reflection coefficient $r_2(\omega)$ and is responsible for the appearance of plasmonic resonances. As mentioned in Sec. S0.1, in order to compute the effective conductivity experienced by the probe pulse, we need to scale the 2D cavity conductivity by a filling factor. Comparing full 3D electromagnetic simulations to the analytical model for the cavity geometry parameters, we find very good agreement when choosing a filling factor $\approx$ 200mm^{-1}. The cavity conductivity obtained from the analytical model and the full 3D electromagnetic simulation in Fig. S8, shows comparable results as shown in Fig. S11, providing strong evidence for the validity and accuracy of the analytical model.

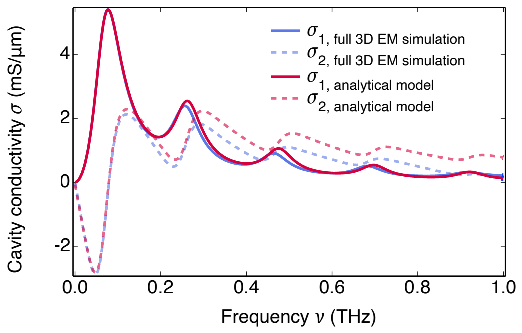

Fig. S11 Comparison of analytical model to a full 3D electromagnetic simulation for a bare graphite cavity: Real and imaginary parts of the cavity conductivity of a bare graphite cavity obtained (as shown in Fig. S8) from a full 3D electromagnetic simulation (blue) and the analytical model (red). The results of the analytical model agree with the full 3D electromagnetic simulation.

S0.3.2 Theoretical description of a heterostructure with two self-cavities: 2D material and graphite gate

The analytical model for the bare graphite cavity was extended to capture the conductivity of a heterostructure with two cavities such as graphene encapsulated in hBN with a graphite gate. A typical cross-section of such a cavity is shown in Fig. S12a. We now expand currents in terms of hybrid, symmetric and antisymmetric modes that form in the graphene and graphite layers and match current densities and potential in each layer.

The model assumes that the heterostructure is perfectly symmetric around a central mirror plane and, as in the case of a single layer introduced above, divides the cavity up into five regions (0 to IV), that are either screened by the transmission line or unscreened. When the graphene is metallic, 2D modulations of the current density, plasmons, can be excited, in addition to the plasmons that are excited in the graphite layer. If the graphene were infinitely far away from the graphite, the resonances of these graphene plasmon modes would be determined by the geometry and screening

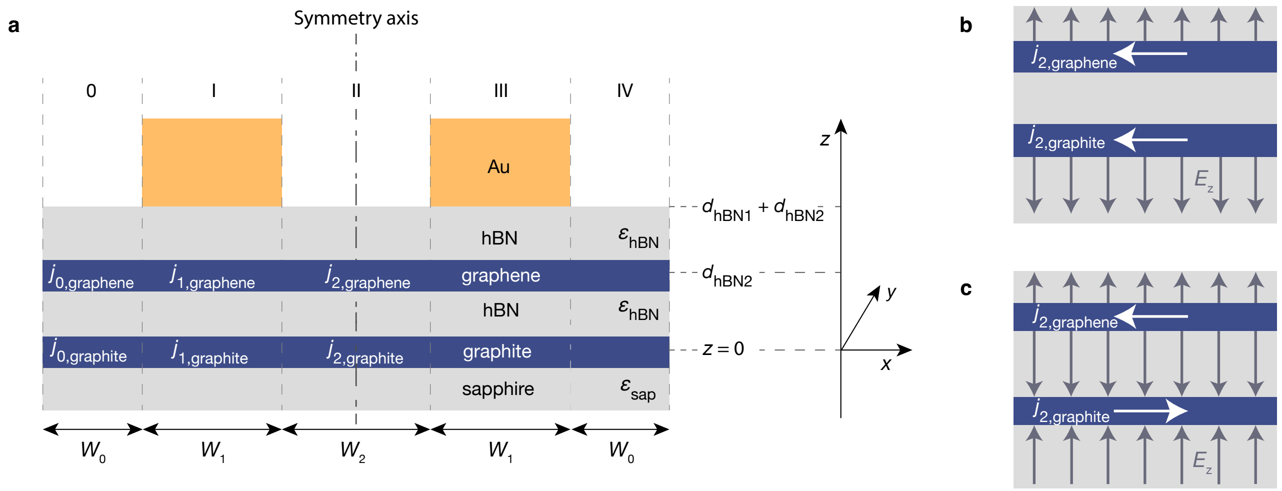

Fig. S12 Symmetric and antisymmetric modes in a typical graphene-graphite cavity: a The cavity can be divided into five regions (0 to IV), that are either screened by the transmission line (I and III) or unscreened (0, II and IV). Current density oscillations j are excited both in the graphene and graphite layer, that are assumed to be continuous along the boundaries between screened and unscreened regions (grey dashed lines). This can lead to the formation of symmetric modes **b**, where current densities oscillate in-phase, or antisymmetric modes **c**, where current densities oscillate out-of-phase.

environment of the cavity in a similar manner to the method described for the bare graphite cavity above.

As the graphene and graphite are brought closer, the charge densities in each layer interact through the Coulomb force and symmetric and antisymmetric modes are formed (see Fig. S12b) [22, 23]. In the symmetric mode, current densities oscillate in-phase in the graphene and graphite, while in the antisymmetric mode, current densities oscillate out-of phase, screening each other and leading to an energetically lower mode (see Fig. S12c).

The symmetric and antisymmetric modes are also dependent on the screening by the metallic transmission lines, such that by solving Maxwell's equations, four plasmon dispersion relations can be found for symmetric and antisymmetric, screened and unscreened modes. The full analytic solutions are too long to be shown here, but an

exemplary plot of the four dispersion relations for a typical graphene cavity is shown in Fig. S13a.

As shown in Fig. S13, the symmetric modes have larger resonance frequencies than their antisymmetric counterparts, which is due to the larger Coulomb interactions for symmetric, in-phase modes. In addition, screened modes have lower resonance frequencies than unscreened modes, as already discussed for single graphite layer cavities in Sec. S0.3.1. The unscreened symmetric mode is the only mode that scales with $\nu_0 \propto \sqrt{q}$ rather than linearly, with resonance frequencies ν_0 that are significantly higher than the resonance frequencies of the other modes for a given plasmon wave vector q (see bright grey-blue modes in Fig. S13).

We note here that we draw the dispersion relations as continuous lines, corresponding to a continuum of 2D plasmon modes that could be sustained in a heterostructure of infinite width. However, due to the finite size of our cavities, only discrete modes with specific momenta can be excited (such as modes which correspond to quarter, three-quarter, and five-quarter wavelength-like modes as shown in Sec. S0.2.2).

When the carrier density in the graphite is much larger than in the graphene, $n_{\text{graphite}} \gg n_{\text{graphene}}$, the dispersion of the symmetric mode is predominantly determined by the graphite (graphite-like), whereas the antisymmetric mode is largely graphene-like. To substantiate this assumption, we plot the dispersion of the symmetric and antisymmetric modes for a range of graphene carrier densities, and compare these to the dispersion relations for a graphite plasmon that is purely screened by the transmission lines at a distance of $d_{\text{hBN1}} + d_{\text{hBN2}}$ and a graphene plasmon that is perfectly screened by both the metal of the transmission lines above and by the graphite below (called double-screened graphene plasmon).

At a graphene carrier density of $n = 0.95 \times 10^{12}$ cm^{-2}, the dispersion relations for a screened graphite plasmon and double-screened graphene plasmon do not overlap with any of the other dispersion relations, as shown in Fig. S13a. However, for very

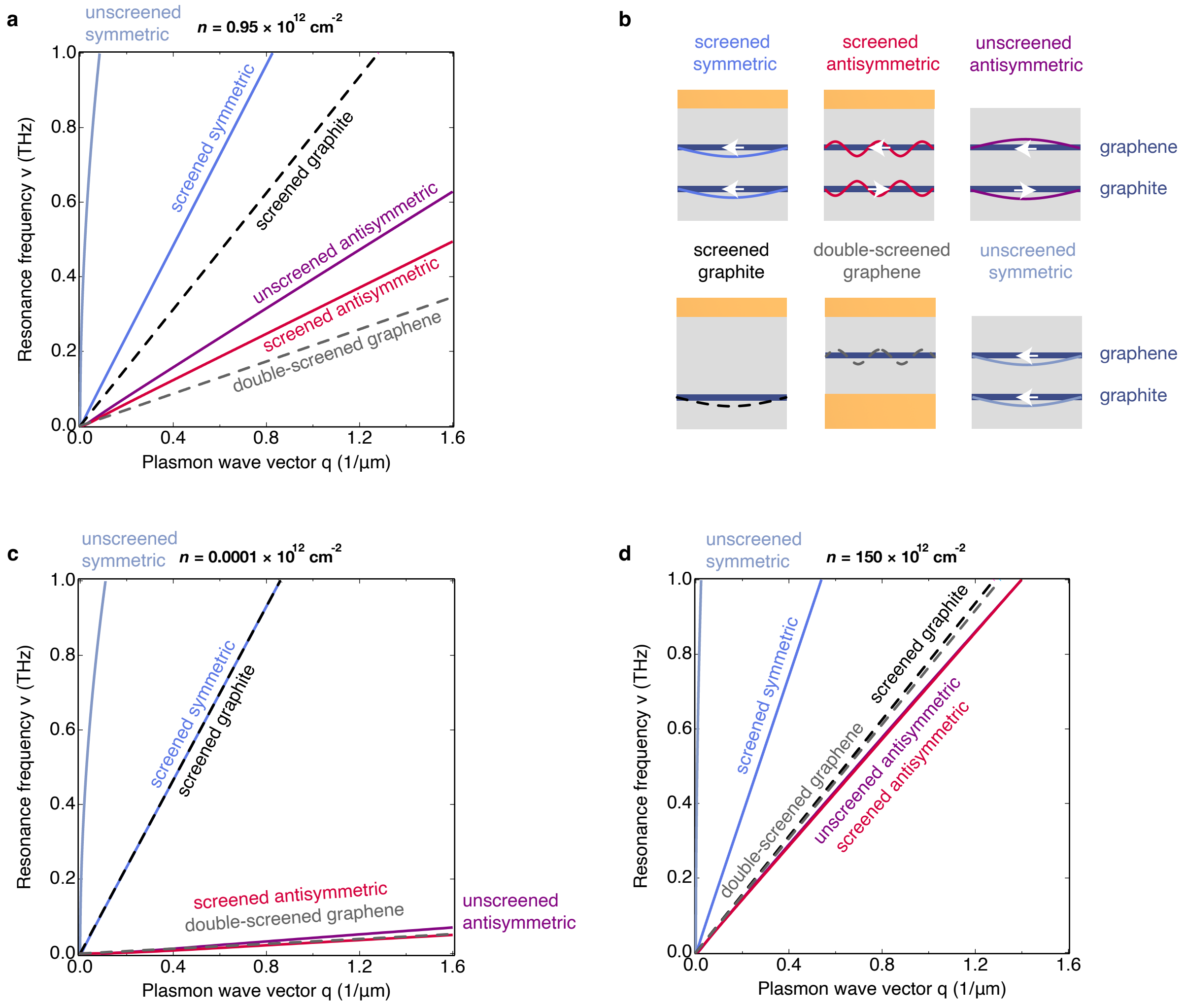

Fig. S13 Dispersion relations of symmetric and antisymmetric modes: a Dispersion relations for screened and unscreened, symmetric and antisymmetric modes, and screened graphite and double-screened graphene modes for a typical graphene cavity with $d_{\mathrm{gr}} = 9$ nm, $d_{\mathrm{hBN1}} = d_{\mathrm{hBN2}} = 9$ nm and $\nu_{\mathrm{3D}} = 118$ THz at a graphene carrier density of $n = 0.95 \times 10^{12}$ cm^{-2}. **b** Legend for other panels with current density and screening sketches for the different modes. **c** Dispersion relations for a small graphene carrier density of $n = 0.0001 \times 10^{12}$ cm^{-2}. In this limit, the screened symmetric modes act like a screened graphite plasmon and the screened antisymmetric mode acts like a double-screened graphene plasmon, screened both by the metal strip above and the graphite underneath (see (b)). **d** Dispersion relations in the limit of a very large graphene carrier density of $n = 150 \times 10^{12}$ cm^{-2}, where screened and unscreened antisymmetric modes lie on top of each other.

small graphene carrier densities, n, the dispersion of the screened symmetric mode (blue) lies on top of the dispersion for the screened 2D graphite plasmon (black dashed line, see Fig. S13b), with a dispersion relation of:

$$\lim_{n\to 0} q_{\text{screened,symmetric}} = \sqrt{\frac{\nu_0^2 \epsilon_{\text{hBN}}}{\nu_{3\text{D}}^2 d_{\text{gr}} (d_{\text{hBN1}} + d_{\text{hBN2}})}}, \tag{30}$$

which is equivalent to Eq. 22 but with an hbn thickness that takes into account both of the insulating layers. This validates that the symmetric mode has mostly graphite character for small graphene carrier densities and is independent of n. While there is a discrepancy between the two dispersion relations when the carrier densities in the graphene and graphite become comparable, as shown in Figs. S13a and c, the impact of this discrepancy on the primary conclusions of this work are minor and discussed in Sec. S0.7.3. For this reason, we identify the symmetric screened mode as the graphite cavity mode in the main text.

For small graphene carrier densities, we find that the dispersion of the screened antisymmetric mode (red) lies on top of the dispersion for a double-screened graphene plasmon (grey dashed line, see Fig. S13b) that is screened from the metal strip at a distance of d_{hBN1} and perfectly screened by the graphite at a distance d_{hBN2}. The dispersion relation of such a double-screened graphene plasmon is:

$$\lim_{n\to 0} q_{\text{screened,antisymmetric}} = \sqrt{\frac{\nu_0^2 \epsilon_{\text{hBN}} \left(\frac{1}{d_{\text{hBN1}}} + \frac{1}{d_{\text{hBN2}}}\right)}{A_{\text{graphene}} \sqrt{n}}}, \tag{31}$$

with $A_{\text{graphene}} = \frac{e^2 v_F}{\epsilon_0 \hbar \sqrt{\pi}}$, where e is the electron charge and v_F the Fermi velocity in graphene. While the two dispersion relations also do not identically coincide for larger graphene carrier densities as shown in Figs. S13a and c, the antisymmetric modes for the uncoupled cavities were still found to scale as $\nu_0 \approx n^{0.25}$, and are thus labeled as graphene modes in the main text.

The unscreened antisymmetric mode, which is found to mediate the coupling between screened symmetric and antisymmetric (screened graphene- and graphite-like plasmons), as discussed below in Sec. S0.9, has a simple solution of:

$$q_{\text{unscreened,antisymmetric}} = \sqrt{\frac{\epsilon_{\text{hBN}}\nu_0^2(A_{\text{gr}}\sqrt{n} + d_{\text{gr}}\nu_{3\text{D}}^2)}{A_{\text{gr}}\sqrt{n}d_{\text{gr}}\nu_{3\text{D}}^2 d_{\text{hBN2}}}}. \tag{32}$$

A final verification of the analytical theory for a heterostructure with two self-cavities can be found by checking the limit of very large graphene carrier densities. In this limit, the screened antisymmetric solution should equal the unscreened antisymmetric solution, because the antisymmetric solution becomes the graphite mode that is now perfectly screened by the graphene. In this case, it should not matter anymore if there is another metal (transmission line) further away. As shown in Fig. S13d, screened and unscreened antisymmetric modes lie on top of one another for $n = 150 \times 10^{12}$ cm^{-2}, further verifying the analytical model.

The formation of two plasmonic modes (symmetric, with in-phase oscillations, and antisymmetric, with out-of-phase oscillations) appears in many different contexts, which we discuss here to connect terminologies across disciplines and provide further intuition. One can think of the formation of the screened (sometimes called 'acoustic') plasmon described earlier as an antisymmetric plasmon oscillation between carriers in the graphene and image charges in the metal. Surface plasmons from two coupled metals separated by a thin dielectric have also been called 'gap' plasmons [24]. Within a single material, symmetric and antisymmetric modes can also form [23]. The Pines Demon mode recently observed in a 3D system [25, 26] corresponds to antisymmetric oscillations of electrons in two bands within the same material (the symmetric mode correspond to the more conventionally known plasmon). In graphene, the recent work by Zhao et al. [27] discusses a hydrodynamic bipolar plasmon, corresponding to antisymmetric oscillations of electrons and holes in the graphene, and a symmetric oscillation of electrons and holes, the hydrodynamic energy wave, or 'demon' mode.

Finally, we note here that the antisymmetric mode, which is orthogonal to the transmission line mode, actually has zero dipole moment and should be considered as a Raman mode that is typically optically silent. However, the step-function change in the screening environment at the boundary between the transmission lines and gap breaks inversion symmetry allowing for a zero-dipole mode to appear in the transmission.

S0.3.3 Influence of hBN anisotropy

In the previous sections, both in the numerical and analytical theory, the permittivity of hBN was assumed to be homogeneous and given by the perpendicular value of hBN, $\epsilon_\perp = 3.7$. In reality, hBN is anisotropic and the dielectric response parallel to graphene is not the same as that perpendicular to graphene, $\epsilon_\parallel \neq \epsilon_\perp$. In this scenario the momentum of the screened 2D plasmon mode of the graphite is given by the equation:

$$\nu^2_{\text{screened,general}} = \frac{v^2_{\text{3D}} d_{\text{gr}} q}{\epsilon_{sap} + \sqrt{\epsilon_\perp \cdot \epsilon_\parallel} \coth\left(\sqrt{\frac{\epsilon_\parallel}{\epsilon_\perp}} q d_{\text{hBN}}\right)}, \tag{33}$$

and as a result in the screened region where d_{hBN} is very small we have the result ($q d_{\text{hBN}} \ll 1$ leads to $\coth\left(\sqrt{\frac{\epsilon_\parallel}{\epsilon_\perp}} q d_{\text{hBN}}\right) \approx 1/\left(\sqrt{\frac{\epsilon_\parallel}{\epsilon_\perp}} q d_{\text{hBN}}\right)$):

$$\nu^2_{screened} = \frac{v^2_{\text{3D}} d_{\text{gr}} d_{\text{hBN}} q^2}{\epsilon_\perp}, \tag{34}$$

while in the unscreened region where $d_{\text{hBN}} \to \infty$, we instead get the expression:

$$\nu^2_{unscreened} = \frac{v^2_{\text{3D}} d_{\text{gr}} q}{\epsilon_{sap} + \sqrt{\epsilon_\perp \cdot \epsilon_\parallel}}. \tag{35}$$

These two expressions show that indeed the screened mode is only sensitive to the out-of-plane dielectric constant. Physically this is due to the fact that the electric field in a screened plasmon points primarily in the z-direction since the material and the coplanar strip form a capacitor-like mode. Deviations from our theory only occur for the unscreened plasmon where the effective dielectric constant experienced from hBN is a geometric mean between the perpendicular and parallel component. The good

agreement with experiments ignoring this effect further emphasizes that the on-chip response is dominated by cavity plasma modes in the screened region.

S0.4 Dependence of the cavity length on the cavity conductivity per micron

The transmitted signal depends on the length of the sample as described by Equation (16) in Section S0.1, where longer samples lead to more of the THz pulse being reflected. However, the effective cavity conductivity does not depend on the length. This is because the THz pulse in the transmission lines is ∼mm long and is approximately uniform along the y-direction of the sample, while at the same time the electric field of the pulse inside the metal traces of the transmission line is oriented almost entirely along the x-direction. Under these two assumptions, the THz pulse launches a spatially patterned current only along the x-direction without any current along the y-direction. As a result the effective cavity conductivity is strongly dependent on the geometry of the sample along the x-direction but independent of the length along the transmission lines. Further evidence of the independence of the

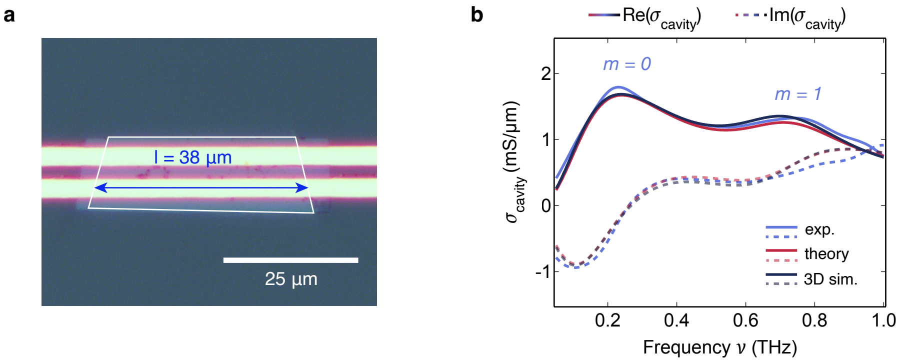

Fig. S14 Data set on a long bare graphite cavity: a Optical micrograph of the cavity. The cavity dimensions are: $l = 38\,\mu\mathrm{m}$, $W_0 = 1\,\mu\mathrm{m}$, $W_1 = 3.33\,\mu\mathrm{m}$, $W_2 = 2.56\,\mu\mathrm{m}$, $d_{\mathrm{gr}} = 5\,\mathrm{nm}$ and $d_{\mathrm{hBN}} = 24\,\mathrm{nm}$. **b** Real and imaginary parts of the cavity conductivity obtained experimentally (blue), from the analytical theory (red) and from full 3D electromagnetic simulations (black). The two resonances stem from the excitation of the $m = 0$ and $m = 1$ cavity modes within the spectral sensitivity range. $C_{\mathrm{filling}} = 360\,\mathrm{mm}^{-1}$.

cavity length on the cavity conductivity per micron is provided by the additional data set shown in Fig. S14. A long bare graphite cavity ($l = 38\,\mu\mathrm{m}$) was measured. Analytical theory and full 3D electromagnetic simulations match with the experimental findings independent of the increased cavity length.

Finally, we note that in the opposite limit where the length becomes extremely small, we expect the results to again be length dependent as the polariton modes excited by the transmission lines will crossover from 2D to 1D polaritons, which have a different dispersion relation.

S0.5 Plasmonic cavity response vs Drude response

We show in the main text that 2D materials such as graphene and thin graphite flakes host standing waves of current density with nodes at the edges of the sample, naturally forming plasmonic self-cavities. The resulting THz conductivity spectra can be fit with a sum of Lorentzians, using the Drude-Lorentz model, where each Lorentzian characterises the resonance of a different plasmonic mode of fixed q. We further show that these cavity modes are highly tunable through the geometry and density of carriers in the material. In this section, we discuss more details of Fig. 2d, which shows when cavity effects have to be considered, and when the conductivity can be interpreted as a Drude response typically expected for measurements of large, metallic samples. This distinction is important for the accurate extraction of intrinsic material properties, and for understanding light-matter interactions in vdW materials. While the analysis here focuses on finite-size effects in metals and semimetals, these results are generalisable to other phases and the formation of polaritons with other collective modes.

The analytical theory can be used to calculate the measured cavity conductivity obtained with on-chip THz spectroscopy (σ_{cavity}), as a function of sample size (W), and intrinsic 2D conductivity of a 2D material, $\sigma_{2\text{D}}$, to determine under which conditions

discrete cavity modes appear in the spectrum and when a Drude response can be detected. Figure S15a shows σ_{2D} (as defined by a Drude lineshape, $\sigma_{2D} = \frac{\epsilon_0 \omega_{3D}^2 d_{gr}}{-i\omega+\gamma}$) of a graphite flake of typical thickness. The value of $\sigma_{2D,1}(\nu = 0)$ corresponds to the DC conductivity, 32.3 mS, and a scattering rate of 0.12 THz. This conductivity is used as an input for the analytical model, which calculates the corresponding cavity conductivity for different sample widths W ($W = 2W_1 + W_2$, $W_1 = W_2$, $d_{hBN} = 25\,\mathrm{nm}$), as shown in Figure S15b,c. For graphite samples of typical dimensions achievable using the current generation of dry-transfer vdW heterostructure fabrication techniques, the cavity conductivity is noticeably different from the intrinsic 2D conductivity, even for a sample width of 300 µm. When the sample lateral dimensions become larger than the THz diffraction limit, for example, 1 mm, a response comparable to σ_{2D} is recovered, with only a small discrepancy occurring at frequencies typically lower than the spectral sensitivity range.

Experimental measurements of a large graphite cavity (see Figure S16) are in agreement with these theoretical findings. The obtained conductivity spectrum from

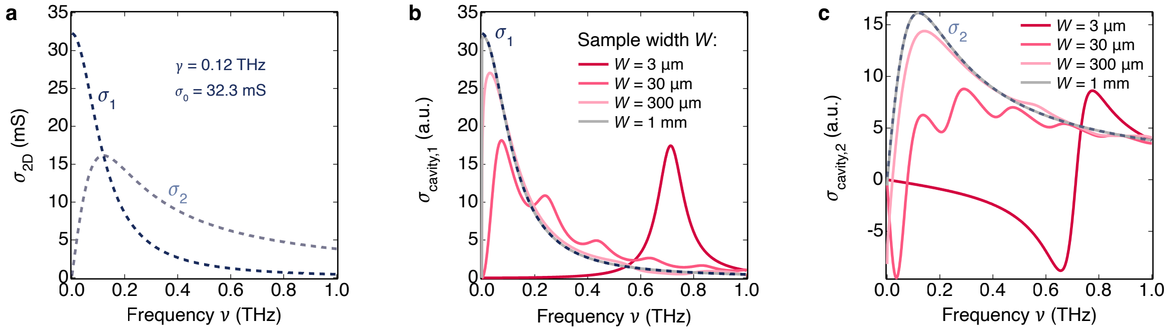

Fig. S15 Drude-like 2D conductivity vs cavity conductivity as function of sample width: **a** 2D conductivity of a typical graphite flake. Real and imaginary parts follow perfectly the Drude-model, which allows to extract the scattering rate γ and DC conductivity σ_0. **b** Real and **c** imaginary parts of the cavity conductivity for different sample widths W ($W = 2 \cdot W_1 + W_2$, $W_1 = W_2$, $d_{hBN} = 25\,\mathrm{nm}$). In the limit of very large samples ($\sim 1\,\mathrm{mm}$, $W \gg \lambda_{THz}$), the cavity conductivity recovers σ_{2D}, with the small different that σ_{cavity} is zero at $\nu = 0$ due to the capacitive sensing of on-chip THz spectroscopy.

this device appeared to be broad, but showed a series of oscillations at low frequencies with peaks that were found to be robust to the analysis/windowing procedure, indicating that the oscillations are due to low-frequency cavity modes. Thus, cavity effects have to be considered, for example to extract linewidths, for relatively large graphene/graphite heterostructures with sizes $\approx 45\,\mu m$.

Fig. 2d demonstrates that both wider sample dimensions and lower 2D conductivities shift cavity resonances to lower frequencies, resulting in a spectrum that deceivingly resembles a Drude response. This is especially critical to account for in the transition zone from small to large enough samples, and from high conductivity to low enough conductivity to show a true Drude response. An example of such a sample in the transition zone is given in Figure S17a,b. A low conducting 2D material with σ_{2D}=4.64 mS and $W = 16\,\mu m$ is chosen as an example. Simulations for a low-conductivity 2D material (σ_{2D}=4.64 mS, $W = 16\,\mu m$) show that overlapping cavity modes can produce a broad peak resembling a Drude response. Without bandwidth below 0.1 THz, this may lead to misinterpretation, as the Drude model fit parameters would deviate significantly from the intrinsic material properties (see Fig. S17c,d).

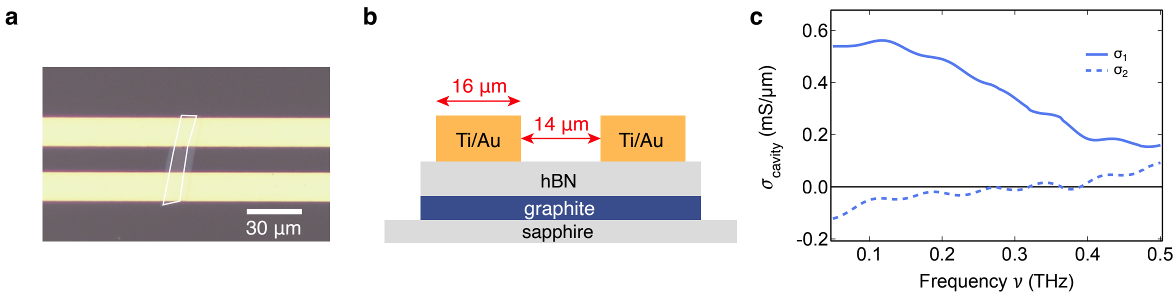

Fig. S16 Cavity conductivity spectrum of a wide graphite cavity: a Optical micrograph of the graphite/hBN heterostructure embedded in a transmission line geometry. **b** Cross-section of the device: $W_1 = 16\,\mu m$ and $W_2 = 14\,\mu m$ are much larger than of the cavities studied in the paper ($d_{gr} = 7$ nm, $d_{hBN} = 32$ nm). **c** Real and imaginary parts of the measured cavity conductivity as function of frequency. The spectrum appears broad as many low-frequency resonances with linewidths larger than their frequency separation contribute to the spectrum.

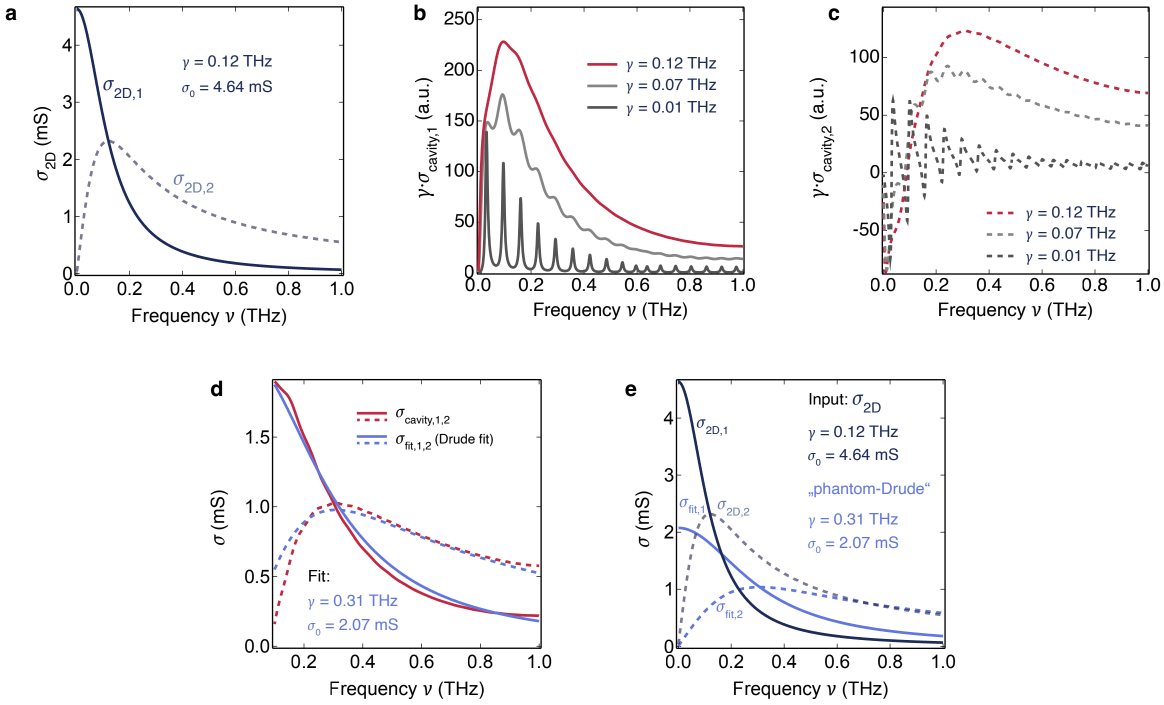

Fig. S17 2D conductivity vs cavity conductivity for a sample with low σ_{2D}: a Real and imaginary parts of the intrinsic 2D conductivity of a 2D material with DC conductivity $\sigma_0 = 4.64\,\text{mS}$. **b** Real and **c** imaginary parts of the cavity conductivity σ_{cavity} for two different scattering rates γ, normalized through a multiplication with the scattering rate to allow seeing the shape of the different linewidth spectra. A series of many low-frequency cavity modes form a broad peak for $\gamma = 0.12\,\text{THz}$. **c** Typically, the spectral resolution in few-mm long on-chip THz spectroscopy circuits is limited to 0.1 THz at low frequencies. In that case, the conductivity spectrum for $\gamma = 0.12\,\text{THz}$ could be mistaken for a Drude response (phantom Drude response). **d** Phantom Drude response in comparison to the input 2D conductivity to the analytical theory. The extracted DC conductivity from the Drude-fit is off by a factor of two and the extracted scattering rate is off by a factor of three. Cavity parameters: $d_{\text{hBN}} = 20\,\text{nm}$, $W_0 = W2 = 3\,\mu\text{m}$, $W_2 = 10\,\mu\text{m}$, $\omega_{3D} = 100\,\text{THz}$ and $d_{2D\ \text{material}} = 1\,\text{nm}$. Note: All panels do not include the scaling factor C_{filling}. However, as C_{filling} is the same for all curves in all shown panels, only the overall amplitudes change, not the relative features.

Consequently, it is essential to carefully evaluate subwavelength spectra that resemble Drude responses, especially when the true peak of the cavity response is lower in frequency than the available spectral bandwidth.

The curve separating the two regimes, Drude response and plasmonic cavity response, in Fig. 2d was calculated with the assumption that the Drude response is still detectable if the conductivity amplitudes of the real parts of σ_{cavity} and σ_{2D} are

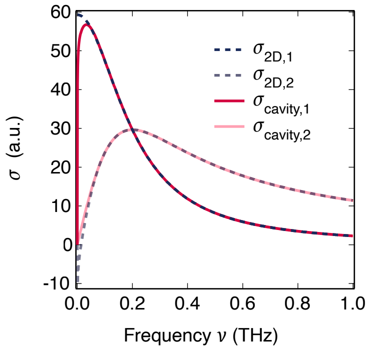

Fig. S18 Cavity response vs Drude response: The curve defining the boundary between a plasmonic cavity response and a Drude response in Fig. 2d was calculated to obtain the maximum 2D conductivity for a given sample width W that still allows a reliable fit of the conductivity obtained with on-chip THz spectroscopy to a Drude model. The curve is defined such that the real parts of σ_{cavity} and σ_{2D} are only different by 10 % at a frequency of 10 GHz and σ_{cavity} and σ_{2D} match nicely for frequencies in the typical spectral sensitivity range ($\approx$ 0.1 THz to 1 THz).

less than 10 % different at a frequency of 10 GHz, as shown in Fig. S18. The curve was calculated for a scattering rate of $\gamma = 0.1$ THz, however, similar results were achieved with various different scattering rates in the range of 0.1 THz to 1 THz.

S0.6 Influence of hybridisation on linewidths

We find that the linewidths of the graphene plasmonic modes detected in the ultrastrong coupling cavity (Fig. 4, Fig. S6) are clearly broadened when compared to the reduced coupling cavity (Fig. 3, Fig. S4). Furthermore, the largest linewidth of the $s = 2$ mode in the ultrastrong coupling cavity is detected at the doping of the maximum coupling strength, indicating that the detected mode broadening could be a further indicator of enhanced coupling.

To obtain a better understanding of the linewidth broadening, we investigated the effect of the graphite cavity linewidth on the linewidth of the graphene plasmonic cavity modes using the analytical theory (see Fig. S19). We found that an increasing graphite cavity linewidth does not affect the linewidth of the graphene modes in a sensing cavity, if the graphite layer is >20 nm and if graphene and graphite layer

are separated by a rather thick hBN flake (see Fig. S19a). If the layers are only separated by a $\approx 20\,\mathrm{nm}$ hBN flake, the graphene-like (antisymmetric) modes broaden slightly through the formation of the hybrid symmetric and antisymmetric modes. This happens as both symmetric and antisymmetric modes have contributions of the scattering rates of both layers, and the effect is most prominent if the distance between the layers is small and if the 2D conductivity of the layers becomes comparable (as for thin graphite or semiconducting gates). However, the effect of mode broadening of graphene plasmonic modes is additionally enhanced in the geometry of an ultrastrong coupling cavity (see Fig. S19b).

Thus, the detected broadening of the graphene modes in Device 3 has a multi-faceted origin. The graphene modes are broadening both through the formation of symmetric and antisymmetric modes in the graphene and graphite layer, that are only

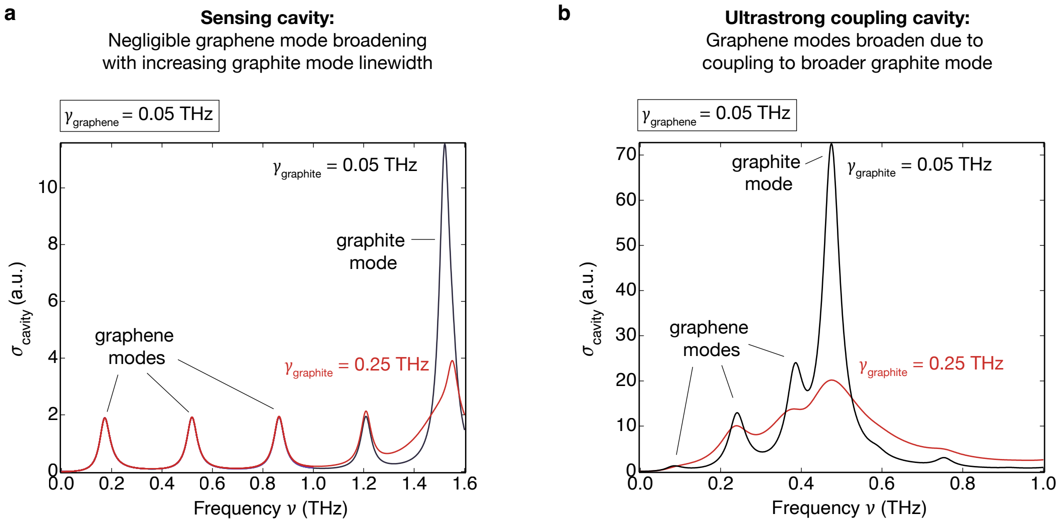

Fig. S19 Increased linewidth of graphene plasmonic modes through ultrastrong coupling **a** In a sensing cavity, the broadening of the graphene plasmonic modes through an increased scattering rate of the graphite mode negligible. Cavity parameters: $W_0 = 0\,\mu\mathrm{m}$, $W_1 = 4\,\mu\mathrm{m}$ and $W_2 = 0.1\,\mu\mathrm{m}$, $d_{\mathrm{gr}} = 30\,\mathrm{nm}$, $d_{\mathrm{hBN1}} = 10\,\mathrm{nm}$, $d_{\mathrm{hBN2}} = 150\,\mathrm{nm}$ and $n = 4 \times 10^{12}\ \mathrm{cm}^{-2}$. **b** In contrast, the graphene plasmonic modes broaden significantly with increasing graphite scattering rates in an ultrastrong coupling cavity. Cavity parameters: $W_0 = 2.2\,\mu\mathrm{m}$, $W_1 = 3.33\,\mu\mathrm{m}$ and $W_2 = 2.6\,\mu\mathrm{m}$, $d_{\mathrm{gr}} = 10.5\,\mathrm{nm}$, $d_{\mathrm{hBN1}} = 21.1\,\mathrm{nm}$, $d_{\mathrm{hBN2}} = 18.3\,\mathrm{nm}$ and $n = 1.24 \times 10^{12}\ \mathrm{cm}^{-2}$.

separated by a thin hBN layer (as the graphite layer has a larger scattering rate) and then additionally through ultrastrong coupling of graphene and graphite modes, which is resonantly enhanced through the antisymmetric modes in the transmission line gap (see Section S0.9.

Refs. [28–30] further suggest that altered linewidths can be a signature of ultrastrong coupling, as dissipation is generally expected to increase through ultrastrong coupling.

To summarize, our theory shows that broadened linewidths in Device 3, can indeed be interpreted as a signature of hybridisation of graphene and graphite self-cavity modes, however due to the competing effects contributing to the linewidth, it is difficult to quantify the broadening due to hybridization.

S0.7 Comparing experimental data, full 3D electromagnetic simulations and analytical model

This section is dedicated to provide further information on the interpretation of the experimental data and analytical theory simulation discussed in Fig. 2, 3, 4 and 5, also by comparing the results to full 3D electromagnetic simulations.

S0.7.1 Device 1

The experimental cavity conductivity data taken on the bare graphite cavity discussed in Fig. 2b,c and Fig. S2 is compared here to full 3D electromagnetic simulations and to the results that the analytical model delivers. Fig. S20 shows that the result from all three approaches are in good agreement.

The full 3D electromagnetic simulations were performed on a cavity with the exact dimensions of the graphite and hBN flake in the vdW heterostructure, including deviations from the shape of a perfect rectangle, assuming a scattering rate of

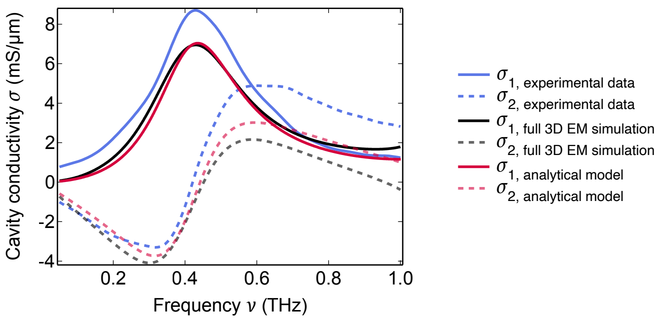

Fig. S20 Experimental data, full 3D electromagnetic and analytical theory simulations for Device 1 of Fig. 2: Real and imaginary parts obtained experimentally, by full 3D electromagnetic simulations and with the analytical model for the cavity geometry of Device 1.

$\tau^{-1} = 0.29\,\text{THz}$, a 3D plasma frequency of $\nu_{3\text{D}} = 121\,\text{THz}$ (0.5 eV) and other geometry parameters as given in Table 1.

The analytical theory simulations were performed assuming the same scattering rate and 3D plasma frequency as for the full 3D electromagnetic simulations, but simplifying the cavity to be perfectly symmetric with $W_0 = 1.9\,\mu\text{m}$. A filling factor of $360\,\text{mm}^{-1}$ was considered to match the amplitudes of the theoretically simulated cavity conductivity with the cavity conductivity obtained from the full 3D electromagnetic simulations.

S0.7.2 Device 2

The cavity of Device 2 was placed slightly asymmetrically underneath the transmission line to avoid covering a bubble with the transmission lines, such that $W_0 = 2.7\,\mu\text{m}$ on one side and $W_0 = 1.3\,\mu\text{m}$ on the other side (see Extended data Fig. 3). For the analytical theory simulations we used the averaged width of $2\,\mu\text{m}$. For all other geometric parameters of the cavity, see Table 1. A scattering rate $\tau^{-1} = 0.14\,\text{THz}$ was chosen both for the graphite and for the graphene layer to capture comparable linewidths to the experimental data.

Comparing amplitudes of the $s = 1$ mode in the experimental data measured at the largest graphene doping to the analytical model for the cavity geometry parameters, we find very good agreement when choosing a filling factor of $180\,\mathrm{mm}^{-1}$.

As discussed in the main text, the reduced power dependencies of the resonance frequencies of the graphene plasmonic modes extracted as function of carrier density for the experimental data is due to the hybridisation of the graphene modes with the graphite cavity mode, which is located at 1.13 THz, outside the spectral sensitivity range. To evidence the role of plasmonic mode hybridisation, we simulated a similar graphene cavity, but where the graphite layer was replaced by an infinitely thick perfect metal layer (see Fig. S21a).

In this geometry, the graphene layer is still screened by a metal located the same distance away as in the cavity with the graphite layer. However, it does not hybridise

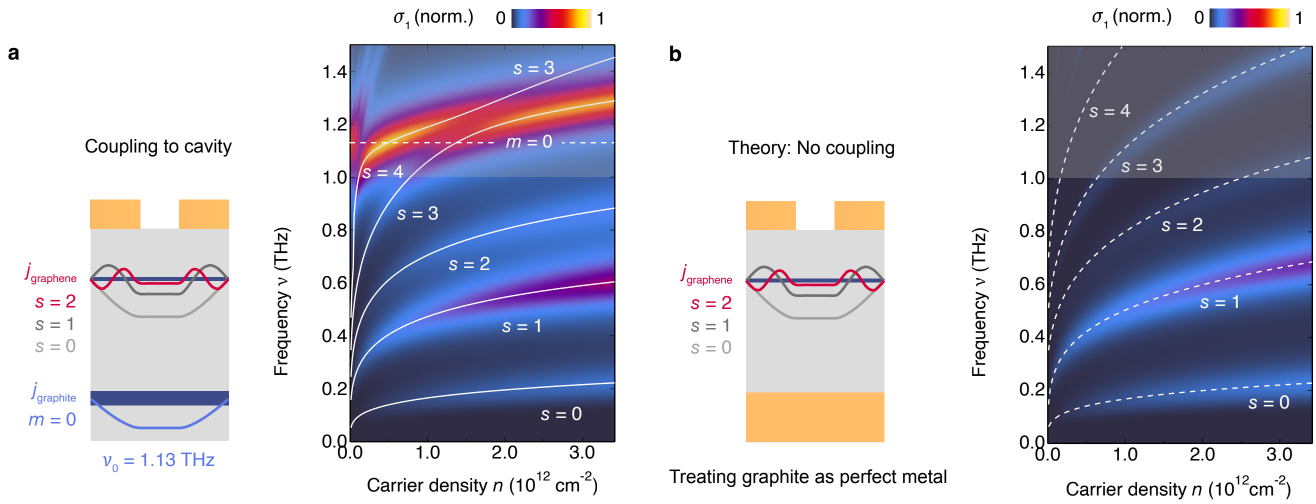

Fig. S21 Analytical theory simulation for Device 2 in Fig. 3: No coupling versus coupling: a Plasmon spectrum as shown in Fig. 3. Through the coupling with the cavity $m = 0$ mode, the graphene $s = 0, 1, 2, ..$ modes are significantly suppressed in resonance frequency when compared to the uncoupled scenario shown in **b**: Graphite of the cavity is treated as a perfect, thick metal forming a cavity with no coupling. All graphene modes ($s = 0, 1, 2, 3, 4$) are undistorted and follow the typical $\nu \propto n^{0.25}$ power laws. The cavity conductivity spectra in (a) and (b) are normalized and comparable between the two panels.

with back gate modes, as the plasmonic resonances would be so far away in frequency that the effect of hybridisation is negligible. Such a simulation reveals a plasmonic spectrum of the cavity with undistorted graphene modes $s = 0, 1, 2, 3, 4$ (see Fig. S21b). When we compare the analytical theory simulation as shown in Fig. 3 and S21a for the graphite cavity we measured to the metal gate cavity spectrum shown in Fig. S21b, the effect of reduced resonance frequencies for the $s = 1, 2, 3$ modes through hybridisation with the $m = 0$ mode becomes visible.

S0.7.3 Device 3

The device parameters given in Table 1 and a scattering rate $\tau^{-1} = 0.03$ THz was used for the analytical theory simulations. The lower scattering rate than experimentally detected linewidth ($\gamma \approx 0.2$ THz, see Fig. S6) was chosen for ease of interpretation and enhanced visibility of the avoided crossing.

The analytical model again compares the results with a completely uncoupled device in the same geometry (see Fig. S22), by treating the graphite layer as a thick perfect metal layer (see Fig. S22b), as discussed in the previous section. The simulation of the uncoupled, thick, perfect metal cavity shows (see Fig. S22a) that all graphene modes, $s = 0, 1, 2, 3$, scale with $\nu \propto n^{0.25}$. In contrast, when the conductivity is calculated using the graphite layer (Fig. S22d), the plasmon resonance frequencies of the different graphene modes are significantly suppressed and a large avoided crossing appears (Fig. S22c).

The normalized coupling strength η can be calculated from the mode splitting of the avoided crossing. However, there are different approaches to doing so that are elaborated on below, and are found to give approximately the same value for η.

The approach chosen in the main paper calculates η at the carrier density of $n_1 = 1.24 \times 10^{12}$ cm^{-2}, where the hybrid modes split equally around the unhybridised graphite mode $m = 0$ mode at $\nu_0 = 0.43$ THz. This approach has the advantage that the normalized coupling strength can be extracted from the experimental data without

comparing the data to the analytical model. When calculating η from the analytical simulations, we find $\eta = \frac{g}{\nu_0} = \frac{50\text{GHz}}{0.43\text{THz}} = 0.11$, marked with the red solid line n_1 in Fig.S22f.

The approach presented above treats the graphite resonance frequency as independent of the graphene carrier density. In reality, the graphite resonance corresponds to

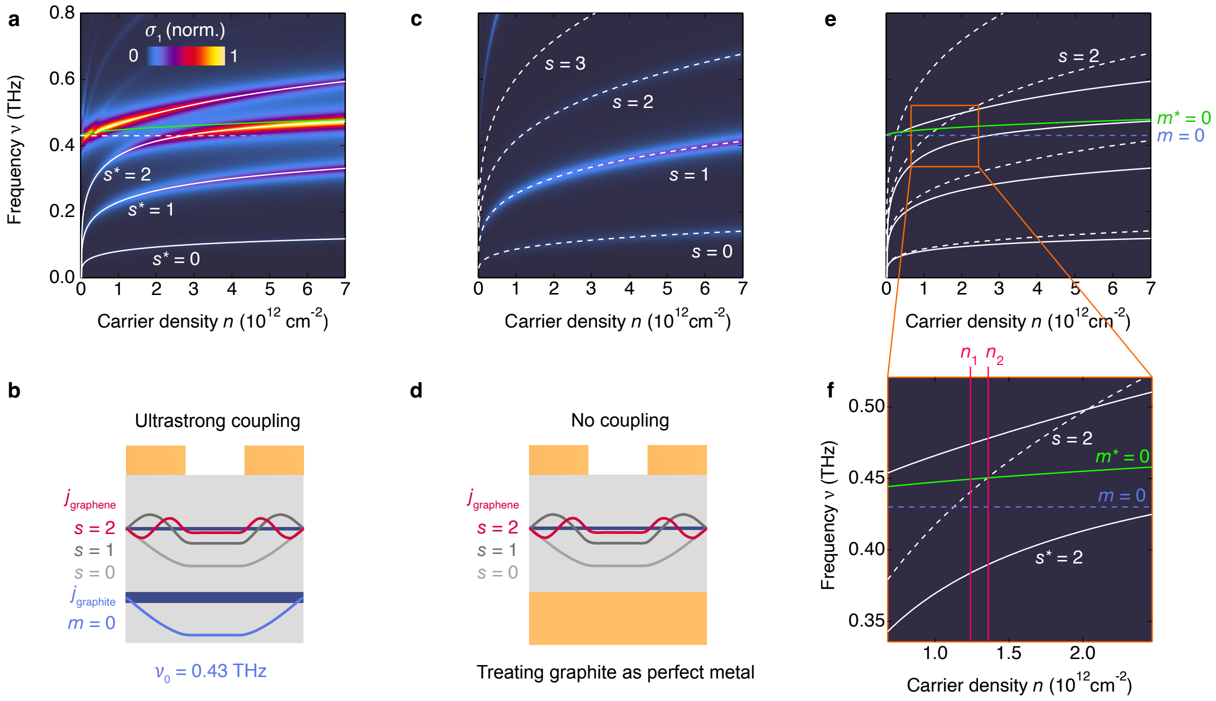

Fig. S22 Analytical theory simulations for Device 3 in Fig. 4: Normalized coupling strength calculation approaches: a Cavity conductivity spectra when the graphene modes couple to the graphite, $m = 0$ mode (**b**). Through the coupling with the graphite cavity, the s modes are suppressed in resonance frequency, forming the hybridised $s^* = 0, 1, 2$ modes and an avoided crossing. **c** Undistorted cavity conductivity spectra for the graphene $s = 0, 1, 2, 3$ modes obtained from the analytical model when treating the graphite layer as a thick perfect metal layer (**d**). The cavity conductivity spectra in (a) and (c) are normalized and comparable between the two panels. For better comparison, the coupled modes (solid lines) of panel (a) and the uncoupled modes (dashed lines) of panel (c) are put on top of one another in panel **e**, in addition to the $m = 0$ mode, which shows the resonance frequency of the graphite in the absence of graphene and the $m^* = 0$ mode, which gives the graphene carrier density dependent graphite resonance frequency of this thin graphite cavity. **f** Zoomed-in carrier density and frequency range of the avoided crossing shown in (e) with the carrier density markers n_1 and n_2 for different normalized coupling strength calculation approaches discussed in the supplementary text.

the symmetric mode in the graphene - graphite heterostructure and added graphene carrier density will slightly blue shift this resonance (see Sec. S0.3.2). For thick graphite flakes ($\gtrsim$ 17 nm), such as for the cavity discussed in Fig. 3, this is a very small correction and the graphite resonance can be assumed to be independent of the number of carriers. However, the device discussed in Fig. 4 has a thinner graphite of roughly 10 nm thickness, where the effect of increasing resonance frequency of the symmetric, graphite-like mode with increasing graphene carrier density becomes visible.

To compute η when accounting for the small change in the symmetric mode resonance frequency with n, the green, $m^* = 0$ curve was calculated with the dispersion relation found by solving Maxwell's equations for a purely screened symmetric mode in a vdW heterostructure consisting of the same graphene, hBN and graphite layers as Device 3. The pure screening of the vdW heterostructure excludes the effect of mode coupling. To preserve the same resonance frequency of the cavity mode in this purely screened cavity as in the coupled, inhomogeneous Device 3, a 2D plasmon wave vector of $q = \frac{2\pi}{5.375 W_1}$ was chosen, with $W_1 = 3.33\,\mu\mathrm{m}$, the same W_1 width as for Device 3. The factor of 5.375 fulfilling the resonance frequency condition can further be interpreted such that the unscreened regions of the cavity increase the wavelength of the screened plasmons from a quarter wavelength mode $\lambda = 4 \cdot W_1$ to $\lambda = 5.375 \cdot W_1$.

If we want to consider the effect of increasing resonance frequency of the graphite-like symmetric $m^* = 0$ mode, the normalized coupling strength should be extracted at a graphene carrier density of $n_2 = 1.36 \times 10^{12}$ cm^{-2}, as the $s = 2$ mode crosses in frequency with the (green) $m^* = 0$ at n_2 (see Fig. S22f). At this carrier density, the modes are split by 0.088 THz, corresponding to $2g$. If we compare this value to the resonance frequency of the $m^* = 0$ mode at this carrier density, which is 0.43 THz, we obtain $\eta \approx 0.10$, which is close to the calculated value using the other approach and still in the ultrastrong coupling regime.

S0.7.4 Sensing cavity simulations for Fig. 5

Full 3D electromagnetic simulations (shown in Fig. S23) were performed for a sensing cavity with the same cavity geometric parameters as the sensing cavity discussed in Fig. 5c (for parameters see Table 1) to demonstrate that this geometry indeed can be used to probe the THz conductivity of vdW heterostructures, without the influence of ultrastrong light-matter interaction. The full 3D electromagnetic simulations were performed using a thin graphite layer (called graphite$_1$) whose simulation parameters were optimized to match the experimental results obtained from 16 measured bare graphite cavity devices, and whose thickness was chosen so that the 2D carrier density is comparable to a graphene carrier density of $n = 1.1 \cdot 10^{12}\mathrm{cm}^{-2}$ of the analytical simulations shown in Fig. 5c.

The full 3D electromagnetic simulations were performed both for the bare graphite$_2$ cavity, and for a dual-cavity heterostructure with an additional, 3 nm thick graphite$_1$ layer assuming a 3D plasma frequency of $\nu_{3\mathrm{D}} = 118\,\mathrm{THz}$ and a scattering rate of $\tau^{-1} = 0.03\,\mathrm{THz}$. The bare graphite cavity mode is centered at a frequency of 856 GHz (see Fig. S23b) with a current density distribution in the graphite$_2$ layer as shown in Fig. S23c.

When the additional graphite$_1$ is placed inside the cavity, the simulations show three additional resonances ($s = 0, 1, 2$) from the graphite$_1$ layer, while the resonance of the plasmonic self-cavity mode does not change significantly when compared to the bare cavity without the graphite$_1$ layer. The current density distributions of the $s = 0, 1, 2$ modes in the graphite$_1$ layer are shown in Fig. S23f, exhibiting mostly undistorted modes with wavelengths $\lambda = 4W_1, (4/3)W_1, (4/5)W_1$.

When the simulations of the cavity shown in Fig. 5c (analytical model) are compared to additional analytical theory simulations where the graphite was replaced by a perfect, thick metal, we find that both cavities exhibit qualitatively similar behaviour. The typical resonance frequency scaling of $\nu \approx n^{0.25}$ is observed. In addition, the

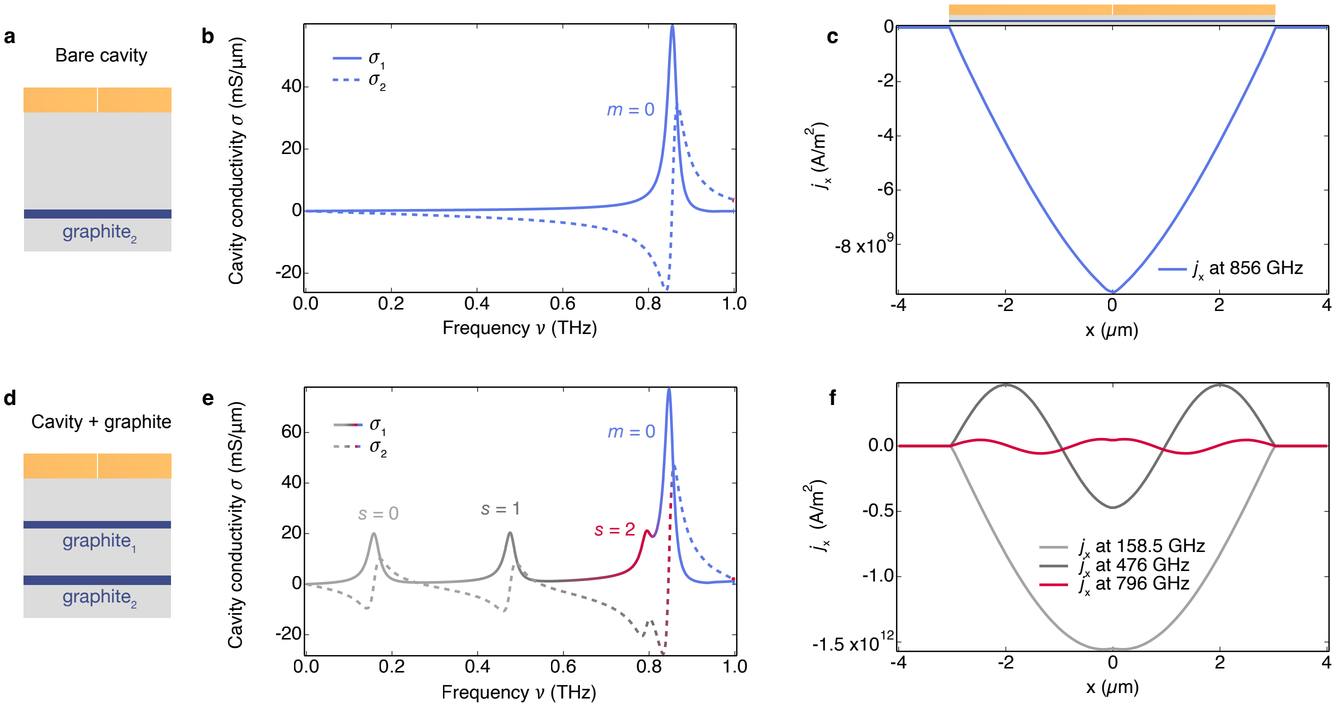

Fig. S23 Full 3D electromagnetic simulations for a sensing cavity design as in Fig. 5c: **a** Design of the simulated bare graphite cavity. **b** Real and imaginary part of the cavity conductivity as function of frequency. The $m = 0$ mode of the bare graphite cavity hosts a resonance centered at 856 GHz. **c** Current density profile in the graphite$_2$ layer at the resonance frequency. **d** An additional graphite layer, graphite$_1$ is introduced into the cavity as a sample. **e** Cavity conductivity spectrum for the cavity with the graphite$_1$. Three additional resonances appear below 1 THz, that correspond to the $s = 0$ (158.5 GHz), $s = 1$ (476 GHz) and $s = 2$ (796 GHz) modes of the graphite$_1$ layer. **f** Current density profiles obtained inside the graphite$_1$ layer at the three resonance frequency positions of the $s = 0, 1, 2$ modes. Cavity parameters: $W_0 = 0\,\mu\text{m}$, $W_1 = 3\,\mu\text{m}$ and $W_2 = 0.01\,\mu\text{m}$, $d_{\text{gr}} = 9\,\text{nm}$, $d_{\text{hBN1}} = 9\,\text{nm}$, $d_{\text{hBN2}} = 93\,\text{nm}$ and $n = 1.1 \cdot 10^{12}\text{cm}^{-2}$.

amplitude of each graphene mode scales with $A_{\text{graphene}} \approx n^{0.5}$. Because the scattering rate τ^{-1} is fixed in the simulations, then the spectral weight of an uncoupled graphene mode scales with $\approx n^{0.5}$.

Thus, both full 3D electromagnetic simulations and analytical theory simulations illustrate that for such a geometry, the cavity can be used to probe the THz conductivity of vdW heterostructures, without the influence of ultrastrong light-matter interaction. We call this cavity a sensing cavity.

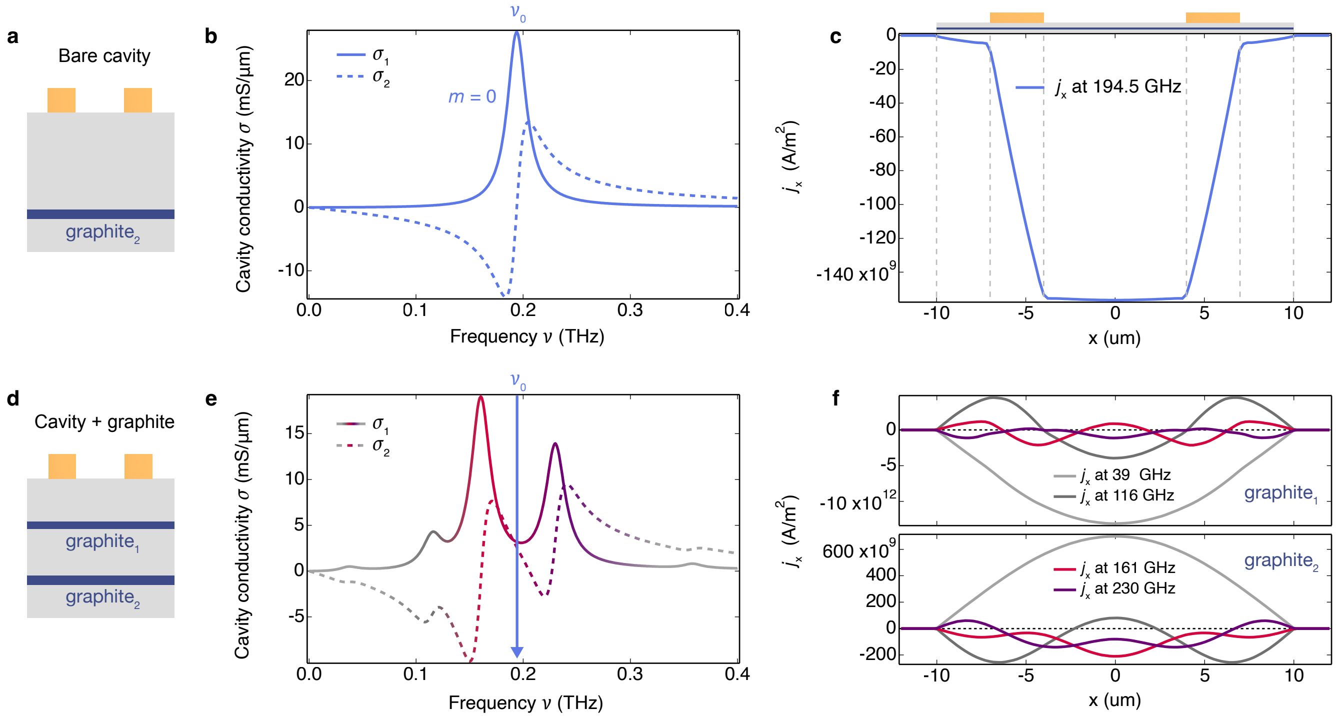

Fig. S24 Full 3D electromagnetic simulations for an ultrastrongly coupled cavity design as in Fig. 5d: a Design of the simulated bare graphite cavity. **b** Real and imaginary part of the cavity conductivity as function of frequency. The $m = 0$ mode of the bare graphite cavity hosts a resonance centered at 194.5 GHz. **c** Current density profile in the graphite$_2$ layer at the resonance frequency. **d** The bare cavity is filled with an additional graphite$_1$ layer, while all other cavity geometry parameters are kept constant. **e** Cavity conductivity spectrum for the cavity with the graphite$_1$. Instead of a resonance at 194.5 GHz, an avoided crossing appears, where the magenta and purple resonances split equally around the self-cavity resonance frequency ν_0. **f** Current density profiles obtained inside the graphite$_1$ (upper panel) layer and inside the graphite$_2$ layer (bottom panel) at the frequencies at which the resonances in (e) appear. Cavity parameters: $W_0 = 3\,\mu\text{m}$, $W_1 = 3\,\mu\text{m}$ and $W_2 = 8\,\mu\text{m}$, $d_{\text{gr}} = 9\,\text{nm}$, $d_{\text{hBN1}} = 9\,\text{nm}$, $d_{\text{hBN2}} = 9\,\text{nm}$ and $n = 0.95 \cdot 10^{12}\text{cm}^{-2}$.

S0.7.5 Ultrastrongly coupled cavity of Fig. 5

Full 3D electromagnetic simulations were performed on a cavity with geometric parameters equal to the cavity with $\alpha = 1$ both for the bare cavity with just a graphite$_2$ layer (see Fig. S24a) and for the cavity with an additional graphite$_1$ layer, placed at the same position as the graphene layer in the cavity discussed in Fig. 5d. A thickness of 0.5 nm and a 3D plasma frequency of 276.7 THz (1.144 eV) were chosen to have the

graphite$_1$ layer comparable to a graphene layer with a carrier density of $n = 0.95 \times 10^{12}$ cm^{-2}, the carrier density at which the avoided crossing occurs. The graphite$_2$ layer has the same thickness of 9 nm and typical 3D plasma frequency of 118 THz (0.49 eV). A scattering rate of $\tau^{-1} = 0.02$ THz was chosen for both graphite layers.

The full 3D electromagnetic simulation for the cavity shows that the resonance of the $m = 0$ mode is centered at $\nu_0 = 194.5$ GHz (see Fig. S24b). The current density distribution j at this resonance frequency is plotted in Fig. S24c, showing that the current density goes to zero at the edges of the dual graphite heterostructure. When the additional graphite$_1$ layer is placed in the cavity (see Fig. S24d), the cavity conductivity spectrum shown in Fig. S24e reveals that the magenta and purple modes form a large avoided crossing and are split equally around ν_0, with $g = 34.5$ GHz, $\nu_0 = 194.5$ GHz, giving $\eta = g/\nu_0 \approx 0.18$, equal to the value obtained from the analytical model for this cavity.

The current density distributions in both the graphite$_1$ and in the graphite$_2$ layer are shown in Fig. S24f. All resonances appear in both graphite layers. The signs of the current densities are opposite, as expected from image charges in the two layers.

S0.8 Multimodal coupling

An avoided crossing is typically illustrated as occurring when *two* modes interact. For example, a constant $m = 0$ mode could cross with an $s = 2$ mode whose frequency changes with carrier density if the modes are uncoupled, as sketched in Fig. S25a. As the coupling is increased, then the modes hybridise and repel each other. A lower plasmon polariton branch which begins with $s = 2$ mode character, saturates at the $m = 0$ mode at large carrier densities (blue dashed line). In contrast the upper plasmon polariton branch begins at the $m = 0$ mode frequency, and gains character of the $s = 2$ mode with carrier density such that it increases in frequency as a function of carrier density at large graphene carrier densities.

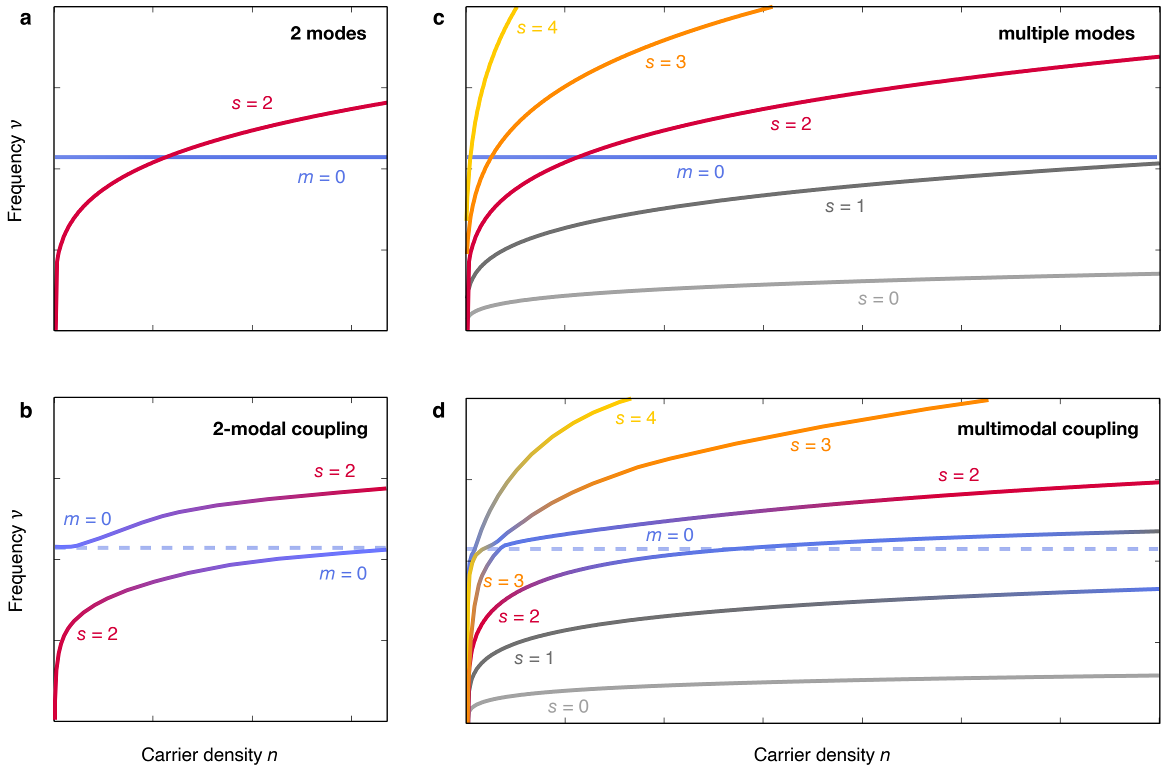

Fig. S25 Sketch of 2-modal coupling vs multimodal coupling: **a** Uncoupled graphene-like ($s = 2$) and graphite cavity $m = 0$ as a function of graphene carrier density n. **b** When the modes hybridise, an avoided crossing appears, and the lower polariton branch saturates to the $m = 0$ frequency. **c** Carrier-density dependence of resonance frequency of a graphite cavity mode and multiple graphene-like modes. **d** When these modes hybridise, level repulsion is observed around their crossing points, and the power-law scales of graphene-like modes are significantly suppressed.

In this work, we observed multimodal coupling between several different graphene-like (antisymmetric) $s = 0, 1, 2, ...$ modes and the graphite-like cavity $m = 0$ mode. In this scenario, the signatures of an avoided crossing are more complex. For the uncoupled case, the $m = 0$ mode is bisected by a sequence of graphene-like antisymmetric s modes, which each scale as a function of $n^{0.25}$ (Fig. S25c). When the $m = 0$ and s modes hybridise, the signatures of such coupling are suppressed power-laws and level-repulsion at the crossing points, as shown in Fig. S25d. A clear saturation of a lower polariton branch to the $m = 0$ line, however, is obscured, as the lower polariton of

one avoided crossing rapidly becomes the upper polariton branch of the next crossing. This effect is observed in Device 3, as shown in Fig. 4, in which the lower polariton branch (labeled $s = 2$ mode) does not saturate at ν_0.

S0.9 Coupling mechanism

In this work, we observed ultrastrong coupling between screened, symmetric graphite plasmons and screened, antisymmetric graphene plasmons. The coupling occurs even though these modes have different momenta, and is maximised when they are resonant in frequency. We further explain the mechanism that couples these modes together below.

We first consider a cavity consisting of a graphene and graphite heterostructure with a homogeneously screened dielectric environment, such as depicted in Fig. S26. Two types of modes can form in this cavity, screened symmetric (graphite-like) and antisymmetric (graphene-like) modes (as discussed in Sec. S0.3.2). Due to the finite size of the cavities, only discrete wavelengths of modes can be admitted in the cavity, which are drawn schematically in Fig. S26c with the blue circle, corresponding to the $m = 0$ bare graphite cavity mode, and grey and red circles, corresponding to graphene $s = 0, 1, 2$ modes. Through tuning of the graphene carrier density, two modes of different momenta, such as the $m = 0$ and $s = 2$ mode can be brought into resonance at a frequency ν_{sc} and at a specific graphene carrier density n_{sc}, as shown in Fig. S26c. However, because the modes have different, well-defined momenta, they are orthogonal to each other and no energy is transferred from one mode to another in this homogeneously screened cavity. As the graphene carrier density is tuned, the screened antisymmetric mode crosses with the screened symmetric mode. The modes become resonant in frequency, and do not form an avoided crossing, as shown in Fig. S26d.

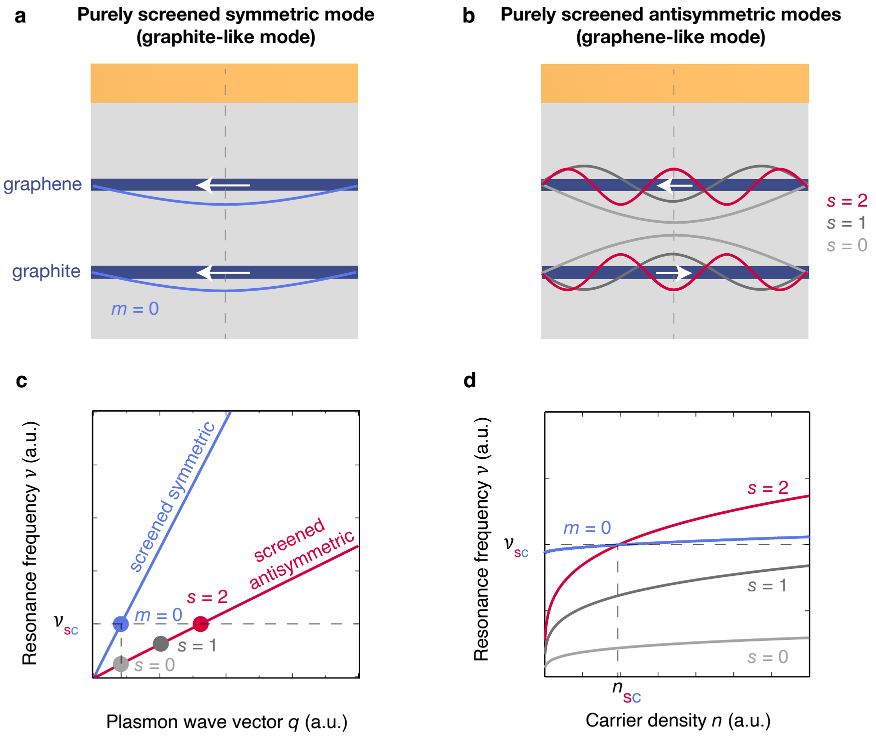

Fig. S26 Cavity with homogeneous screening: a Screened symmetric modes can form in the cavity, where current densities oscillate in-phase in the graphene and graphite layers. For small graphene carrier densities, the symmetric mode behaves as a screened graphite plasmon as shown in Fig. S13. **b** Additionally, screened antisymmetric modes can form, where current densities oscillate out of phase. For small graphene carrier densities, these modes behave as graphene plasmons that are screened by the gold and graphite (see Fig. S13). **c** In the case of homogeneous screening of the cavity, only these two modes can form with the schematically shown dispersion relations at a given graphene carrier density. However, due to the finite size of the cavity, we only excite discrete modes such as indicated by the four circles for the $m = 0$ and $s = 0, 1, 2$ modes. Through tuning of the graphene carrier density, the $s = 2$ mode can be brought into resonance with the $m = 0$ mode, indicated by ν_{sc}. However, these two modes have different momenta and can not couple in a cavity with a homogeneous dielectric environment. **d** This allows the modes to cross in frequency without the formation of an avoided crossing. We sketch the $m = 0$ mode to be slightly increasing in frequency with increasing n to consider the symmetric nature of the graphite cavity mode as discussed in Sec. S0.3.2.

In contrast, a cavity can also consist of a graphene and graphite heterostructure with an inhomogeneously screened dielectric environment, such as depicted in Fig. S27a. When treating the screened and unscreened regions independently, four types of modes can be excited, screened and unscreened, symmetric and antisymmetric modes with dispersion relations drawn in Fig. S27b (see Sec. S0.3.2 for further details). For screened and unscreened cavity regions of finite-size, then again modes can be sustained at only discrete momenta (stars and circles) that depend predominantly on the widths W_1 and W_2.

For two modes to couple together, both momentum and energy must be conserved in the coupling process. As explained above, this is not realisable in a homogeneously screened cavity because the symmetric and antisymmetric modes are orthogonal to each other. However, the symmetric and antisymmetric modes from an inhomogeneously screened cavity (Fig. S27a) contain both screened and unscreened mode character, which results in a much broader momentum distribution for each mode. Consequently, modes, which we nominally denote as having different momenta (such as the $m = 0$ and $s = 2$ modes, labeled by their *screened* character), only have to be brought to similar energies (resonance frequencies) to maximise coupling.

The resonance frequency of the unscreened antisymmetric modes can be varied relative to the resonance frequency of the screened modes by changing the width W_2 for a fixed metal width W_1, which moves the discrete excitations of unscreened antisymmetric modes (purple stars) along the dispersion line (see Fig. S27b). When the purple star aligns with the screened symmetric and antisymmetric modes (blue and red circles) such that $\nu_{\mathrm{unsc},1} = \nu_{\mathrm{sc}}$, a maximum coupling strength can be achieved (see Fig. S28). Therefore, the unscreened antisymmetric mode can be thought of as the spring in Fig. S27a and Fig. 1 of the main text, which mediates the coupling between screened symmetric and antisymmetric modes.

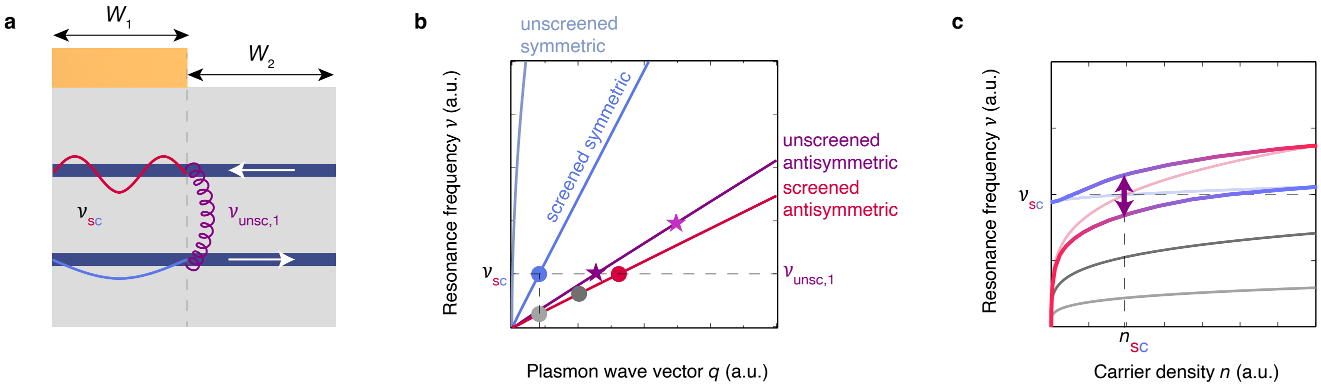

Fig. S27 Cavity with inhomogeneous screening: a The cavity consists of screened and unscreened vdW heterostructure regions. When treating screened and unscreened regions independently, screened symmetric (blue) and antisymmetric (red) modes can form underneath the metal strip, that can couple through the resonant unscreened antisymmetric mode at the boundary. **b** Dispersion relations for screened and unscreened, symmetric and antisymmetric modes. The antisymmetric unscreened mode can have a resonant mode at $\nu_{\text{unsc},1}$ as indicated by the purple star, that is close or equal in resonance frequency to the screened antisymmetric mode (red circle). Due to the presence of localized charge hot spots at the screened/unscreened boundaries that provide a wide distribution of available momentum states, screened symmetric and antisymmetric modes can couple at the boundaries when the unscreened antisymmetric mode approaches similar resonance frequencies/momenta. **c** An avoided crossing forms as a result of the mode coupling (shown schematically).

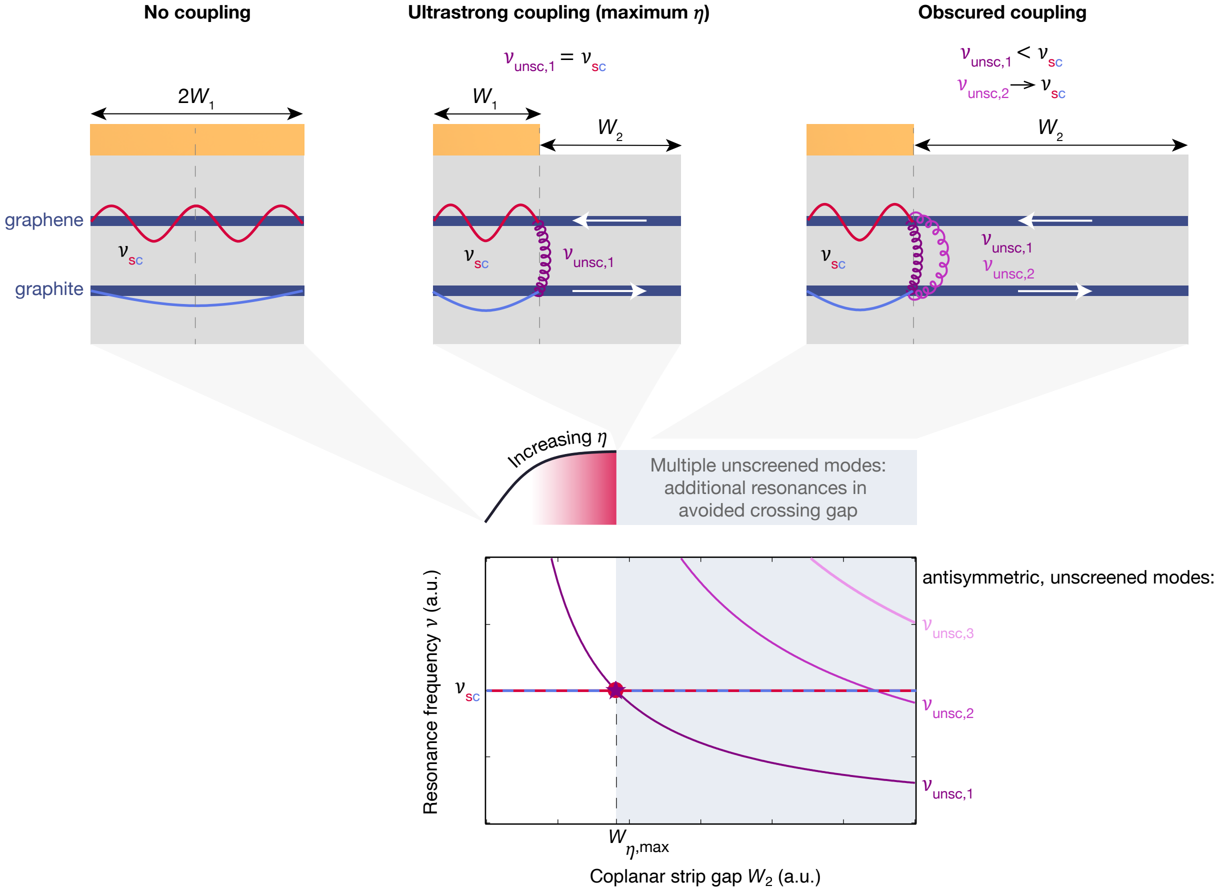

Fig. S28 Schematic illustration of the coupling strength tuning mechanism: A homogeneously screened cavity does not allow coupling of modes of different momenta, as shown in Fig. S26, such that $\eta = 0$. Once there is a tiny unscreened gap between the two metal strips, modes of different momenta can couple at the boundaries between screened and unscreened regions, as a wide range of momenta are available through localized charge hot spots at the screened/unscreened boundaries. The coupling is resonantly enhanced through the resonance frequency of the antisymmetric unscreened mode (purple spring), which can be tuned with the gap width W_2. In the W_2-range between 0 and $W_2 = W_{\eta,\mathrm{max}}$, the purple unsc,1 mode ($\lambda = W_2$) is pushed continuously closer to ν_{sc}, which increases the normalized coupling strength η (bottom panel). A maximum coupling strength is reached when $\nu_{\mathrm{unsc},1} = \nu_{\mathrm{sc}}$, which happens at $W_2 = W_{\eta,\mathrm{max}}$. However, for further increasing W_2, higher harmonics of the unscreened modes (bright purple $\nu_{\mathrm{unsc},2}$ mode with $\lambda = 1/2 \cdot W_2$ and brightest purple $\nu_{\mathrm{unsc},3}$ mode with $\lambda = 1/3 \cdot W_2$) approach ν_{sc}, that then additionally couple to the resonant screened modes (top right panel) leading to additional resonances in the unscreened region, which obscures the coupling.

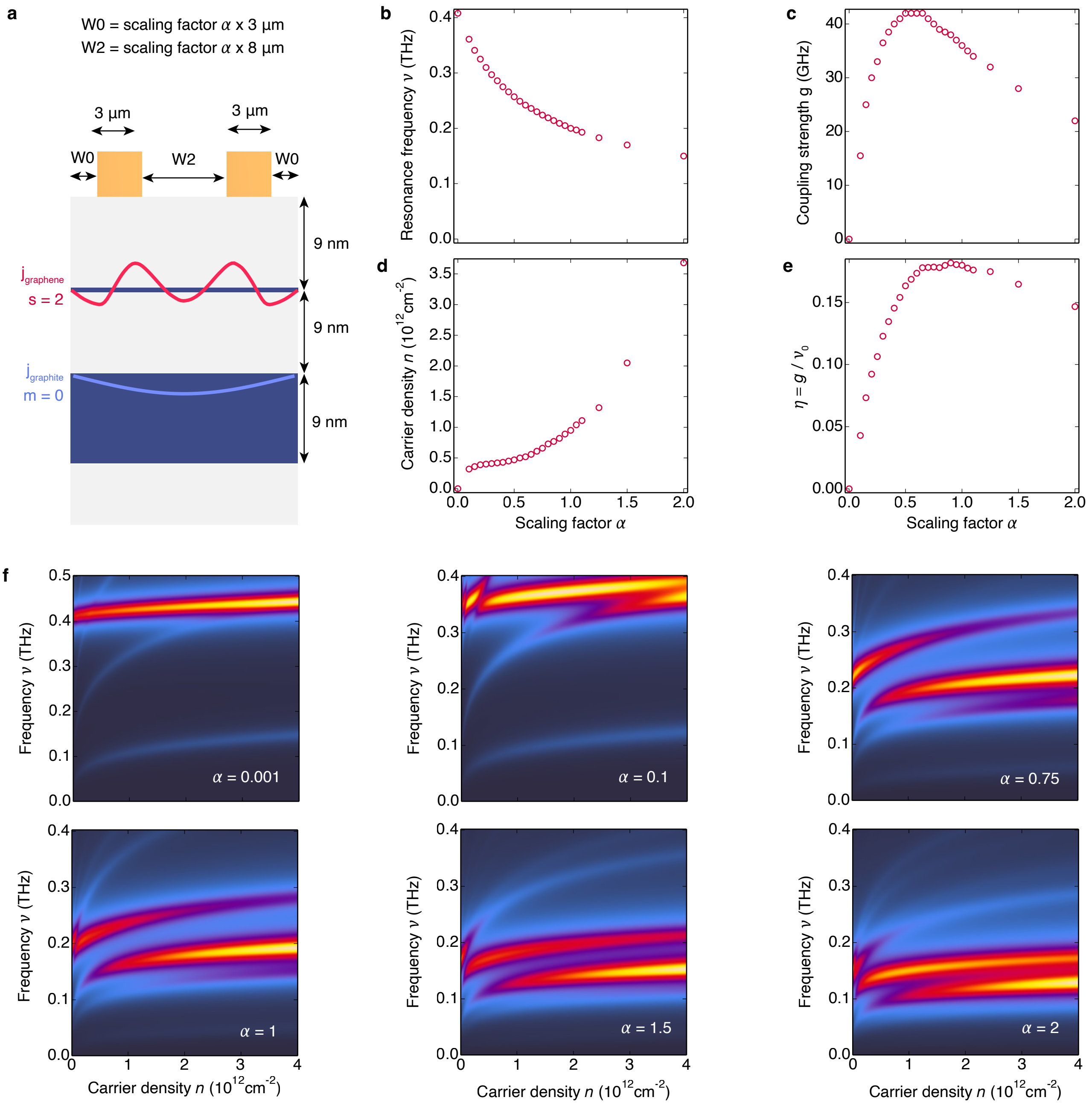

Fig. S29 Additional information for Fig. 5e: a Cross-section of the cavity simulated in Fig. 5b,d,e. The widths $W_0 = 3\,\mu\text{m}$ and $W_2 = 8\,\mu\text{m}$ are scaled by a factor α. **b-e** Resonance frequency ν, coupling strength g, carrier density n and normalized coupling strength η for the avoided crossing between the $s = 2$ and $m = 0$ modes as function of α. **f** Selection of analytical theory simulations for different values of α. Note that the cavity conductivity amplitudes are normalized of the individual panels to the maximum conductivity, but are not comparable between the panels. The maximum conductivity of the panel for $\alpha = 2$ is ≈ 200 times larger than of the panel for $\alpha = 0.001$, neglecting filling factors.

Thus, increasing W_2 relative to W_1 serves as a coupling strength tuning knob until $\nu_{\text{unsc},1} = \nu_{\text{sc}}$ at a width $W_2 = W_{\eta,\text{max}}$ (see Fig. S28). When the transmission line gap W_2 is further increased, the situation becomes more complicated. In addition to the unscreened unsc,1 mode (purple), higher order plasmons of the unscreened region (bright purple unsc,2 mode, brightest purple unsc,3 mode in Fig. S28) start to appear in the resonance frequency range which also couple to the screened modes. This obscures the coupling as more resonances are squeezed into the specific frequency range.

Fig. S29 provides additional information on Fig. 5e, where the normalized coupling strength as function of scaling factor α is shown. α scales the widths of the unscreened regions $W_0 = \alpha \cdot 3\,\mu\text{m}$ and $W_2 = \alpha \cdot 8\,\mu\text{m}$. As plotted in Fig. S29, the coupling strength g and the normalized coupling strength η measured between the $m = 0$ and the $s = 2$ mode appears to decrease for $W_2 > 8\,\mu\text{m}$ (for $\alpha > 1$) utilizing the normalized coupling strength calculation protocol of this work, which can be ascribed to the obscured coupling mechanism of higher harmonics of the unscreened antisymmetric modes, as described above.

S0.10 Quantum fluctuations in the ultrastrong coupling regime

One of the primary motivations for achieving the ultrastrong coupling regime in light - matter hybrid systems is the ability to modify quantum properties of the equilibrium state, such as modifying quantum fluctuations and generating squeezed modes in the ground state [31]. The ability to modify fluctuations of collective modes underpins a plethora of theoretical proposals for realising cavity induced phase transitions in 2D materials. In this section, we theoretically map the graphene - graphite plasmons on a quantum model of two harmonic oscillators, X_1 and X_2 respectively, to illustrate the modification of fluctuations one can expect with the ultrastrong coupling we report. The Hamiltonian of two coupled Harmonic oscillators is given by:

$$H = \frac{\omega_1^2}{2} X_1^2 + \frac{\omega_2^2}{2} X_2^2 + \lambda \tanh\left(\frac{\omega_1}{\omega_0}\frac{\omega_2}{\omega_0}\right) X_1 X_2 + \frac{P_1^2}{2} + \frac{P_2^2}{2}, \tag{36}$$

In this minimal model, X_1 is a generalised quantum coordinate representing the graphite plasmon, and we set $\omega_1 = \omega_c$ to the graphite plasma frequency. X_2 is the graphene plasmon coordinate, and we set ω_2 to the graphene plasma frequency. The interaction between them arising from the coupling mediated by the inhomogeneous on-chip geometry, as described above, is captured by the parameter λ. The $\tanh\left(\frac{\omega_1\omega_2}{\omega_0^2}\right)$, is a function that is added to mitigate unstable behaviour present in this model when either ω_1 or ω_2 is too small. At high frequencies, this prefactor becomes 1. We take $\omega_0 = \omega_c$, as then when $\omega_1 = \omega_2 = \omega_c$, i.e. on resonance, this factor is again equal to 1, such that λ corresponds to the splitting when on resonance. In Fig. S30(a), the dispersion of the coupled system is shown. λ determines the level repulsion when the two modes are on resonance. To fit a splitting of $0.2 \cdot \omega_c$ found in experiments, we take $\lambda = 0.27 \cdot \omega_c^2$.

Quantum effects are introduced by imposing the canonical commutation relations, $[\hat{X}_1, \hat{P}_1] = i$ and $[\hat{X}_2, \hat{P}_2] = i$. Diagonalising the system allows us to compute both quantum fluctuations and squeezing due to the coupling. In Fig. S30b, we plot the relative change of the quantum fluctuations of the cavity mode relative to the undoped case, $\frac{\langle \hat{X}_1^2(g)\rangle - \langle \hat{X}_1^2(g=0)\rangle}{\langle \hat{X}_1^2(g=0)\rangle}$. Changes to the fluctuations show how ultrastrong coupling can influence the ground state of the cavity. To determine the amount of squeezing, we define the creation operator of the cavity (graphite plasmon), as $\hat{a}_1^\dagger = \frac{\sqrt{\omega_1}\hat{X}_1 - i\hat{P}_1/\sqrt{\omega_1}}{\sqrt{2}}$, which obeys bosonic commutation relations, $[a_1, a_1^\dagger] = 1$. In terms of this operator, we can compute the amount of squeezing through $\langle a_1^\dagger a_1^\dagger \rangle$ as plotted in Fig. S30c. When this object is non-zero it shows that the ground state of the system is no longer a vacuum in the original cavity creation operators. The presence of squeezing is also a consequence of breaking of the so-called rotating wave approximation (RWA) often discussed in the literature in relation to ultrastrong coupling[32].

We close this section by saying that based on this simple model, we expect significant modification of quantum fluctuations in the ultrastrong coupling regime. However, to compute from first principles how fluctuations of concrete observables such as the electric field or current are affected, a full quantum electrodynamics approach needs to be implemented in future work.

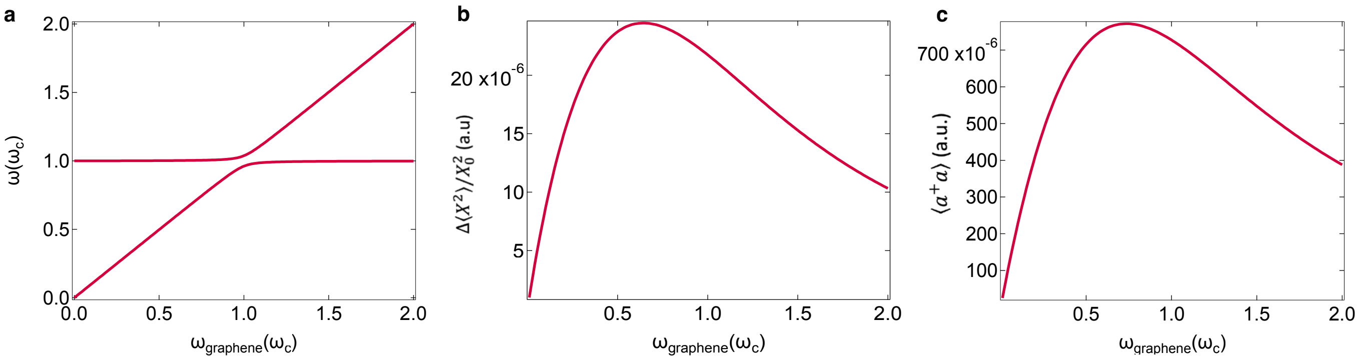

Fig. S30 Consequences of ultrastrong light-matter coupling: a Dispersion of the cavity and graphene collective modes coupled using Eq. 36. When the graphene mode is resonant with the cavity, an avoided crossing appears **b** In such a scenario, the quantum fluctuations of the cavity mode are modified in the ground state through this coupling. **c**. The ultrastrong coupling generates a squeezed state of graphite plasmons which is responsible for the change in the quantum fluctuations of the coupled ground state. The correspondence between squeezing and the change in quantum fluctuations can be graphically seen by the fact that the curves are proportional to each other.

References

[1] Gallagher, P. *et al.* Quantum-critical conductivity of the Dirac fluid in graphene. *Science* **364**, 158–162 (2019).

[2] M. Born, E. W. *Principles of optics* (Pergamon Press Ltd., 1970).

[3] Schulte, B. F. *Ultrafast Transport in Tunable Graphene Heterostructures*. Phd thesis, University of Hamburg (2022).

[4] Ismail, N., Kores, C. C., Geskus, D. & Pollnau, M. Fabry-Pèrot resonator: spectral line shapes, generic and related Airy distributions, linewidths, finesses, and performance at low or frequency-dependent reflectivity. *Opt. Express* **24**, 16366–16389 (2016). URL https://opg.optica.org/oe/abstract.cfm?URI=oe-24-15-16366.

[5] Di Paolo, F. *Networks and devices using planar transmissions lines* (CRC Press, 2018).

[6] Neu, J. & Schmuttenmaer, C. A. Tutorial: An introduction to terahertz time domain spectroscopy (THz-TDS). *Journal of Applied Physics* **124** (2018).

[7] Nuss, M. C. & Orenstein, J. Terahertz time-domain spectroscopy. *Millimeter and submillimeter wave spectroscopy of solids* 7–50 (2007).

[8] Jenkins, M. A. Algorithm 493: Zeros of a real polynomial [c2]. *ACM Transactions on Mathematical Software (TOMS)* **1**, 178–189 (1975).

[9] McIver, J. W. *et al.* Light-induced anomalous Hall effect in graphene. *Nature physics* **16**, 38–41 (2020).

[10] Laturia, A., Van de Put, M. L. & Vandenberghe, W. G. Dielectric properties of hexagonal boron nitride and transition metal dichalcogenides: from monolayer to

bulk. *npj 2D Materials and Applications* **2**, 6 (2018).

[11] Systemes, D. CST simulation software. https://space.mit.edu/RADIO/CST_online/mergedProjects/3D/special_struct/special_struct_layer_parameters_general.htm (2020).

[12] Scheuch, M. *Strong modulation of THz optical properties of layered materials by thermal and ultrafast heating*. Ph.D. thesis, Freie Universität Berlin (2012).

[13] Yoon, H., Yeung, K. Y., Kim, P. & Ham, D. Plasmonics with two-dimensional conductors. *Philosophical Transactions of the Royal Society A:Mathematical, Physical and Engineering Sciences* **372**, 20130104 (2014).

[14] Alonso-González, P. *et al.* Acoustic terahertz graphene plasmons revealed by photocurrent nanoscopy. *Nature nanotechnology* **12**, 31–35 (2017).

[15] Economou, E. N. Surface Plasmons in Thin Films. *Phys. Rev.* **182**, 539–554 (1969). URL https://link.aps.org/doi/10.1103/PhysRev.182.539.

[16] Laturia, A., Van de Put, M. L. & Vandenberghe, W. G. Dielectric properties of hexagonal boron nitride and transition metal dichalcogenides: from monolayer to bulk. *npj 2D Materials and Applications* **2**, 6 (2018). URL https://doi.org/10.1038/s41699-018-0050-x.

[17] Svintsov, D. A. & Alymov, G. V. Refraction laws for two-dimensional plasmons. *Physical Review B* **108**, L121410 (2023).

[18] Lundeberg, M. B. *et al.* Tuning quantum nonlocal effects in graphene plasmonics. *Science* **357**, 187–191 (2017).

[19] Gonçalves, P. *et al.* Quantum surface-response of metals revealed by acoustic graphene plasmons. *Nature Communications* **12**, 3271 (2021).

[20] Reserbat-Plantey, A. *et al.* Quantum nanophotonics in two-dimensional materials. *ACS Photonics* **8**, 85–101 (2021).

[21] Boroviks, S. *et al.* Extremely confined gap plasmon modes: when nonlocality matters. *Nature Communications* **13**, 3105 (2022).

[22] Hwang, E. & Sarma, S. D. Plasmon modes of spatially separated double-layer graphene. *Physical Review B* **80**, 205405 (2009).

[23] Sarma, S. D. & Madhukar, A. Collective modes of spatially separated, two-component, two-dimensional plasma in solids. *Physical Review B* **23**, 805 (1981).

[24] Ding, F., Yang, Y., Deshpande, R. A. & Bozhevolnyi, S. I. A review of gap-surface plasmon metasurfaces: fundamentals and applications. *Nanophotonics* **7**, 1129–1156 (2018).

[25] Husain, A. A. *et al.* Pines' demon observed as a 3D acoustic plasmon in Sr_2RuO_4. *Nature* **621**, 66–70 (2023).

[26] Pines, D. Electron interaction in solids. *Canadian Journal of Physics* **34**, 1379–1394 (1956).

[27] Zhao, W. *et al.* Observation of hydrodynamic plasmons and energy waves in graphene. *Nature* **614**, 688–693 (2023).

[28] Canales, A., Karmstrand, T., Baranov, D. G., Antosiewicz, T. J. & Shegai, T. O. Polaritonic linewidth asymmetry in the strong and ultrastrong coupling regime. *Nanophotonics* **12**, 4073–4086 (2023). URL https://doi.org/10.1515/nanoph-2023-0492.

[29] Houdré, R., Stanley, R. P., Oesterle, U. & Weisbuch, C. Strong coupling regime in semiconductor microcavities. *Comptes Rendus Physique* **3**, 15–27 (2002). URL

https://www.sciencedirect.com/science/article/pii/S1631070502012999.

[30] Törmä, P. & Barnes, W. L. Strong coupling between surface plasmon polaritons and emitters: a review. *Reports on Progress in Physics* **78**, 013901 (2014). URL https://dx.doi.org/10.1088/0034-4885/78/1/013901.

[31] Scully, M. O. & Zubairy, M. S. *Quantum optics* (Cambridge university press, 1997).

[32] Frisk Kockum, A., Miranowicz, A., De Liberato, S., Savasta, S. & Nori, F. Ultrastrong coupling between light and matter. *Nature Reviews Physics* **1**, 19–40 (2019).
